# Supplementary material for: Determinants of adult sedentary behavior and physical inactivity for the primary prevention of diabetes in historically disadvantaged communities: A representative cross-sectional population-based study from Reunion Island
Source: PLoS One. 2024 Aug 13;19(8):e0308650. doi: 10.1371/journal.pone.0308650 (PMC11321555; doi:10.1371/journal.pone.0308650)
Supplement: S1 Appendix — (PDF) [file pone.0308650.s001.pdf]

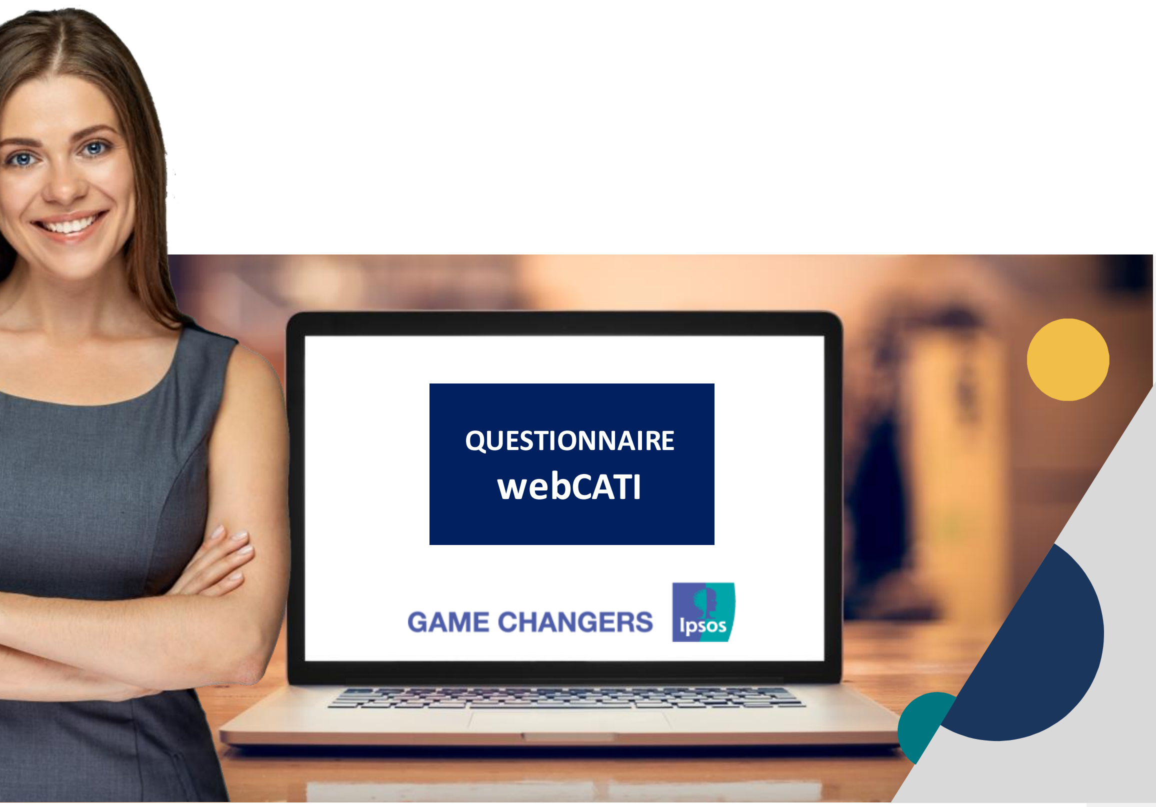

Enquête régionale sur les pratiques physiques et sportives à La Réunion

20-019764-01

Université de la Réunion

VOTRE CONTACT IPSOS

Cybina CHIV / Margaux PASSIER

cybina.chiv@ipsos.com / margaux.passier@ipsos.com

Tel +33 1 41 98 96 77 /+33 1 41 98 99 67

Ce questionnaire a été rédigé selon les standards Qualité d'Ipsos France.
Il a été relu et validé par :
Valérie Blineau, Directrice adjointe du département des Grandes Enquêtes

afaq
RGPD

26/08/2021

INFO FICHIER A RECUPERER

IDENT
SEXE_CIBLE
JOUR_NAISSANCE_CIBLE
MOIS_NAISSANCE_CIBLE
ANNE_NAISSANCE_CIBLE
ADRESSE
COMPLEMENT_ADRESSE
CODE_POSTAL
LIBELLE_COMMUNE

MICRO_REGION – QUOTAS A SUIVRE

1 MR1 - sud
2 MR2 - est
3 MR3 - nord
4 MR4 - ouest

INDIC_QPV - – QUOTAS A SUIVRE

1 0
2 1

TOP DEBUT QUESTIONNAIRE

IDENTIFICATION ARRIVEE CATI

AFFFICHAGE FIXE IDENT SUR CATI EN BAS A GAUCHE

DATE

VERIFNAISSCATI

Enq : vérifier la date de naissance

PROG : FORMAT DATE JJ/MM/AAAA

REPORT DES INFORMATIONS FICHIER A VALIDER OU CORRIGER

JNAIS

MIN : 01, MAX : 31

MNAIS

MIN : 01, MAX : 12

ANAIS

MIN : 1900, MAX : ANNEE EN COURS

SINGLE

VERIFSEXE

Enq : coder le sexe du répondant

REPORT DES INFORMATIONS FICHIER A VALIDER OU CORRIGER

- 1. Un homme

- ❍ 2. Une femme
- ❍ 3. (ne sait pas, ne souhaite pas répondre)

TOP FIN INTRO CATI

RECODE : AGE (AGE DU REPONDANT)
CALCULER AGE AVEC VERIFNAISSCATI (A FAIRE SUR MOIS/ANNEE)
SI VERIFNAISSCATI=VIDE ALORD CALCULER AGE AVEC INFO FICHIER ANNE_NAISSANCE_CIBLE

RECODE : SEXE (SEXE DU REPONDANT)
SI VERIFSEXE RENSEIGNEE
SEXE=1 SI VERFISEXE=1
SEXE=2 SI VERFISEXE=2
SEXE=3 SI VERFISEXE=VIDE

SI VERIFSEXE VIDE, RECODER AVEC SEXE_CIBLE
SEXE=1 SI SEXE_CIBLE =1
SEXE=2 SI SEXE_CIBLE =2
SEXE=3 SI SEXE_CIBLE =VIDE

TOP MODULE A – IDENTIFIER LES PRATIQUANTS

Nous allons aborder les activités physiques et sportives que vous pratiquez, y compris à l'occasion de vos déplacements professionnels et de vos déplacements domicile-travail ou domicile-école.

A TOUS

SIMPLE

A1

Au cours des douze derniers mois, avez-vous fait, même occasionnellement, y compris pendant vos vacances, des activités physiques et sportives ?

- 🕮 ***Si âge< 25*** *Nous parlons des activités physiques et sportives, en dehors des cours obligatoires d'éducation physique et sportive à l'école.*
- 🕮 *Les jeux de société – jeux de cartes – jeux vidéo – activités de jardinage – bricolage – activités manuelles ne sont pas acceptés.*

- ❍ 1. Oui
- ❍ 2. Non

TOP FIN MODULE A

© IPSOS - CONFIDENTIAL

TOP MODULE B – LES PRATIQUES

SI A1 = 1

MULTIPLE

B1

Quelle(s) activité(s) physique(s) ou sportives(s) avez-vous pratiquée(s) dans les douze derniers mois ?

- *Si âge< 25 « en dehors des cours obligatoires d'éducation physique et sportive à l'école » ?*
- *Les jeux de société – jeux de cartes – jeux vidéo – activités de jardinage – bricolage – activités manuelles ne sont pas acceptés.*
- *Ne pas citer - Plusieurs réponses possibles*
- *ENQ : réponse en spontanée*

AFFICHER PAR ORDRE ALPHABETIQUE

1. Accrobranche/parcours acrobatiques en hauteur - 140
2. Aïkido - 154
3. Alpinisme (hors ski alpinisme) - 137
4. Apnée/plongée libre - 64
5. Aquabike - 33
6. Aquagym - 32
7. Aquathlon - 179
8. Athlétisme adapté - 194
9. Aviation/avion - 144
10. Aviron - 76
11. Babyfoot - 112
12. Badminton - 99
13. Badminton sourds - 195
14. Baignade dans les bassins et rivières - 60
15. Baignade en lagon - 61
16. Base jump - 148
17. Basketball - 117
18. Basketball adapté - 197
19. Bateau à moteur - 72
20. Beach rugby - 123
21. Beach tennis et raquette de plage - 101
22. Beach volley - 121
23. Biking/vélo d'appartement - 35
24. Billard - 108
25. BMX - 84
26. Boccia - 198
27. Bodyboard - 66
28. Bowling - 110
29. Bowling sourds - 199
30. Boxe américaine/Full-contact - 159
31. Boxe anglaise - 161

32. Boxe française (savate) - 162
33. Boxe thaï (muay thaï, muay boran...) - 163
34. Bras de fer - 184
35. Cali escrima/Kali eskrima/Kali Arnis Eskrima (KAE) - 185
36. Canne de combat - 186
37. Canoë Kayak - 75
38. Canyoning/randonnée aquatique - 142
39. Capoeira - 164
40. Cardio training - 36
41. Cécifoot - 200
42. Cerf-volant - 145
43. Chasse - 175
44. Chute libre - 188
45. Country - 49
46. Course d'orientation - 13
47. Course de chiens de traîneaux - 191
48. Course de demi-fond - 9
49. Course de fond - 8
50. Course de haies - 18
51. Course de moto sur piste - 91
52. Course de moto sur route - 90
53. Course de sprint - 17
54. Course marathon - 10
55. Course semi-marathon - 11
56. Course sur route - 16
58. Course ultra-endurance/ultrafond/ultratrail/longue distance - 15
59. Courses automobiles - 96
60. Courses ludiques et à obstacles - 14
61. Croche - 187
62. Cross training/Crossfit - 43
63. Culturisme/Bodybuilding - 40
64. Cyclisme adapté - 201
65. Cyclotourisme - 83
66. Danse classique - 45
67. Danse moderne ou contemporaine - 46
68. Danse urbaine/Hip-hop/Breakdance - 48
69. Décathlon - 21
70. Deltaplane - 146
71. Développé couché et musculation adaptée - 183
72. Duathlon - 24
73. Équitation adaptée - 203
74. Equitation : Course équestre - 170
75. Equitation : Cross, cross-country - 168
76. Equitation : Dressage - 169

77. Equitation : Randonnée équestre - 171
78. Equitation : saut d'obstacle - 167
79. Escalade en plein air ("outdoor") - 138
80. Escalade en salle ("indoor") - 139
81. Escrime - 158
82. Escrime handisport - 202
83. Fitness/aérobic/step/ Body balance etc… - 34
84. Fléchettes - 109
85. Foot de plage/beach soccer - 115
86. Foot fauteuil électrique - 116
87. Foot mal-marchants - 204
88. Football (five, foot de rue, futsal, club) - 114
89. Football sourds - 205
90. Goalball - 206
91. Golf - 105
92. Gymnastique acrobatique/cheerleading - 181
93. Gymnastique douce - 27
94. Gymnastique rythmique - 29
95. Gymnastique sportive - 28
96. Gyropode - 192
97. Haltérophilie - 41
98. Handball - 118
99. Handball sourds - 207
100. Heptathlon - 22
101. Hydrospeed/nage en eaux vives - 59
102. Jet ski - 73
103. Jiu-jitsu brésilien - 155
104. Jogging/footing/course à pied/running - 7
105. Judo - 152
106. Judo adapté - 208
107. Karaté - 153
108. Karting - 94
109. Kayak - 78
110. Kayak de mer - 80
111. Kayak-polo - 79
112. Kickboxing - 160
113. Kite surf - 70
114. Kung fu - 156
115. Lancers (poids, disque, marteau, javelot) - 20
116. Lasergame - 113
117. Les échecs - 176
118. Longe côte/marche aquatique côtière (marche dans l'eau) - 6
119. Luge - 132
120. Lutte - 165

121. Marche athlétique - 2
122. Marche nordique - 1
123. Marche/balade - 3
124. MMA (arts martiaux mixtes) - 166
125. Moringue - 189
126. Moto - 89
127. Moto tout terrain/Moto-cross - 92
128. Motonautisme - 74
129. Motoneige - 136
130. Musculation - 39
131. Nage avec palmes (en piscine) - 58
132. Nage/Natation (en piscine) - 57
133. Natation adaptée - 209
134. Padel - 102
135. Paintball/Airsoft - 111
136. Parachutisme - 147
137. Parapente - 149
138. Parkour/free run - 44
139. Patinage artistique - 134
140. Patinage de vitesse - 135
141. Pêche - 172
142. Pêche sous-marine - 173
143. Pêche sportive - 174
144. Pelote basque - 125
145. Pétanque - 103
146. Pilates - 26
147. Planche à voile/Windsurf - 69
148. Plongée sous-marine - 62
149. Plongée subaquatique adaptée - 211
150. Pole Danse - 51
151. Qi gong/qigong/chi gong/chi kung/Gym chinoise - 55
152. Quad - 95
153. Raid multisports/d'endurance - 5
154. Rameur d'intérieur - 37
155. Randonnée adaptée - 212
156. Randonnée pédestre (petite ou grande randonnée, trekking…) - 4
157. Raquette à neige - 133
158. Relaxation - 54
159. Roller - 86
160. Rugby (7, 13, 15) - 122
161. Rugby Fauteuil - 213
162. Run and bike - 25
163. Salsa - 50
164. Sandball/Beach handball - 119

© IPSOS - CONFIDENTIAL

165. Sarbacane - 190
166. Sauts (hauteur, longueur, perche…) - 19
167. Showdown - 214
168. Simulateur d'activités physiques ou sportives (Wii…) - 177
169. Skateboard - 88
170. Ski alpin et snowboard adapté - 215
171. Ski alpin/freeride - 127
172. Ski artistique/acrobatique/freestyle - 128
173. Ski de fond/ski nordique - 129
174. Ski de randonnée/ski alpinisme - 130
175. Ski nautique/nu-pieds/aquaplane - 67
176. Ski nordique adapté - 216
177. Slackline - 52
178. Snorkeling/nage avec palmes (palmes, masque, tuba) - 63
179. Spéléologie tunnel - 143
180. Sports de boules/pétanque adaptée - 210
181. Sports de boules (hors pétanque) - 104
182. Sports de pagaie : pirogue, dragon boat - 81
183. Squash - 98
184. Stand up paddle (SUP) - 77
185. Street workout/musculation urbaine - 38
186. Stretching - 42
187. Surf - 65
188. Surf de neige/snowboard - 131
189. Swimrun - 180
190. Taekwondo - 157
191. Taïchi/Tai chi/Taïchi chuan/taiji quan/Gym chinoise - 56
192. Tennis - 97
193. Tennis adapté - 217
194. Tennis de table (ping-pong) - 100
195. Tennis de table adapté - 218
196. Tir à l'arc - 107
197. Tir à l'arc adapté - 219
198. Tir sportif adapté - 196
199. Tir sportif/ball trap - 106
200. Torball - 220
201. Trail/course de montagne - 12
202. Trampoline - 30
203. Triathlon - 23
204. Trottinette - 87
205. Tumbling - 182
206. Twirling bâton - 31
207. ULM - 150
208. Ultimate/Frisbee - 126

© IPSOS - CONFIDENTIAL

209. Vélo électrique - 193
210. Vélo free style (Trial, Dirt…) - 93
211. Vélo sur route/cyclisme - 82
212. Via ferrata - 141
213. Voile - 71
214. Voile adaptée - 221
215. Vol à voile - 151
216. Volley-ball - 120
217. Volley-ball sourds - 222
218. VTT sur piste et chemin - 85
219. Wakeboard - 68
220. Water-polo - 124
221. Yoga - 53
222. Zumba - 47
223. Autres activités de la force : Champs ouvert - 241
224. Autres activités de la forme et du bien-être : Champs ouvert - 227
225. Autres activités physiques ou sportives : champs ouvert - 247
226. Autres activités verticales et d'équilibre, à corde, en hauteur : Champs ouvert - 237
227. Autres arts martiaux et sports de combat : Champs ouvert - 245
228. Autres danses : Champs ouvert - 228
229. Autres sports adaptés aux situations de handicap : Champs ouvert - 240
230. Autres sports aériens : Champs ouvert - 238
231. Autres sports aquatiques et nautiques : Champs ouvert - 225
232. Autres sports collectifs ou de plage : Champs ouvert - 235
233. Autres sports combinés et enchainés : Champs ouvert - 246
234. Autres sports d'athlétisme : Champs ouvert - 243
235. Autres sports d'eaux vives : Champs ouvert - 230
236. Autres sports d'équitation : Champs ouvert - 239
237. Autres sports d'hiver ou de montagne : Champs ouvert - 236
238. Autres sports de course : Champs ouvert - 224
239. Autres sports de glisse : Champs ouvert - 229
240. Autres sports de gymnastique : Champs ouvert - 226
241. Autres sports de marche : Champs ouvert - 223
242. Autres sports de moto : Champs ouvert - 242
243. Autres sports de précision ou de cible : Champs ouvert - 234
244. Autres sports de raquette et de balle : Champs ouvert - 233
245. Autres sports de vélo : Champs ouvert - 231
246. Autres sports de VTT : Champs ouvert - 232
247. Autres Sports urbains : Champs ouvert - 244

RECODER B1 SANS POSER

MULTIPLE

B1R

Quelle(s) activité(s) physique(s) ou sportives(s) avez-vous pratiquée(s) dans les douze derniers mois ?

- *Si âge< 25 « en dehors des cours obligatoires d'éducation physique et sportive à l'école » ?*
- *Les jeux de société – jeux de cartes – jeux vidéo – activités de jardinage – bricolage – activités manuelles ne sont pas acceptés.*
- *Ne pas citer - Plusieurs réponses possibles*
- *ENQ : réponse en spontanée*

1. Marche nordique
2. Marche athlétique
3. Marche/balade
4. Randonnée pédestre (petite ou grande randonnée, trekking…)
5. Raid multisports/d'endurance
6. Longe côte/marche aquatique côtière (marche dans l'eau)
7. Jogging/footing/course à pied/running
8. Course de fond
9. Course de demi-fond
10. Course marathon
11. Course semi-marathon
12. Trail/course de montagne
13. Course d'orientation
14. Courses ludiques et à obstacles
15. Course ultra-endurance/ultrafond/ultratrail/longue distance
16. Course sur route
17. Athlétisme : Course de sprint
18. Athlétisme : Course de haies
19. Athlétisme : Sauts (hauteur, longueur, perche…)
20. Athlétisme : Lancers (poids, disque, marteau, javelot)
21. Athlétisme : Décathlon
22. Athlétisme : Heptathlon
23. Athlétisme : Triathlon
24. Athlétisme : Duathlon
25. Athlétisme : Run and bike
26. Pilates
27. Gymnastique douce
28. Gymnastique sportive
29. Gymnastique rythmique
30. Trampoline
31. Twirling bâton
32. Aquagym
33. Aquabike
34. Fitness/aérobic/step/ Body balance etc…
35. Biking/vélo d'appartement
36. Cardio training
37. Rameur d'intérieur
38. Street workout/musculation urbaine
39. Musculation
40. Culturisme/Bodybuilding
41. Haltérophilie

42. Stretching
43. Cross training/Crossfit
44. Parkour/free run
45. Danse classique
46. Danse moderne ou contemporaine
47. Zumba
48. Danse urbaine/Hip-hop/Breakdance
49. Country
50. Salsa
51. Pole Danse
52. Slackline
53. Yoga
54. Relaxation
55. Qi gong/qigong/chi gong/chi kung/Gym chinoise
56. Taïchi/Tai chi/Taïchi chuan/taiji quan/Gym chinoise
57. Nage/Natation (en piscine)
58. Nage avec palmes (en piscine)
59. Hydrospeed/nage en eaux vives
60. Baignade dans les bassins et rivières
61. Baignade en lagon
62. Plongée sous-marine
63. Snorkeling/nage avec palmes (palmes, masque, tuba)
64. Apnée/plongée libre
65. Surf
66. Bodyboard
67. Ski nautique/nu-pieds/aquaplane
68. Wakeboard
69. Planche à voile/Windsurf
70. Kite surf
71. Voile
72. Bateau à moteur
73. Jet ski
74. Motonautisme
75. Canoë Kayak
76. Aviron
77. Stand up paddle (SUP)
78. Kayak
79. Kayak-polo
80. Kayak de mer
81. Sports de pagaie : pirogue, dragon boat
82. Vélo sur route/cyclisme
83. Cyclotourisme
84. BMX
85. VTT sur piste et chemin
86. Roller
87. Trottinette
88. Skateboard
89. Moto
90. Course de moto sur route

© IPSOS - CONFIDENTIAL

91. Course de moto sur piste
92. Moto tout terrain/Moto-cross
93. Vélo free style (Trial, Dirt…)
94. Karting
95. Quad
96. Courses automobiles
97. Tennis
98. Squash
99. Badminton
100. Tennis de table (ping-pong)
101. Beach tennis et raquette de plage
102. Padel
103. Pétanque
104. Sports de boules (hors pétanque)
105. Golf
106. Tir sportif/ball trap
107. Tir à l'arc
108. Billard
109. Fléchettes
110. Bowling
111. Paintball/Airsoft
112. Babyfoot
113. Lasergame
114. Football (five, foot de rue, futsal, club)
115. Foot de plage/beach soccer
116. Foot fauteuil électrique
117. Basketball
118. Handball
119. Sandball/Beach handball
120. Volley-ball
121. Beach volley
122. Rugby (7, 13, 15)
123. Beach rugby
124. Water-polo
125. Pelote basque
126. Ultimate/Frisbee
127. Ski alpin/freeride
128. Ski artistique/acrobatique/freestyle
129. Ski de fond/ski nordique
130. Ski de randonnée/ski alpinisme
131. Surf de neige/snowboard
132. Luge
133. Raquette à neige
134. Patinage artistique
135. Patinage de vitesse
136. Motoneige
137. Alpinisme (hors ski alpinisme)
138. Escalade en plein air ("outdoor")
139. Escalade en salle ("indoor")

© IPSOS - CONFIDENTIAL

140. Accrobranche/parcours acrobatiques en hauteur
141. Via ferrata
142. Canyoning/randonnée aquatique
143. Spéléologie tunnel
144. Aviation/avion
145. Cerf-volant
146. Deltaplane
147. Parachutisme
148. Base jump
149. Parapente
150. ULM
151. Vol à voile
152. Judo
153. Karaté
154. Aïkido
155. Jiu-jitsu brésilien
156. Kung fu
157. Taekwondo
158. Escrime
159. Boxe américaine/Full-contact
160. Kickboxing
161. Boxe anglaise
162. Boxe française (savate)
163. Boxe thaï (muay thaï, muay boran…)
164. Capoeira
165. Lutte
166. MMA (arts martiaux mixtes)
167. Equitation : saut d'obstacle
168. Equitation : Cross, cross-country
169. Equitation : Dressage
170. Equitation : Course équestre
171. Equitation : Randonnée équestre
172. Pêche
173. Pêche sous-marine
174. Pêche sportive
175. Chasse
176. Les échecs
177. Simulateur d'activités physiques ou sportives (Wii…)
179. Aquathlon
180. Swimrun
181. Gymnastique acrobatique/cheerleading
182. Tumbling
183. Développé couché et musculation adaptée
184. Bras de fer
185. Cali escrima/Kali eskrima/Kali Arnis Eskrima (KAE)
186. Canne de combat
187. Croche
188. Chute libre
189. Moringue

© IPSOS - CONFIDENTIAL

190. Sarbacane
191. Course de chiens de traîneaux
192. Gyropode
193. Vélo électrique
194. Athlétisme adapté
195. Badminton sourds
196. Tir sportif adapté
197. Basketball adapté
198. Boccia
199. Bowling sourds
200. Cécifoot
201. Cyclisme adapté
202. Escrime handisport
203. Équitation adaptée
204. Foot mal-marchants
205. Football sourds
206. Goalball
207. Handball sourds
208. Judo adapté
209. Natation adaptée
210. Sports de boules/pétanque adaptée
211. Plongée subaquatique adaptée
212. Randonnée adaptée
213. Rugby Fauteuil
214. Showdown
215. Ski alpin et snowboard adapté
216. Ski nordique adapté
217. Tennis adapté
218. Tennis de table adapté
219. Tir à l'arc adapté
220. Torball
221. Voile adaptée
222. Volley-ball sourds
223. Autres sports de marche : **Champs ouvert**
224. Autres sports de course : **Champs ouvert**
225. Autres sports aquatiques et nautiques : **Champs ouvert**
226. Autres sports de gymnastique : **Champs ouvert**
227. Autres activités de la forme et du bien-être : **Champs ouvert**
228. Autres danses : **Champs ouvert**
229. Autres sports de glisse : **Champs ouvert**
230. Autres sports d'eaux vives : **Champs ouvert**
231. Autres sports de vélo : **Champs ouvert**
232. Autres sports de VTT : **Champs ouvert**
233. Autres sports de raquette et de balle : **Champs ouvert**
234. Autres sports de précision ou de cible : **Champs ouvert**
235. Autres sports collectifs ou de plage : **Champs ouvert**
236. Autres sports d'hiver ou de montagne : **Champs ouvert**
237. Autres activités verticales et d'équilibre, à corde, en hauteur : **Champs ouvert**
238. Autres sports aériens : **Champs ouvert**

© IPSOS - CONFIDENTIAL

239. Autres sports d'équitation : **Champs ouvert**
240. Autres sports adaptés aux situations de handicap : **Champs ouvert**
241. Autres activités de la force : **Champs ouvert**
242. Autres sports de moto : **Champs ouvert**
243. Autres sports d'athlétisme : **Champs ouvert**
244. Autres Sports urbains : **Champs ouvert**
245. Autres arts martiaux et sports de combat : **Champs ouvert**
246. Autres sports combinés et enchainés : **Champs ouvert**
247. Autres activités physiques ou sportives : **Champs ouvert**

TOP LISTE PRATIQUES

A TOUS

MULTIPLE

B2

Afin d'être sûr que vous n'auriez pas oublié une activité physique ou sportive, je vais vous donner une liste d'activités. Vous m'arrêterez en disant « Oui » quand vous entendrez une activité que vous avez pratiquée au cours des douze derniers mois.

PROG : NE PAS AFFICHER LES ITEMS EN B2 SI DEJA COCHE EN B1

- ***Si âge < 25*** *« en dehors des cours obligatoires d'éducation physique et sportive à l'école ».*
- *ENQ : citer - Plusieurs réponses possibles*

1. Marche
2. Randonnée pédestre (petite ou grande randonnée, trekking) → **NE PAS AFFICHER SI B1R =4**
3. Running ou autre sport de course
4. ~~Athlétisme~~
5. Danse
6. Gymnastique aquatique
7. Gymnastique, pilates
8. ~~Stretching → NE PAS AFFICHER SI B1R =42~~
9. Yoga ou autre gymnastique asiatique
10. Relaxation → **NE PAS AFFICHER SI B1R =54**
11. Musculation ou autre sport de force
12. Fitness ou autre sport de forme
13. Baignade (en lagon ou rivière)
14. ~~Nage/Natation (en piscine) → NE PAS AFFICHER SI B1R =57~~
15. Voile → **NE PAS AFFICHER SI B1R =71**
16. Bateau à moteur → **NE PAS AFFICHER SI B1R =72**
17. ~~Jet ski → NE PAS AFFICHER SI B1R =73~~
18. ~~Planche à voile/Windsurf → NE PAS AFFICHER SI B1R =69~~
19. Surf ou bodyboard
20. Plongée ou snorkeling
21. ~~Canoë-kayak ou autre sport de pagaie~~
22. Autre activité aquatique ou nautique → **NE PAS AFFICHER SI B1R =225**
23. Vélo
24. VTT
25. Roller → **NE PAS AFFICHER SI B1R =86**
26. Skateboard → **NE PAS AFFICHER SI B1R =88**

27. Trottinette➔ **NE PAS AFFICHER SI B1R =87**
~~28. Patinage~~
29. Moto ou autre sport de moto
~~30. Courses automobiles➔ NE PAS AFFICHER SI B1R =96~~
31. Karting➔ **NE PAS AFFICHER SI B1R =94**
~~32. Quad➔ NE PAS AFFICHER SI B1R =95~~
33. Jeu de boules, jeu de pétanque
34. Tir, tir à l'arc ou autre sport de précision
~~35. Golf➔ NE PAS AFFICHER SI B1R =105~~
36. Tennis (sur court ou à la plage)
37. ~~Squash➔ NE PAS AFFICHER SI B1R =98~~
38. Tennis de table (ping-pong) ➔ **NE PAS AFFICHER SI B1R =100**
39. Badminton➔ **NE PAS AFFICHER SI B1R =99**
40. Autre jeu de raquettes
41. Football (sur terrain ou à la plage)
42. Basketball➔ **NE PAS AFFICHER SI B1R =117**
~~43. Handball (sur terrain ou à la plage)~~
44. Volley-ball (sur terrain ou à la plage)
45. Rugby (sur terrain ou à la plage)
46. Autre sport collectif
47. Ski
~~48. Luge➔ NE PAS AFFICHER SI B1R =132~~
49. Surf de neige/snowboard➔ **NE PAS AFFICHER SI B1R =131**
~~50. Motoneige➔ NE PAS AFFICHER SI B1R =136~~
51. Autre sport d'hiver
~~52. Alpinisme (hors ski alpinisme) ➔ NE PAS AFFICHER SI B1R =137~~
53. Escalade
54. Accrobranche, via ferrata ou autre parcours en hauteur
55. Autre activité verticales et d'équilibre, à corde, en hauteur : **Champs ouvert➔ NE PAS AFFICHER SI B1R =237**
56. Parapente➔ **NE PAS AFFICHER SI B1R =149**
~~57. Deltaplane➔ NE PAS AFFICHER SI B1R =146~~
58. Parachutisme➔ **NE PAS AFFICHER SI B1R =147**
~~59. Aviation/avion➔ NE PAS AFFICHER SI B1R =144~~
~~60. ULM➔ NE PAS AFFICHER SI B1R =150~~
61. Autre sport aérien➔ **NE PAS AFFICHER SI B1R =238**
62. Judo➔ **NE PAS AFFICHER SI B1R =152**
~~63. Karaté➔ NE PAS AFFICHER SI B1R =153~~
64. Autre sport de combat ou art martial➔ **NE PAS AFFICHER SI B1R =245**
65. Equitation
66. Pêche ou autre type de pêche
67. ~~Chasse➔ NE PAS AFFICHER SI B1R =175~~
68. Les échecs➔ **NE PAS AFFICHER SI B1R =176**
69. Simulateur d'activités physiques ou sportives (Wii…)➔ **NE PAS AFFICHER SI B1R =177**
70. Cerf-volant➔ **NE PAS AFFICHER SI B1R =145**
71. Autres sports de glisse : **Champs ouvert➔ NE PAS AFFICHER SI B1R =229**
72. Aucune activité : **item exclusif**

SI AUCUNE ACTIVITE (CODE 72) EN B2, PASSER A LA QUESTION D1

TOP

SI B2=1 ET (B1R<>1 ET 2 ET 3 ET 223)

MULTIPLE

B2_1

Vous m'avez dit pratiquer de la marche. S'agit-il de...?

📖 *ENQ : citer – plusieurs réponses possibles*

- Marche/balade ➔ **ne pas afficher si B1R=3**
- Marche nordique ➔ **ne pas afficher si B1R=1**
- Marche athlétique ➔ **ne pas afficher si B1R=2**
- Autres sports de marche : **Champ ouvert** ➔ **ne pas afficher si B1R=223**
- (Aucune autre activité que celles citées précédemment) **EXCLUSIF**

SI B2=3 ET (B1R<>7 ET 12 ET 13 ET 14 ET 10 ET 11 ET 16 ET 8 ET 9 ET 15 ET 224)

MULTIPLE

B2_2

Vous m'avez dit pratiquer du Running ou autre sport de course. S'agit-il de...?

📖 *ENQ : citer – plusieurs réponses possibles*

- Jogging/footing/course à pied/running ➔ **ne pas afficher si B1R=7**
- Trail/course de montagne ➔ **ne pas afficher si B1R=12**
- Course d'orientation ➔ **ne pas afficher si B1R=13**
- Courses ludiques et à obstacles ➔ **ne pas afficher si B1R=14**
- Course marathon ➔ **ne pas afficher si B1R=10**
- Course semi-marathon ➔ **ne pas afficher si B1R=11**
- Course sur route ➔ **ne pas afficher si B1R=16**
- Course de fond ➔ **ne pas afficher si B1R=8**
- Course de demi-fond ➔ **ne pas afficher si B1R=9**
- Course ultra-endurance/ultrafond/ultratrail/longue distance ➔ **ne pas afficher si B1R=15**
- Autres sports de course : **Champs ouvert** ➔ **ne pas afficher si B1R=224**
- (Aucune autre activité que celles citées précédemment) **EXCLUSIF**

SI B2=4 ET (B1R<>17 ET 18 ET 19 ET 20 ET 21 ET 22 ET 243)

MULTIPLE

B2_3

Vous m'avez dit pratiquer de l'athlétisme. S'agit-il de...?

📖 *ENQ : citer – plusieurs réponses possibles*

- Course de sprint ➔ **ne pas afficher si B1R=17**
- Course de haies ➔ **ne pas afficher si B1R=18**
- Sauts (hauteur, longueur, perche...) ➔ **ne pas afficher si B1R=19**
- Lancers (poids, disque, marteau, javelot) ➔ **ne pas afficher si B1R=20**
- Décathlon ➔ **ne pas afficher si B1R=21**
- Heptathlon ➔ **ne pas afficher si B1R=22**

- Autres sports d'athlétisme : **Champs ouvert** → **ne pas afficher si B1R=243**
- (Aucune autre activité que celles citées précédemment) **EXCLUSIF**

SI B2=5 ET (B1R<>45 ET 46 ET 48 ET 50 ET 49 ET 51 ET 181 ET 29 ET 28 ET 31 ET 182 ET 228)

MULTIPLE

B2_4

Vous m'avez dit pratiquer de la danse. S'agit-il de...?

ENQ : citer– plusieurs réponses possibles

- Danse classique → **ne pas afficher si B1R=45**
- Danse moderne ou contemporaine → **ne pas afficher si B1R=46**
- Danse urbaine/Hip-hop/Breakdance → **ne pas afficher si B1R=48**
- Salsa → **ne pas afficher si B1R=50**
- Country → **ne pas afficher si B1R=49**
- Pole Danse → **ne pas afficher si B1R=51**
- Gymnastique acrobatique/cheerleading → **ne pas afficher si B1R=181 ou B2=7**
- Gymnastique rythmique → **ne pas afficher si B1R=29 ou B2=7**
- Gymnastique sportive → **ne pas afficher si B1R=28 ou B2=7**
- Twirling bâton → **ne pas afficher si B1R=31 ou B2=7**
- Tumbling → **ne pas afficher si B1R=182 ou B2=7**
- Autres danses : **Champs ouvert** → **ne pas afficher si B1R=228**
- (Aucune autre activité que celles citées précédemment) **EXCLUSIF**

SI B2=6 ET (B1R<>32 ET 33)

MULTIPLE

B2_5

Vous m'avez dit pratiquer de la gymnastique aquatique. S'agit-il de...?

ENQ : citer– plusieurs réponses possibles

- Aquagym → **ne pas afficher si B1R=32**
- Aquabike → **ne pas afficher si B1R=33**
- (Aucune autre activité que celles citées précédemment) **EXCLUSIF**

SI B2=7 ET (B1R<>26 ET 27 ET 181 ET 29 ET 28 ET 31 ET 182 ET 226)

MULTIPLE

B2_6

Vous m'avez dit pratiquer de la gymnastique, pilates. S'agit-il de...?

ENQ : citer– plusieurs réponses possibles

- Pilates → **ne pas afficher si B1R=26**
- Gymnastique douce → **ne pas afficher si B1R=27**
- Gymnastique acrobatique/cheerleading → **ne pas afficher si B1R=181**
- Gymnastique rythmique → **ne pas afficher si B1R=29**
- Gymnastique sportive → **ne pas afficher si B1R=28**
- Twirling bâton → **ne pas afficher si B1R=31**
- Tumbling → **ne pas afficher si B1R=182**
- Autres sports de gymnastique : **Champs ouvert** → **ne pas afficher si B1R=226**

- (Aucune autre activité que celles citées précédemment) **EXCLUSIF**

SI B2=9 ET (B1R<>53 ET 55 ET 56)

MULTIPLE

B2_7

Vous m'avez dit pratiquer du yoga ou autre gymnastique asiatique. S'agit-il de...?

ENQ : citer– plusieurs réponses possibles

- Yoga→ **ne pas afficher si B1R=53**
- Chi gong/Gym chinoise→ **ne pas afficher si B1R=55**
- Taïchi chuan/Gym chinoise→ **ne pas afficher si B1R=56**
- (Aucune autre activité que celles citées précédemment) **EXCLUSIF**

SI B2=11 ET (B1R<>39 ET 40 ET 42 ET 38 ET 241)

MULTIPLE

B2_8

Vous m'avez dit pratiquer de la musculation ou autre sport de force. S'agit-il de...?

ENQ : citer– plusieurs réponses possibles

- Musculation→ **ne pas afficher si B1R=39**
- Culturisme/Bodybuilding→ **ne pas afficher si B1R=40**
- Haltérophilie→ **ne pas afficher si B1R=42**
- Street workout/musculation urbaine→ **ne pas afficher si B1R=38**
- Autres activités de la force : **Champs ouvert→ ne pas afficher si B1R=241**
- (Aucune autre activité que celles citées précédemment) **EXCLUSIF**

SI B2=12 ET (B1R<>34 ET 35 ET 36 ET 37 ET 43 ET 227)

MULTIPLE

B2_9

Vous m'avez dit pratiquer du fitness ou autre sport de forme. S'agit-il de...?

ENQ : citer– plusieurs réponses possibles

- Fitness/aérobic/step/ Body balance etc...→ **ne pas afficher si B1R=34**
- Biking/vélo d'appartement→ **ne pas afficher si B1R=35**
- Cardio training→ **ne pas afficher si B1R=36**
- Rameur d'intérieur→ **ne pas afficher si B1R=37**
- Cross training/Crossfit→ **ne pas afficher si B1R=43**
- Autres activités de la forme et du bien-être : **Champs ouvert→ ne pas afficher si B1R=227**
- (Aucune autre activité que celles citées précédemment) **EXCLUSIF**

SI B2=13 ET (B1R<>60 ET 61)

MULTIPLE

B2_10

Vous m'avez dit pratiquer de la baignade (en lagon ou rivière). S'agit-il de...?

ENQ : citer– plusieurs réponses possibles

- Baignade dans les bassins et rivières→ **ne pas afficher si B1R=60**

- Baignade en lagon→ **ne pas afficher si B1R=61**
- (Aucune autre activité que celles citées précédemment) **EXCLUSIF**

SI B2=19 ET (B1R<>65 ET 66)

MULTIPLE

B2_11

Vous m'avez dit pratiquer du Surf ou bodyboard. S'agit-il de...?

ENQ : citer– plusieurs réponses possibles

- Surf→ **ne pas afficher si B1R=65**
- Bodyboard→ **ne pas afficher si B1R=66**
- (Aucune autre activité que celles citées précédemment) **EXCLUSIF**

SI B2=20 ET (B1R<>62 ET 63 ET 64)

MULTIPLE

B2_12

Vous m'avez dit pratiquer de la plongée. S'agit-il de...?

ENQ : citer– plusieurs réponses possibles

- Plongée sous-marine→ **ne pas afficher si B1R=62**
- Snorkeling/nage avec palmes (palmes, masque, tuba) → **ne pas afficher si B1R=63**
- Apnée/plongée libre→ **ne pas afficher si B1R=64**
- (Aucune autre activité que celles citées précédemment) **EXCLUSIF**

SI B2=21 ET (B1R<>75 ET 80 ET 76 ET 81)

MULTIPLE

B2_13

Vous m'avez dit pratiquer du canoë-kayak ou autre sport de pagaie. S'agit-il de...?

ENQ : citer– plusieurs réponses possibles

- Canoë Kayak→ **ne pas afficher si B1R=75**
- Kayak de mer→ **ne pas afficher si B1R=80**
- Aviron→ **ne pas afficher si B1R=76**
- Sports de pagaie : pirogue, dragon boat→ **ne pas afficher si B1R=81**
- (Aucune autre activité que celles citées précédemment) **EXCLUSIF**

SI B2=22 ET (B1R<>6 ET 70 ET 67 ET 68 ET 77 ET 225)

MULTIPLE

B2_14

Vous m'avez dit pratiquer une autre activité aquatique ou nautique. S'agit-il de...?

ENQ : citer– plusieurs réponses possibles

- Longe côte/marche aquatique côtière (marche dans l'eau) → **ne pas afficher si B1R=6**
- Kitesurf→ **ne pas afficher si B1R=70**
- Ski nautique/nu-pieds/aquaplane→ **ne pas afficher si B1R=67**
- Wakeboard→ **ne pas afficher si B1R=68**
- Stand up paddle (SUP) → **ne pas afficher si B1R=77**
- Autres sports aquatiques et nautiques : **Champs ouvert**→ **ne pas afficher si B1R=225**

© IPSOS - CONFIDENTIAL

- (Aucune autre activité que celles citées précédemment) **EXCLUSIF**

SI B2=23 ET (B1R<>82 ET 83 ET 193 ET 231)

MULTIPLE

B2_15

Vous m'avez dit pratiquer du vélo. S'agit-il de...?

ENQ : citer– plusieurs réponses possibles

- Vélo sur route/cyclisme → **ne pas afficher si B1R=82**
- Cyclotourisme → **ne pas afficher si B1R=83**
- Vélo électrique → **ne pas afficher si B1R=193**
- Autres sports de vélo : **Champs ouvert** → **ne pas afficher si B1R=231**
- (Aucune autre activité que celles citées précédemment) **EXCLUSIF**

SI B2=24 ET (B1R<>85 ET 84 ET 232)

MULTIPLE

B2_16

Vous m'avez dit pratiquer du VTT. S'agit-il de...?

ENQ : citer– plusieurs réponses possibles

- VTT sur piste et chemin → **ne pas afficher si B1R=85**
- BMX → **ne pas afficher si B1R=84**
- Autres sports de VTT : **Champs ouvert** → **ne pas afficher si B1R=232**
- (Aucune autre activité que celles citées précédemment) **EXCLUSIF**

SI B2=28 ET (B1R<>134 ET 135)

MULTIPLE

B2_17

Vous m'avez dit pratiquer du patinage. S'agit-il de...?

ENQ : citer– plusieurs réponses possibles

- Patinage artistique → **ne pas afficher si B1R=134**
- Patinage de vitesse → **ne pas afficher si B1R=135**
- (Aucune autre activité que celles citées précédemment) **EXCLUSIF**

SI B2=29 ET (B1R<>89 ET 90 ET 91 ET 92 ET 242)

MULTIPLE

B2_18

Vous m'avez dit pratiquer de la Moto ou autre sport de moto. S'agit-il de...?

ENQ : citer– plusieurs réponses possibles

- Moto → **ne pas afficher si B1R=89**
- Course de moto sur route → **ne pas afficher si B1R=90**
- Course de moto sur piste → **ne pas afficher si B1R=91**
- Moto tout terrain/Moto-cross → **ne pas afficher si B1R=92**
- Autres sports de moto : **Champs ouvert** → **ne pas afficher si B1R=242**
- (Aucune autre activité que celles citées précédemment) **EXCLUSIF**

SI B2=33 ET (B1R<>103 ET 104)

MULTIPLE

B2_19

Vous m'avez dit pratiquer des jeux de boules et de pétanque. S'agit-il de...?

ENQ : citer – plusieurs réponses possibles

- Pétanque → **ne pas afficher si B1R=103**
- Sports de boules (hors pétanque) → **ne pas afficher si B1R=104**
- (Aucune autre activité que celles citées précédemment) **EXCLUSIF**

SI B2=34 ET (B1R<>106 ET 107 ET 111 ET 113 ET 109 ET 110 ET 108 ET 234)

MULTIPLE

B2_20

Vous m'avez dit pratiquer du tir, tir à l'arc ou autre sport de précision. S'agit-il de...?

ENQ : citer – plusieurs réponses possibles

- Tir sportif/ball trap → **ne pas afficher si B1R=106**
- Tir à l'arc → **ne pas afficher si B1R=107**
- Paintball/Airsoft → **ne pas afficher si B1R=111**
- Lasergame → **ne pas afficher si B1R=113**
- Fléchettes → **ne pas afficher si B1R=109**
- Bowling → **ne pas afficher si B1R=110**
- Billard → **ne pas afficher si B1R=108**
- Autres sports de précision ou de cible : **Champs ouvert** → **ne pas afficher si B1R=234**
- (Aucune autre activité que celles citées précédemment) **EXCLUSIF**

SI B2=36 ET (B1R<>97 ET 101)

MULTIPLE

B2_21

Vous m'avez dit pratiquer du tennis (sur court ou à la plage). S'agit-il de...?

ENQ : citer – plusieurs réponses possibles

- Tennis → **ne pas afficher si B1R=97**
- Beach tennis et raquette de plage → **ne pas afficher si B1R=101**
- (Aucune autre activité que celles citées précédemment) **EXCLUSIF**

SI B2=40 ET (B1R<>102 ET 125 ET 233)

MULTIPLE

B2_22

Vous m'avez dit pratiquer un autre jeu de raquettes. S'agit-il de...?

ENQ : citer – plusieurs réponses possibles

- Padel → **ne pas afficher si B1R=102**
- Pelote basque → **ne pas afficher si B1R=125**
- Autres sports de raquette et de balle : **Champs ouvert** → **ne pas afficher si B1R=233**
- (Aucune autre activité que celles citées précédemment) **EXCLUSIF**

SI B2=41 ET (B1R<>114 ET 115)

MULTIPLE

B2_23

Vous m'avez dit pratiquer du football (sur terrain ou à la plage). S'agit-il de...?

ENQ : citer– plusieurs réponses possibles

- Football (five, foot de rue, futsal, club) ➔ **ne pas afficher si B1R=114**
- Foot de plage/beach soccer ➔ **ne pas afficher si B1R=115**
- (Aucune autre activité que celles citées précédemment) **EXCLUSIF**

SI B2=43 ET (B1R<>118 ET 119)

MULTIPLE

B2_24

Vous m'avez dit pratiquer du handball (sur terrain ou à la plage). S'agit-il de...?

ENQ : citer– plusieurs réponses possibles

- Handball ➔ **ne pas afficher si B1R=118**
- Sandball/Beach handball ➔ **ne pas afficher si B1R=119**
- (Aucune autre activité que celles citées précédemment) **EXCLUSIF**

SI B2=44 ET (B1R<>120 ET 121)

MULTIPLE

B2_25

Vous m'avez dit pratiquer du volley-ball (sur terrain ou à la plage). S'agit-il de...?

ENQ : citer– plusieurs réponses possibles

- Volley ball ➔ **ne pas afficher si B1R=120**
- Beach volley ➔ **ne pas afficher si B1R=121**
- (Aucune autre activité que celles citées précédemment) **EXCLUSIF**

SI B2=45 ET (B1R<>122 ET 123)

MULTIPLE

B2_26

Vous m'avez dit pratiquer du rugby (sur terrain ou à la plage). S'agit-il de...?

ENQ : citer– plusieurs réponses possibles

- Rugby (7, 13, 15) ➔ **ne pas afficher si B1R=122**
- Beach rugby ➔ **ne pas afficher si B1R=123**
- (Aucune autre activité que celles citées précédemment) **EXCLUSIF**

SI B2=46 ET (B1R<>124 ET 235)

MULTIPLE

B2_27

Vous m'avez dit pratiquer un autre sport collectif. S'agit-il de...?

ENQ : citer– plusieurs réponses possibles

© IPSOS - CONFIDENTIAL

- Water-polo→ **ne pas afficher si B1R=124**
- Autres sports collectifs ou de plage : **Champs ouvert**→ **ne pas afficher si B1R=235**
- (Aucune autre activité que celles citées précédemment) **EXCLUSIF**

SI B2=47 ET (B1R<>127 ET 128 ET 129 ET 130)

MULTIPLE

B2_28

Vous m'avez dit pratiquer du ski. S'agit-il de...?

ENQ : citer– plusieurs réponses possibles

- Ski alpin/freeride→ **ne pas afficher si B1R=127**
- Ski artistique/acrobatique/freestyle→ **ne pas afficher si B1R=128**
- Ski de fond/ski nordique→ **ne pas afficher si B1R=129**
- Ski de randonnée/ski alpinisme→ **ne pas afficher si B1R=130**
- (Aucune autre activité que celles citées précédemment) **EXCLUSIF**

SI B2=51 ET (B1R<>133 ET 191 ET 236)

MULTIPLE

B2_29

Vous m'avez dit pratiquer un autre sport d'hiver. S'agit-il de...?

ENQ : citer– plusieurs réponses possibles

- Raquette à neige→ **ne pas afficher si B1R=133**
- Course de chiens de traîneaux→ **ne pas afficher si B1R=191**
- Autres sports d'hiver ou de montagne : Champs ouvert→ **ne pas afficher si B1R=236**
- (Aucune autre activité que celles citées précédemment) **EXCLUSIF**

SI B2=53 ET (B1R<>138 ET 139)

MULTIPLE

B2_30

Vous m'avez dit pratiquer de l'escalade. S'agit-il de...?

ENQ : citer– plusieurs réponses possibles

- Escalade en plein air ("outdoor")→ **ne pas afficher si B1R=138**
- Escalade en salle ("indoor")→ **ne pas afficher si B1R=139**
- (Aucune autre activité que celles citées précédemment) **EXCLUSIF**

SI B2=54 ET (B1R<>140 ET 141)

MULTIPLE

B2_31

Vous m'avez dit pratiquer de l'accrobranche, via ferrata ou autre parcours en hauteur. S'agit-il de...?

ENQ : citer– plusieurs réponses possibles

- Accrobranche/parcours acrobatiques en hauteur→ **ne pas afficher si B1R=140**
- Via ferrata→ **ne pas afficher si B1R=141**
- (Aucune autre activité que celles citées précédemment) **EXCLUSIF**

© IPSOS - CONFIDENTIAL

SI B2=61 ET (B1R<>188 ET 148 ET 151 ET 238)

MULTIPLE

B2_32

Vous m'avez dit pratiquer un autre sport aérien. S'agit-il de...?

ENQ : citer– plusieurs réponses possibles

- Chute libre→ **ne pas afficher si B1R=188**
- Base jump→ **ne pas afficher si B1R=148**
- Vol à voile→ **ne pas afficher si B1R=151**
- Autres sports aériens : **Champs ouvert**→ **ne pas afficher si B1R=238**
- (Aucune autre activité que celles citées précédemment) **EXCLUSIF**

SI B2=64 ET (B1R<>189 ET 187 ET 165 ET 155 ET 157 ET 166 ET 161 ET 162 ET 163 ET 184 ET 158 ET 245)

MULTIPLE

B2_33

Vous m'avez dit pratiquer un autre sport de combat ou art martial. S'agit-il de...?

ENQ : citer– plusieurs réponses possibles

- Moringue→ **ne pas afficher si B1R=189**
- Croche→ **ne pas afficher si B1R=187**
- Lutte→ **ne pas afficher si B1R=165**
- Jiu-jitsu brésilien→ **ne pas afficher si B1R=155**
- Taekwondo→ **ne pas afficher si B1R=157**
- MMA (arts martiaux mixtes) → **ne pas afficher si B1R=166**
- Boxe anglaise→ **ne pas afficher si B1R=161**
- Boxe française (savate) → **ne pas afficher si B1R=162**
- Boxe thaï (muay thaï, muay boran...)→ **ne pas afficher si B1R=163**
- Bras de fer→ **ne pas afficher si B1R=184**
- Escrime→ **ne pas afficher si B1R=158**
- Autres arts martiaux et sports de combat : **Champs ouvert**→ **ne pas afficher si B1R=245**
- (Aucune autre activité que celles citées précédemment) **EXCLUSIF**

SI B2=65 ET (B1R<>167 ET 168 ET 169 ET 170 ET 171 ET 239)

MULTIPLE

B2_34

Vous m'avez dit pratiquer de l'équitation. S'agit-il de...?

ENQ : citer– plusieurs réponses possibles

- Saut d'obstacle→ **ne pas afficher si B1R=167**
- Cross, cross-country→ **ne pas afficher si B1R=168**
- Dressage→ **ne pas afficher si B1R=169**
- Course équestre→ **ne pas afficher si B1R=170**
- Randonnée équestre→ **ne pas afficher si B1R=171**
- Autres sports d'équitation : Champs ouvert→ **ne pas afficher si B1R=239**
- (Aucune autre activité que celles citées précédemment) **EXCLUSIF**

SI B2=66 ET (B1R<>172 ET 174 ET 173)

MULTIPLE

B2_35

Vous m'avez dit pratiquer de la pêche ou autre type de pêche. Duquel ou desquels s'agit-il ?

ENQ : citer– plusieurs réponses possibles

- Pêche➔ **ne pas afficher si B1R=172**
- Pêche sportive➔ **ne pas afficher si B1R=174**
- Pêche sous-marine➔ **ne pas afficher si B1R=173**
- (Aucune autre activité que celles citées précédemment) **EXCLUSIF**

SI A1 = 2 ET B2= 72 "AUCUNE ACTIVITE AFFICHER « PARLONS MAINTENANT DES SENTIERS ET CHEMINS A PROXIMITE DE VOTRE DOMICILE »

SI A1 = 2 ET B2= 72 "AUCUNE ACTIVITE"

SIMPLE

R3

Connaissez-vous les chemins et sentiers près de votre domicile ?

- 1. Oui
- 2. Non

SI R3 = 1

SIMPLE

R4

Est-ce que ces chemins et sentiers sont facilement accessibles ?

- 1. Oui
- 2. Non

R3 = 2

SIMPLE

R5

Si on vous indiquait ces chemins et sentiers, est-ce que vous les utiliseriez ?

- 1. Oui
- 2. Non

TOP FIN LISTE PRATIQUANTS

PROG : TOUTES LES ACTIVITES PRATIQUEES SERONT RECODEES SUR UNE LISTE GLOBALE NOMMEE C0, ELLE REPRENDRA TOUTES LES ACTIVITES CODEES EN B1R, B2, B2_X+ RECUPERATION DE TOUS LES « AUTRES » DE CES QUESTIONS SAUF CODES AUCUNE

DUMMY
NBC0 – NOMBRE D'ACTIVITES EN C0

A TOUS

MULTIPLE

R1.1

Pour vous, quels sont les 3 sports qui représentent le mieux La Réunion et ses habitant(e)s ?

📖 *Trois réponses possibles*

- 1. **Reprendre liste B1 – 3 listes déroulantes**
- 2. Aucun **(exclusif)**
- 3. (Ne se prononce pas) **(exclusif)**

SI C0>0

SIMPLE

R20

Parmi les sports que vous pratiquez, quel est votre sport principal ou celui que vous pratiquez le plus ?

📖 *Citer - Une seule réponse possible*

PROG : AFFICHER TOUTES LES ACTIVITES PRATIQUES = CO

SI C0>0 ET R20=REPONDU

SIMPLE

R20.BIS

A combien estimez-vous votre temps de trajet aller/retour pour vous rendre sur le lieu de votre activité physique et sportive principale ?

📖 *Citer - Une seule réponse possible*

- 1. Moins de 15 minutes
- 2. Moins de 45 minutes
- 3. Plus 45 minutes
- 4. (Aucun trajet, vous pratiquez chez vous ou directement en partant de chez vous)

SI C0>0 ET R20=REPONDU

SIMPLE

R20.1

Est-ce que vous auriez envie de faire plus de cette activité physique et sportive ?

- 1. Oui
- 2. Non

SI R20.1 = 1

SIMPLE

R21

Quelles sont vos limites personnelles à la pratique de votre activité physique et sportive principale ?

📖 *ENQ : citer - Une seule réponse possible par ligne*

	Oui, tout à fait	Oui, assez	Non, pas tellement	Non, pas du tout	(Ne sait pas)	(Ne se prononce pas)
1. Votre temps libre ?	4	3	2	1	6	5
2. Vos activités professionnelles ou scolaires ?	4	3	2	1	6	5
3. Vos activités familiales ?	4	3	2	1	6	5
4. Vos autres loisirs ?	4	3	2	1	6	5
5. Votre état de santé ?	4	3	2	1	6	5
6. Votre fatigue ?	4	3	2	1	6	5

© IPSOS - CONFIDENTIAL

SI C0>0

SIMPLE

R21.1

Qu'est-ce qui rend votre activité physique et sportive principale difficile à pratiquer ?

ENQ : citer - Une seule réponse possible par ligne

	Oui, tout à fait	Oui, assez	Non, pas tellement	Non, pas du tout	(Ne sait pas)	(Ne se prononce pas)
1. Le coût des pratiques et du matériel	4	3	2	1	6	5
2. Les risques physiques	4	3	2	1	6	5
3. Le manque d'équipement et de lieux de pratique	4	3	2	1	6	5
4. La disponibilité d'accès des équipements et des lieux de pratique	4	3	2	1	6	5
5. L'éloignement des lieux de pratique	4	3	2	1	6	5
6. La durée de transport	4	3	2	1	6	5
7. La crise sanitaire de la Covid-19	4	3	2	1	6	5

SI CO = UNE ACTIVITE DE MARCHE OU DE COURSE (7, 12, 13, 14, 3, 4, 1, 2, 223, 224, 16, 10, 11, 8, 9, 15)

SIMPLE

R1

Concernant vos activités de marche/course, êtes-vous satisfait(e)…

ENQ : citer - Une seule réponse possible par ligne

Par voies praticables, il est entendu les chemins, les parcours de santé, les sentiers littoraux.

Par sentiers de randonnée, il est entendu les sentiers de grandes et de petites randonnées.

	Oui, tout à fait	Oui, assez	Non, pas tellement	Non, pas du tout	(Ne se prononce pas)
1. Du nombre de voies praticables autour de votre domicile ?	4	3	2	1	5
2. De la qualité des voies praticables autour de votre domicile ?	4	3	2	1	5
~~3. Du nombre de sentiers de randonnée sur l'île ?~~	~~4~~	~~3~~	~~2~~	~~1~~	~~5~~
4. De la qualité des sentiers de randonnée sur l'île ?	4	3	2	1	5

SI CO = UNE ACTIVITE DE CYCLE (82, 83, 193, 85, 84, 201) OU/ET UN SPORT URBAIN (87, 86, 192)

SIMPLE

R2

Êtes-vous satisfait(e)…

📖 *ENQ : citer - Une seule réponse possible par ligne*

	Oui, tout à fait	Oui, assez	Non, pas tellement	Non, pas du tout	(Ne se prononce pas)
1. Du nombre de parcours cyclables autour de votre domicile ?	4	3	2	1	5
2. De la qualité des parcours cyclables autour de votre domicile ?	4	3	2	1	5
3. Du nombre de pistes cyclables sur l'île ?	4	3	2	1	5
4. De la qualité des pistes cyclables sur l'île ?	4	3	2	1	5

A TOUS

MULTIPLE

R1.2

Si vous pouviez faire tout ce que vous voulez sans aucun empêchement, quelles sont les 3 activités physiques et sportives que vous aimeriez pratiquer ?

📖 *Trois réponses possibles*

- ❍ 1 **Liste exhaustive B1 – menu déroulant**
- ❍ 2. Aucune **(exclusif)**
- ❍ 3. (Ne se prononce pas) **(exclusif)**

SI C0>0

OUVERTE

R1.3

Pour votre propre pratique d'activité physique et sportive, quel type d'équipement sportif aimeriez-vous voir être créé près de votre domicile ?

- ❍ 1. **Ouvrir champs**
- ❍ 2. Aucun **(exclusif)**
- ❍ 3. Ne se prononce pas **(exclusif)**

SI A1 = 2 ET B2 = 72 PASSER AU BLOC D

TOP FIN MODULE B

TOP DEBUT MODULE C – VOTRE MANIERE DE PRATIQUER

POSER MODULE C POUR TOUTES ACTIVITEES CITEES EN CO

SI CO>0 ET (C0 = 3 OU 7 OU 16 OU 84 OU 193 OU 82 OU 83 OU 85 OU 89 OU 90 OU 91 OU 92 OU 95 OU 87 OU 86 OU 88 OU 192

SIMPLE

C1

Vous avez déclaré avoir pratiqué *SI C0>1* « les activités suivantes » *C0=1* < l'activité > *[activité pratiquée en C0]*.

Vous est-il arrivé d'en avoir une pratique utilitaire au cours des douze derniers mois ?

- 🕮 *Utilitaire : au sens déplacement domicile-travail, domicile-école ou lieu d'études, ou bien déplacement professionnel*
- 🕮 *ENQ : citer - Une seule réponse possible par ligne*

	Oui, une pratique exclusivement utilitaire	Oui, une pratique utilitaire et non utilitaire	Non, pas de pratique utilitaire
SI C0 = 3<br>1. Marche/balade	1	2	3
SI C0 = 7 OU 16<br>2. Jogging, footing, course à pied, running	1	2	3
SI C0 = 84 OU 193 OU 82 OU 83 OU 85<br>3. Vélo, cyclisme, VTT	1	2	3
SI C0 = 89 OU 90 OU 91 OU 92 OU 95<br>4. Moto ou quad	1	2	3
SI C0 = 87 OU 86 OU 88 OU 192<br>5. Trottinette ou roller ou skateboard ou gyropode	1	2	3

~~*SI C1_1=1 OU 2, OU C1_2=1 OU 2, OU C1_3=1 OU 2, OU C1_4=1 OU 2, OU C1_5=1 OU 2*~~

~~NUMERIC~~

~~**C1A**~~

~~**En tout, au cours des douze derniers mois, combien de fois environ avez-vous eu une pratique utilitaire de chacune de ces activités ?**~~

~~**Vous pouvez répondre en nombre de fois par semaine, par mois ou par an.**~~

- ~~🕮 *Pratique « utilitaire » : au sens déplacement domicile-travail, domicile-école ou lieu d'études, ou bien déplacement professionnel*~~
- ~~🕮 *ENQ : citer - Une seule réponse possible par ligne*~~

	~~Nombre de fois par semaine~~	~~Nombre de fois par mois~~	~~Nombre de fois par an~~	~~(Ne sait pas)~~

~~SI C1_1 = 1 OU 2~~ ~~MARCHE/BALADE~~	~~MIN=1 / MAX=30~~	~~MIN=1 / MAX=99~~	~~MIN=1 / MAX=999~~	~~0~~
~~SI C1_2 = 1 OU 2~~ ~~JOGGING, FOOTING, COURSE À PIED, RUNNING~~	~~MIN=1 / MAX=30~~	~~MIN=1 / MAX=99~~	~~MIN=1 / MAX=999~~	~~0~~
~~SI C1_3 = 1 OU 2~~ ~~VELO, CYCLISME, VTT~~	~~MIN=1 / MAX=30~~	~~MIN=1 / MAX=99~~	~~MIN=1 / MAX=999~~	~~0~~
~~SI C1_4 = 1 OU 2~~ ~~MOTO~~	~~MIN=1 / MAX=30~~	~~MIN=1 / MAX=99~~	~~MIN=1 / MAX=999~~	~~0~~
~~SI C1_5 = 1 OU 2~~ ~~TROTTINETTE OU ROLLER OU SKATEBOARD OU GYROPODE~~	~~MIN=1 / MAX=30~~	~~MIN=1 / MAX=99~~	~~MIN=1 / MAX=999~~	~~0~~

TOP DEBUT BOUCLE

PROG : POSER LES QUESTIONS C2 A C6 EN BOUCLE POUR CHAQUE ACTIVITE CITEE EN C0

Voici à présent une série de questions pour chacune des activités que vous avez déclarées.
Ces questions concernent vos pratiques non utilitaires de ces douze derniers mois.

SIMPLE

C2

Parlons de < AFFICHER ACTIVITE COCHEE EN C0 >. Au cours des 12 derniers mois, quand l'avez-vous pratiqué ?

🕮 *Si le répondant est retraité : par vacances, nous entendons une période de trois jours ou plus durant laquelle il n'était pas chez lui*

🕮 *Citer - Une seule réponse possible*

❍ 1. Tout au long de l'année, à la fois durant vos vacances et hors période de vacances
❍ 2. Seulement pendant vos vacances
❍ 3. Uniquement en dehors de vos vacances

NUMERIC

C2.1

En tout, en dehors d'une pratique utilitaire, combien de fois environ avez-vous pratiqué l'activité < AFFICHER ACTIVITE COCHEE EN C0 > au cours des douze derniers mois ?
<Si C2 = 1 ou 3> Vous pouvez répondre en nombre de fois par semaine, par mois ou par an.
<Si C2 = 2> Merci de répondre en nombre de fois par an.

🕮 *Pratique « utilitaire » : au sens déplacement domicile-travail, domicile-école ou lieu d'études, ou bien déplacement professionnel*

AFFICHER SI C2=1 ou 3 : 1./……../ fois par semaine ***(min = 1 / max = 999)***
AFFICHER SI C2=1 ou 3 : 2./……../ fois par mois ***(min = 1 / max = 999)***
AFFICHER SI C2=1 ou 3 ou 2 : 3./……../ fois par an ***(min = 1 / max = 999)***

PROG : CREER UN COMPTEUR TOTAL DE SEANCES D'APS PAR AN
NB SEANCES = SOMME DE TOUTES LES FREQUENCES DE PRATIQUE DECLAREES PAR APS EN C2.1.

SI C2.1 EST RENSEIGNEE EN NB DE FOIS PAR SEMAINE, MULTIPLIER PAR 52.
SI C2.1 EST RENSEIGNEE EN NB DE FOIS PAR MOIS, MULTIPLIER PAR 12.
SI C2.1 EST RENSEIGNEE EN NB DE FOIS PAR AN.

MULTIPLE

C3

Où avez-vous pratiqué l'activité < AFFICHER ACTIVITE COCHEE EN C0 > ?

📖 *Il peut y avoir des incohérences entre le type d'activité pratiqué (exemple le ski) et le lieu de pratique (à domicile). Dans ce cas, il faut préciser que le questionnaire a été rédigé de manière à couvrir tous les lieux de pratique, au risque d'avoir des situations improbables.*

📖 *Citer - Plusieurs réponses possibles*

- ☐ 1. Dans une installation sportive : stade, gymnase, piscine, salle de fitness ...
- ☐ 2. En milieu naturel : dans une forêt, un bois, une ravine, à la mer, sur un lac, à la montagne, à la campagne, plage ...
- ☐ 3. En ville : dans un parc, un jardin public, une rue ...
- ☐ 4. A votre domicile, ou au domicile d'une personne de votre connaissance
- ☐ 5. Sur votre lieu de travail, d'études ou à proximité
- ☐ 6. Un autre lieu de pratique

MULTIPLE

C4

Toujours en dehors d'une pratique utilitaire, avez-vous pratiqué l'activité < AFFICHER ACTIVITE COCHEE EN C0 > ...

📖 *Citer - Plusieurs réponses possibles*

📖 *Pratique « utilitaire » : au sens déplacement domicile-travail, domicile-école ou lieu d'études, ou bien déplacement professionnel*

- ☐ 1. Entre amis
- ☐ 2. En famille ou en couple
- ☐ 4. Avec des relations de travail ou d'études
- ☐ 5. Avec des membres d'une application communautaire ou d'un réseau social
- ☐ 6. Avec d'autres sportifs pratiquant la même activité
- ☐ 7. Avec d'autres personnes que celles indiquées précédemment
- ☐ 3. Seul(e)

SIMPLE

C5

A quel âge avez-vous débuté l'activité < AFFICHER ACTIVITE COCHEE EN C0 >, même si vous l'avez interrompue pendant une certaine période ?

📖 *Ne pas citer – recoder en fonction de la réponse - Une seule réponse possible*

- ❍ 1. 5 ans ou moins
- ❍ 2. 6 – 10 ans
- ❍ 3. 11 – 14 ans
- ❍ 4. 15 – 19 ans
- ❍ 5. 20 – 29 ans
- ❍ 6. 30 – 39 ans
- ❍ 7. 40 – 49 ans
- ❍ 8. 50 – 59 ans

- 9. 60 – 69 ans
- 10. 70 ans et plus

SIMPLE

C6

Pour l'activité < AFFICHER ACTIVITE COCHEE EN C0 >, quel niveau vous donneriez-vous ?

Une seule réponse possible

- 1. Débutant
- 2. Débrouillard
- 3. Intermédiaire
- 4. Avancé
- 5. Expert

FIN DE LA BOUCLE

TOP FIN DE LA BOUCLE

PROG : LES QUESTIONS C8 A C13D SERONT PASSEES EN UNE SEULE FOIS POUR L'ENSEMBLE DES ACTIVITES CITEES EN C0 (DE 1 A N ACTIVITES) / POSER C8 A C15BIS, SI C0>1

Les questions complémentaires qui suivent sont relatives à l'ensemble de vos pratiques physiques et sportives utilitaires et non utilitaires de ces douze derniers mois.

SIMPLE

C8

Avez-vous pratiqué SI PLUSIEURS ACTIVITES CITEES EN CO AFFICHER < une ou plusieurs de ces activités > / SI UNE SEULE ACTIVITE CITEE EN B1R, B2, B2_X AFFICHER < cette activité > en tant que membre d'un club sportif public ou d'une association sportive ou d'une structure privée à caractère commercial de type centre de fitness, d'un club de gym, d'un centre de remise en forme, … ?

Citer

- 1. Oui
- 2. Non

SI C8=2

SINGLE

C8NEW

Cette non-affiliation est-elle consécutive à la crise de la Covid-19 ?

- 1. Oui
- 2. Non

SI C8 = 1

MULTIPLE

C8.1

Pouvez-vous préciser pour quelles activités physiques et sportives ?

Citer - Plusieurs réponses possibles

- 1. **PROG : FAIRE APPARAITRE A L'ECRAN LES ACTIVITES CITEES EN C0**
- 2. Autre : **PROG CHAMP OUVERT**

SI C8 = 1

MULTIPLE

C8.2

Pouvez-vous préciser le type de structure à laquelle vous êtes adhérent(e) pour l'activité..?

Citer - Plusieurs réponses possibles

	Une association sportive ou un club sportif public	Une structure privée à caractère commercial de type centre de fitness, club de golf, centre équestre
AFFICHER ACTIVITE CITEE EN C8.1	1	2
AFFICHER ACTIVITE CITEE EN C8.1 ...	1	2

SIMPLE

C9

Pour la pratique SI PLUSIEURS ACTIVITES CITEES EN CO AFFICHER < d'une ou plusieurs de ces activités > / SI UNE SEULE ACTIVITE CITEE EN B1R, B2, B2_X AFFICHER < de cette activité >, avez-vous été encadré(e) par un coach, un entraîneur, moniteur, professeur, éducateur ou animateur, toujours dans les douze derniers mois ?

« Encadré(e) par quelqu'un présent à vos côtés lorsque vous pratiquez. »

- 1. Oui
- 2. Non

SI C9 = 1 ET SI C0 > 1 ACTIVITE

MULTIPLE

C9.1.1

Pouvez-vous préciser pour quelles activités vous avez été encadré ?

PROG : FAIRE APPARAITRE A L'ECRAN LES ACTIVITES CITEES EN C0

Citer - Plusieurs réponses possibles

SIMPLE

C10

Pour la pratique SI PLUSIEURS ACTIVITES CITEES EN CO AFFICHER < d'une ou plusieurs de ces activités > / SI UNE SEULE ACTIVITE CITEE EN B1R, B2, B2_X AFFICHER < de cette activité >, avez-vous eu une ou plusieurs licences sportives délivrées par des fédérations sportives durant ces douze derniers mois ?

Être détenteur d'une carte d'adhésion à un club ou une association ne signifie pas automatiquement avoir une licence sportive.

❍ 1. Oui
❍ 2. Non

SI C10=2

SINGLE

C10NEW

Est-ce consécutif à la crise de la Covid-19 ?

❍ 1. Oui
❍ 2. Non

SINGLE

C11

Avant la crise sanitaire de la Covid-19, pratiquiez-vous SI PLUSIEURS ACTIVITES CITEES EN C0 AFFICHER < une ou plusieurs de ces activités > / SI UNE SEULE ACTIVITE CITEE EN B1R, B2, B2_X AFFICHER < cette activité sportive > dans le cadre d'une compétition officielle ou d'un tournoi ?

❍ 1. Oui
❍ 2. Non

MULTIPLE

C13

Au cours des douze derniers mois, dans le cadre de votre pratique d'activité(s) physique(s) et sportive(s), utilitaire ou non utilitaire, vous est-il arrivé …

PROG : ITEM « NON » EXCLUSIF / MAX 2 REPONSES POSSIBLES PAR LIGNE + ALEA SOUS-QUESTIONS 1 A 5

- *Une seule réponse par ligne*
- *Pratique « utilitaire » : au sens déplacement domicile-travail, domicile-école ou lieu d'études, ou bien déplacement professionnel*

	Oui, pendant votre activité	Oui, avant ou après votre activité	Oui, tout le temps	Non (EXCLUSIF)
1. … d'utiliser un objet connecté hors smartphone (montre, bracelet, capteur …) ?	1	2	3	4
2. … d'utiliser une application internet ou mobile de sport/fitness (par exemple Runkeeper, Strava, Swimming Heroes, Fizzup, Fitbit, Pocket Yoga, Visorando, Endomondo …) ?	1	2	3	4
3. … d'utiliser les réseaux sociaux/sites de rencontres amicales pour organiser votre pratique (Tik Tok, Facebook, Instagram, Snapchat, Whatsapp, OVS …) ?	1	2	3	4
4. … de suivre ou regarder les contenus d'un blogueur, d'un youtubeur, un influenceur ?	1	2	3	4
5. … de suivre ou regarder un tutoriel sur Internet ?	1	2	3	4

~~SINGLE~~

~~C13B~~

~~Au cours des douze derniers mois, dans le cadre de votre pratique d'activité(s) physique(s) et sportive(s), utilitaire ou non utilitaire, vous est-il arrivé de vous abonner à un service d'accompagnement et de conseils personnalisés en ligne avec un entraineur sportif ?~~

~~ENQ : citer - Une seule réponse possible~~

- ~~1. Oui~~
- ~~2. Non~~

~~*SI C13B=1*~~

~~SINGLE~~

~~C13C~~

~~Ce service d'accompagnement en ligne proposait-il des cours par vidéo …~~

~~ENQ : citer - Une seule réponse possible~~

- ~~1. En direct~~
- ~~2. En différé~~
- ~~3. Les 2~~

~~*SI C13B=1*~~

~~SINGLE~~

~~C13D~~

~~Pouvez-vous discuter de vos performances avec votre entraineur sportif de vive voix ?~~

~~ENQ : citer - Une seule réponse possible~~

- ~~1. Oui~~
- ~~2. Non~~

SI C0>0

SIMPLE

R19.2

Trouvez-vous que votre dépense pour les activités physiques et sportives est ?

ENQ : citer - Une seule réponse possible

- 1. Très importante
- 2. Importante
- 3. Suffisante
- 4. Faible
- 5. Très faible
- 6. (Ne se prononce pas)

R19.3 ET R19.4 EN TRIGGER

SI CO>0

MULTIPLE

R19.3

Ces 12 derniers mois, quelles sont les sorties de loisirs sportifs que vous avez achetées personnellement à La Réunion ?

Enq : citer les activités

Citer : Plusieurs réponses possibles

PROG : ALEA DE 1 A 21 EN REGROUPANT ITEMS 17, 18 ET 19

- ☐ 1. Parapente, chute libre
- ☐ 2. Plongée
- ☐ 3. Escalade, alpinisme

- ☐ 4. Canyoning
- ☐ 5. Tunnel de lave
- ☐ 6. Guide randonnée
- ☐ 7. Guide touristique
- ☐ 8. Nage en eau vive
- ☐ 9. Raft
- ☐ 10. Karting
- ☐ 11. Quad
- ☐ 12. Sortie en mer
- ☐ 13. Scooter des mers
- ☐ 14. VTT, VAE (vélo électrique), Cycle
- ☐ 15. Accro Branche
- ☐ 16. Saut à l'élastique
- ☐ 17. Randonnée aquatique (PLM)
- ☐ 18. Randonnée aquatique en paddle
- ☐ 19. Randonnée aquatique en canoë
- ☐ 20. Pêche au gros
- ☐ 21. Surf
- ☐ 22. Autre
- ☐ 23. Aucune activité achetée personnellement **(EXCLUSIF)**

SI C0>0 ET R19.3 = AU MOINS UNE REPONSE ET R19.3<>23 (AUCUNE)

NUMERIC

R19.4

Combien d'argent avez-vous dépensé pour la dernière activité de loisir sportif que vous avez achetée ?

1. Réponse libre

- ○ 99. (Ne se prononce pas) **Exclusif**
- ○ 999. (Refus de répondre) **Exclusif**

CO>0

SIMPLE

C15

Au cours des 12 derniers mois, pour faire une activité physique ou sportive êtes-vous allé dans les lieux en accès libre suivants ou avez-vous utilisé les équipements suivants ?

Lieux en accès libre : accessibles à tous sans frais ou autre restriction

Une seule réponse par ligne

	Oui	Non
1. Parcours de santé, parcours sportif, aire de fitness ou musculation	1	2
2. Skate-park	1	2
3. City-stade, plateau multisports (basket + foot + …)	1	2
4. Table de ping-pong, court de tennis	1	2
5. Terrain de pétanque	1	2
6. Lagon et plage	1	2
7. Rivière et bassin	1	2
8. Sentier et chemin	1	2

SI C15_1 A C15_8 AU MOINS UN « OUI »

SIMPLE

C15BIS

A quelle fréquence au cours des douze derniers mois ?

Une seule réponse par ligne

	Moins d'une fois par mois	Une à trois fois par mois	Une fois par semaine	Plusieurs fois par semaine
SI C15_1 = OUI 1. Parcours de santé, parcours sportif, aire de fitness ou musculation	1	2	3	4
SI C15_2 = OUI 2. Skate-park	1	2	3	4
SI C15_3 = OUI 3. City-stade, plateau multisports (basket + foot + …)	1	2	3	4
SI C15_4 = OUI 4. Table de ping-pong, court de tennis	1	2	3	4
SI C15_5 = OUI 5. Terrain de pétanque	1	2	3	4
SI C15_6 = OUI 6. Lagon et plage	1	2	3	4
SI C15_7 = OUI 7. Rivière et bassin	1	2	3	4
SI C15_8 = OUI 8. Sentier et chemin	1	2	3	4

NUMERIC

C16

NB_SEANCES PAR AN :

TOP FIN MODULE C

TOP DEBUT MODULE D – VOTRE MOTIVATION ET VOTRE PRATIQUE EN GENERAL

Les questions qui suivent concernent votre rapport général à la pratique d'activités physiques ou sportives.

SI (A1 = 2 ET C0=0) OU C16 < 54

SIMPLE

D1

A l'aide d'une échelle allant de 0 à 5, 0 voulant dire « Non pas du tout » et 5 voulant dire « Oui tout à fait », si vous ne pratiquez Si A1 = 2 et B2 = 72 AFFICHER < aucune activité physique ou sportive aujourd'hui> / SI AU MOINS UNE ACTIVITE EN C0 AFFICHER < pas davantage d'activités physiques ou sportives aujourd'hui >, est-ce parce que … :

PROG : ROTATION DES ITEMS 1 A 17

ENQ : citer - Une seule réponse possible par ligne

	0 Non, pas du tout	1	2	3	4	5 Oui, tout à fait
1. Vous préférez d'autres activités	1	2	3	4	5	6
2. Vous n'aimez pas le sport	1	2	3	4	5	6
3. Vous avez eu une mauvaise expérience dans le passé (notamment à l'école, en club…)	1	2	3	4	5	6
4. Vous en faites déjà suffisamment PROG : Proposer cet item si NB_SEANCES < 54 et > 0	1	2	3	4	5	6
5. Vous avez des contraintes professionnelles ou scolaires	1	2	3	4	5	6
6. Vous avez des contraintes familiales	1	2	3	4	5	6
7. Cela coûte trop cher	1	2	3	4	5	6
8. Vous n'arrivez pas à vous y mettre	1	2	3	4	5	6
9. Les lieux pour pratiquer sont trop éloignés	1	2	3	4	5	6
10 Les lieux pour pratiquer ne sont pas adaptés	1	2	3	4	5	6
11. Ce que proposent les clubs et associations ne vous convient pas	1	2	3	4	5	6
12. Vous avez des problèmes de santé	1	2	3	4	5	6
13. Votre métier est ou a été physiquement dur	1	2	3	4	5	6
14. Vous ne connaissez personne avec qui pratiquer	1	2	3	4	5	6
15. Le regard des autres est difficile à supporter	1	2	3	4	5	6
16. Vous avez des difficultés à être accepté(e) par les autres	1	2	3	4	5	6
17. Vous manquez de temps	1	2	3	4	5	6
18. Pour d'autres raisons	1	2	3	4	5	6

SI (A1 = 2 ET C0=0) AUCUNE ACTIVITE SPORTIVE

SIMPLE

D2

Avez-vous envie de pratiquer une activité physique ou sportive ?

- 1. Oui
- 2. Non

SI (A1 = 2 ET C0=0) AUCUNE ACTIVITE SPORTIVE

SIMPLE

D3

Avez-vous par le passé pratiqué une ou plusieurs activités physiques ou sportives, hors activités physiques obligatoires à l'école ?

- 1. Oui
- 2. Non

SI D3 = 1

MULTIPLE

D3.1

Lesquelles ?

- 🕮 *Plusieurs réponses possibles*
- 🕮 *Ne pas citer*

LISTE B1

SI C0 > 0

SIMPLE

D4

En prenant en compte l'ensemble de vos activités déclarées au cours des 12 derniers mois, diriez-vous, qu'en moyenne, vous pratiquez vos activités physiques et sportives …

- 🕮 *Prendre en compte toutes les activités pratiquées (utilitaires et non-utilitaires) au cours des douze derniers mois.*
- 🕮 *ENQ : citer - Une seule réponse possible*

- ❍ 1. Moins d'une fois par mois
- ❍ 2. Une à trois fois par mois
- ❍ 3. Une fois par semaine
- ❍ 4. 2 à 3 fois par semaine
- ❍ 5. Plus de 3 fois par semaine
- ❍ 6. (Je ne sais pas)

~~*SI C0 > 0*~~

~~**SIMPLE**~~

~~**D4.2**~~

~~**En prenant en compte l'ensemble de vos activités déclarées au cours des 12 derniers mois, pensez-vous avoir suffisamment de temps pour pratiquer vos activités physiques et sportives ?**~~

- ~~🕮 *Prendre en compte toutes les activités pratiquées (utilitaires et non-utilitaires) au cours des douze derniers mois.*~~

- ❍ ~~1. Tout à fait d'accord~~
- ❍ ~~2. Plutôt d'accord~~
- ❍ ~~3. Indifférent~~
- ❍ ~~4. Plutôt pas d'accord~~
- ❍ ~~5. Pas du tout d'accord~~
- ❍ ~~6. (Je ne sais pas)~~

~~*SI C0 > 0*~~

~~**SIMPLE**~~

~~**D4.3**~~

~~**Arrivez-vous à trouver un équilibre dans votre quotidien entre vos contraintes familiales et professionnelles (ou scolaires) et la pratique de toutes vos activités physiques et sportives ?**~~

- ~~🕮 *Prendre en compte toutes les activités pratiquées (utilitaires et non-utilitaires) au cours des douze derniers mois.*~~

- ❍ ~~1. Tout à fait d'accord~~
- ❍ ~~2. Plutôt d'accord~~
- ❍ ~~3. Indifférent~~
- ❍ ~~4. Plutôt pas d'accord~~

❍ ~~5. Pas du tout d'accord~~
❍ ~~6. (Je ne sais pas)~~

SI C0>0

SIMPLE

D5

A l'aide d'une échelle allant de 0 à 5, 0 voulant dire « Non pas du tout » et 5 voulant dire « Oui tout à fait », diriez-vous qu'en pratiquant une activité physique ou sportive, vous recherchez :

PROG : ROTATION DES ITEMS 1 A 13

ENQ : citer - Une seule réponse possible par ligne

	0 Non pas du tout	1	2	3	4	5 Oui tout à fait
1. Le risque	1	2	3	4	5	6
2. La compétition	1	2	3	4	5	6
3. La rencontre avec les autres, être avec vos proches	1	2	3	4	5	6
4. La santé	1	2	3	4	5	6
5. Le contact avec la nature	1	2	3	4	5	6
6. La dépense physique	1	2	3	4	5	6
7. Le plaisir, l'amusement	1	2	3	4	5	6
8. L'entretien physique	1	2	3	4	5	6
9 L'amélioration de vos performances personnelles	1	2	3	4	5	6
10. Être bien dans votre peau, évacuer le stress	1	2	3	4	5	6
11. L'amélioration de votre apparence physique (se muscler, mincir, garder la ligne)	1	2	3	4	5	6
12. L'aventure	1	2	3	4	5	6
13. Rien en particulier mais vous devez avoir une activité physique	1	2	3	4	5	6
14. Autre chose	1	2	3	4	5	6

TOP FIN MODULE D

TOP DEBUT MODULE E – ACCIDENT LORS DE LA PRATIQUE

POSER E1 A E9 SI C0>0

SIMPLE

E1

Dans les douze derniers mois, avez-vous été victime d'un accident pendant la pratique d'une activité physique ou sportive, comme une fracture, une entorse, un claquage, une tendinite, une perte de connaissance, un malaise ... ?

❍ 1. Oui

- 2. Non
- 3. (Je ne souhaite pas répondre)

SI E1 = 1 ET C0 > 0 ACTIVITE

MULTIPLE

E2

Pendant la pratique de quelle(s) activité(s) avez-vous eu cet accident de santé ?

PROG : FAIRE APPARAITRE A L'ECRAN LES ACTIVITES CITEES EN C0

PROG : AJOUT DE LA MODALITE « ACTIVITE NON DECLAREE (NE PAS CITER) »

- *Plusieurs réponses possibles*
- *Lorsque le répondant se souvient d'avoir eu un accident sur une activité non citée au début, merci de cocher « Activité non déclarée ». Les questions suivantes feront référence à cette activité déclarée en cours d'entretien. »*

- Activité non déclarée

POSER E3 A E6 POUR CHAQUE ACTIVITE CITEE EN E2 (Y COMPRIS « ACTIVITE NON DECLAREE »

Nous allons maintenant évoquer vos accidents sportifs.

SI E2 = 1 OU > 1

~~SIMPLE~~

~~**E3**~~

~~**Concernant [activité]**~~

~~**Les services de secours ont-ils été appelés à la suite de cet accident (SAMU, ambulance, pompiers, pisteurs secouristes, maîtres-nageurs sauveteurs, etc.) ?**~~

- ~~1. Oui~~
- ~~2. Non~~
- ~~3. Je ne souhaite pas répondre~~

~~SIMPLE~~

~~**E5**~~

~~**Avez-vous consulté un médecin à la suite de cet accident ?**~~

- ~~1. Oui~~
- ~~2. Non~~
- ~~3. Je ne souhaite pas répondre~~

~~SIMPLE~~

~~**E4**~~

~~**Avez-vous été hospitalisé à la suite de cet accident ?**~~

- ~~1. Oui~~
- ~~2. Non~~
- ~~3. Je ne souhaite pas répondre~~

SIMPLE

E6

Cet accident a-t-il provoqué un arrêt de votre activité professionnelle ou scolaire ?

- 1. Oui

- 2. Non
- 3. (Non concerné(e), vous n'aviez pas d'activité professionnelle ou scolaire à ce moment-là)

TOP E2

Comme vous le savez, depuis Mars 2020, la crise sanitaire liée à la Covid-19 a fortement transformé la vie des Réunionnais. Je vais vous poser quelques questions sur la manière dont cette crise a modifié la pratique de vos activités physiques et sportives.

SIMPLE

E7

Parmi les activités physiques et sportives que vous avez déclarées, est-ce que vous avez nouvellement débuté ou repris une de ces activités en raison de la crise Covid-19 ?

- 1. Oui
- 2. Non
- 3. (Ne sait pas)

SI E7=1

MULTIPLE

E8

<si C0=1> Laquelle <si C0>1> lesquelles ?

AFFICHER LA LISTE DES SPORTS RECODE EN C0 ET SAISIE EN SPONTANEE SUR CETTE LISTE, SAUF SI HESITATION DE L'ENQUETE ALORS LECTURE DE LA LISTE.

DUMMY
NBE8 – NOMBRE D'ACTIVITES EN E8
DIFF = NBC0-NBE8

SI E7=1 ET DIFF>=1

SIMPLE

E9

Depuis cette crise sanitaire, votre temps de pratique a diminué, augmenté ou est inchangée pour…

AFFICHER LA LISTE DES ACTIVITES CITEES EN C0 EN RETIRANT TOUTES CELLES CITEES EN E8

	A diminué	A augmenté	Est inchangé	(Ne sait pas)
Afficher activité 1	1	2	3	4
Afficher activité 2	1	2	3	4
Afficher activité …	1	2	3	4

A TOUS (PRATIQUANTS ET NON-PRATIQUANTS)

SIMPLE

E10

En raison de la crise sanitaire avez-vous complètement abandonné une activité physique et sportive ?

- Oui
- Non

SI E10=1

MULTIPLE

E11

Laquelle ou lesquelles ?

AFFICHAGE DE LA LISTE DES SPORTS B1

Plusieurs réponses possibles

TOP FIN MODULE E

TOP DEBUT MODULE F – VOS HABITUDES DE VIE ET VOTRE SANTE

Nous allons maintenant évoquer vos habitudes de vie et votre santé.

A TOUS

SIMPLE

F1.NEW

Vous est-il arrivé d'utiliser un moyen de déplacement électrique ou avec assistance électrique au cours des douze derniers mois ?

ENQ : citer

- 1. Oui
- 2. Non

SI F1_NEW=1

MULTIPLE

F1.NEW2

Quels moyens de déplacement électrique avez-vous utilisés ?

ENQ : citer – plusieurs réponses possibles

- ☐ 1. Le vélo
- ☐ 2. Le VTT
- ☐ 3. La trottinette
- ☐ 4. Un autre moyen (skate, gyropode, hoverboard, segway, etc.)

SI F1_NEW=1

SIMPLE

F1BIS

A quelle fréquence au cours des douze derniers mois, avez-vous utilisé un moyen de déplacement électrique ou avec assistance électrique de type … ?

Une seule réponse possible par ligne

	Moins d'une fois par semaine	Une fois par semaine	Plus d'une fois par semaine
SI F1_NEW2 = 1<br>**F1bis_1** : Vélo	1	2	3
SI F1_NEW2 = 2<br>**F1bis_2** : VTT	1	2	3
SI F1_NEW2 = 3<br>**F1bis_3** : Trottinette	1	2	3
SI F1_NEW2 = 4<br>**F1bis_4** : Autre (skate, gyropode, hoverboard, segway, etc.)	1	2	3

A TOUS

SIMPLE

F2

Dans le cadre de votre activité principale, lors d'une journée habituelle…

ENQ : citer - Une seule réponse possible par ligne

ENQ : L'activité principale regroupe les choses que vous avez à faire au quotidien, qu'elles soient rémunérées ou non : votre activité professionnelle, l'entretien de votre maison, vous occuper de votre famille, étudier ou vous former, etc.

	Tout le temps	Souvent	Quelquefois	Jamais
1. Etes-vous assis(e)	1	2	3	4
2. Etes-vous debout	1	2	3	4
3. Marchez-vous	1	2	3	4
4. Portez-vous ou transportez-vous des charges (restauration, déménagement, etc.)	1	2	3	4
5. Mobilisez-vous une activité physique ou sportive (coursier(e) à vélo, sportif(ve) professionnel(le) ou en sport-études, militaire, pompier(e))	1	2	3	4

A TOUS

SIMPLE

F3.NEW

Lorsque vous êtes chez vous, lors d'une semaine habituelle, en moyenne combien de temps consacrez-vous aux activités suivantes ?

PROG : REPONSE OBLIGATOIRE POUR CHAQUE LIGNE

MIN 0 MAX 90.0

Nombre en décimal

Vous pouvez écrire des nombres décimaux, par exemple 0,5 pour une demi-heure.

F3NEW_1 Ménage : __/ heures

❍ 999. Je ne m'en occupe pas

F3NEW_2 Jardinage : __/ heures

❍ 999. Je ne m'en occupe pas

F3NEW_3 Bricolage : __/ heures

❍ 999. Je ne m'en occupe pas

~~*A TOUS*~~

~~SIMPLE~~

~~**F4_NEW**~~

~~**Au cours des douze derniers mois avez-vous joué sur ordinateur, console, tablette ou smartphone (hors simulateur d'activité physique type Wii-sport) …**~~

~~*ENQ : citer - Une seule réponse possible par ligne*~~

© IPSOS - CONFIDENTIAL

	Oui	Non
F4_1NEW : à un jeu vidéo de sport	1	2
F4_2NEW : à un autre type de jeu vidéo	1	2

SI FA_NEW = 1 OU > 1 (AU MOINS UN OUI)

SIMPLE

F4BIS

Au cours des douze derniers mois, avez-vous joué … ?

ENQ : citer - Une seule réponse possible par ligne

	Tous les jours ou presque	Plusieurs fois par semaine	Une fois par semaine	Moins souvent
SI F4_1NEW = OUI<br>F4bis_1 : à un jeu vidéo de sport	1	2	3	4
SI F4_2 NEW = OUI<br>F4bis_2 : à un autre type de jeu vidéo	1	2	3	4

SI FA_NEW = 1 OU > 1 (AU MOINS UN OUI)

SIMPLE

F4.1NEW

Avez-vous participé à un tournoi ou une compétition de jeux vidéo au cours des douze derniers mois…

ENQ : Une seule réponse possible par ligne

	Oui	Non
F4.1NEW_1 : Entre amis ou en famille, dans la même pièce	1	2
F4.1 NEW_2 : En réseau à distance avec des amis, de la famille ou d'autres joueurs	1	2
F4.1 NEW_3 : En vous déplaçant sur les lieux d'une compétition officielle	1	2

SI F4.1_NEW = 1 OU > 1 (AU MOINS UN OUI)

SIMPLE

F4.1BIS

Au cours des 12 derniers mois, vous y avez participé…

ENQ : citer - Une seule réponse possible par ligne

	Tous les jours ou presque	Plusieurs fois par semaine	Une fois par semaine	Moins souvent
SI F4.1 NEW_1 = OUI<br>F4.1bis_1 : Entre amis ou en famille, dans la même pièce	1	2	3	4
SI F4.1 NEW_2 = OUI<br>F4.1bis_2 : En réseau à distance avec des amis, de la famille ou d'autres joueurs	1	2	3	4

© IPSOS - CONFIDENTIAL

~~SI F4.1 NEW_3 = OUI~~<br>~~F4.1bis_3 : En vous déplaçant sur les lieux d'une compétition officielle~~	~~1~~	~~2~~	~~3~~	~~4~~

A TOUS

SIMPLE

F6

Au cours de la semaine dernière, en dehors de votre travail ou de vos études, en moyenne combien d'heures par jour avez-vous regardé un écran (télévision, smartphone, ordinateur ou tablette) ?

🕮 *ENQ : citer - Une seule réponse possible par ligne*

	Moins de 3 heures par jour	De 3 à 7 heures par jour	Plus de 7 heures par jour
1. Du lundi au vendredi	1	2	3
2. Le samedi et le dimanche	1	2	3

Afin de mieux cerner le lien qui existe entre santé et activité physique, parlons brièvement de votre santé.

A TOUS

SIMPLE

F7

Comment est votre état de santé en général ?

🕮 *ENQ : citer - Une seule réponse possible*

- ❍ 1. Très bon
- ❍ 2. Bon
- ❍ 3. Assez bon
- ❍ 4. Mauvais
- ❍ 5. Très mauvais
- ❍ 6. (Je ne sais pas) **EXCLUSIF**
- ❍ 7. (Je ne souhaite pas répondre) **EXCLUSIF**

A TOUS

SIMPLE

F8

Avez-vous une maladie ou un problème de santé qui soit chronique ou de caractère durable ?

🕮 *Chronique signifie que le problème de santé dure depuis au moins 6 mois*

- ❍ 1. Oui
- ❍ 2. Non
- ❍ 3. (Je ne sais pas) **EXCLUSIF**
- ❍ 4. (Je ne souhaite pas répondre) **EXCLUSIF**

A TOUS

SIMPLE

F9

Etes-vous limité(e), depuis au moins 6 mois, à cause d'un problème de santé, dans les activités que les gens font habituellement ?

🕮 *ENQ : citer - Une seule réponse possible*

- 1. Oui, fortement limité(e)
- 2. Oui, limité(e), mais pas fortement
- 3. Non, pas limité(e) du tout
- 4. (Je ne sais pas) **EXCLUSIF**
- 5. (Je ne souhaite pas répondre) **EXCLUSIF**

A TOUS

SIMPLE

F9BIS

Êtes-vous en situation de handicap ?

- 1. Oui
- 2. Non
- 3. (Ne souhaite pas répondre) **EXCLUSIF**

SI F9BIS = OUI

SIMPLE

F9NEW2

Avez-vous accès à un lieu où vous pouvez pratiquer une activité physique et sportive aménagées pour votre handicap ?

- 1. Oui
- 2. Non

SI F9NEW2 = OUI

SIMPLE

F9NEW2BIS

Est-ce que vous êtes satisfait du nombre d'activités sportives adaptées auxquelles vous pouvez participer ?

- 1. Oui
- 2. Non
- 3. (Ne se prononce pas) **EXCLUSIF**

A TOUS

NUMERIC

F10

Quelle est votre taille ?

🕮 *La connaissance du profil physique est importante dans l'analyse des choix d'activités physiques ou sportives.*
🕮 *En cm, pieds nus*
🕮 *Inscrivez ici uniquement des chiffres*

© IPSOS - CONFIDENTIAL

PROG : BORNES 60-280

/ / cm

- ❍ Je ne souhaite pas répondre **EXCLUSIF**

NUMERIC

F11

Quel est votre poids ?

- 🕮 *La connaissance du profil physique est importante dans l'analyse des choix d'activités physiques ou sportives.*
- 🕮 *En kg, sans vêtement, ni chaussure*
- 🕮 *Inscrivez ici uniquement des chiffres*
- 🕮 ***Consigne pour les femmes enceintes** : « Merci d'indiquer votre poids avant la grossesse. »*

PROG : BORNES 20-300

/ / Kg

- ❍ (Je ne souhaite pas répondre) **EXCLUSIF**

TOP FIN MODULE F

TOP DEBUT MODULE R – QUESTION ILE DE LA REUNION

A TOUS

SIMPLE

R11.2

Est-ce que vous faites des paris sportifs ?

- 1. Oui
- 2. Non
- 3. (Refus de répondre) **EXCLUSIF**

SI R11.2 = 1

OUVERTE

R12

Sur quel(s) sport(s) ?

ENQ : saisir un sport par ligne

MAX 7

SI NOMBRE DE REPONSES A R12 >1

SINGLE

R12B

Parmi les sports sur lesquels vous faîtes des paris, quel est celui où vous pariez le plus ?

Une seule réponse possible

AFFICHER REPONSES EN R12

SI R11.2 = 1

MULTIPLE

R12.1

Par quel(s) moyen(s) faites-vous des paris sportifs ?

ENQ : citer - Plusieurs réponses possibles

- 1. Presse/tabac
- 2. Bar/PMU
- 3. Smartphone/tablette
- 4. Ordinateur/télévision
- 5. Gallodrome/cercle de combat de coq
- 6. Autre
- 7. (Ne se prononce pas) **(EXCLUSIF)**

© IPSOS - CONFIDENTIAL

SI R11.2 = 1

SIMPLE

R12.2

Quelle est votre fréquence de pari sportif ?

ENQ : citer - Une seule réponse possible

- 1. 1 à plusieurs fois par jour
- 2. 1 à plusieurs fois par semaine
- 3. 1 à plusieurs fois par mois
- 4. 1 à plusieurs fois par an
- 5. (Ne se prononce pas) **EXCLUSIF**

SI R11.2 = 1

SIMPLE

R12.3

Faites-vous des paris sportifs en tenant compte …

ENQ : citer - Une seule réponse possible par ligne

ALEA ITEMS

	Tout le temps	*Souvent*	*Quelquefois*	*Jamais*	*(Ne se prononce pas)*
1. Des cotes de pari	1	2	3	4	5
2. De l 'information sportive	1	2	3	4	5
3. De votre connaissance du sport	1	2	3	4	5
4. De votre vécu sportif	1	2	3	4	5
5. De votre intuition	1	2	3	4	5
6. Des équipes ou sportifs que vous supportez	1	2	3	4	5

SI R11.2 = 1

NUMERIC

R12.6

Combien avez-vous misé au-cours des 7 derniers jours ?

Recoder dans l'échelle le montant donné par le répondant

- 1. Moins de 50 euros
- 2. De 50 à moins de 100 euros
- 3. De 100 à moins de 250 euros
- 4. De 250 à moins de 500 euros
- 5. De 500 à moins de 750 euros
- 6. De 750 à moins de 1000 euros
- 7. 1000 euros ou plus
- 8. (N'a pas misé au cours des 7 derniers jours)
- 9. (Ne se prononce pas) **EXCLUSIF**

- *Perception et bien-être liés au sport à La Réunion*

SI C0>0

SIMPLE

R22

Tout bien considéré, dites-moi dans quelle mesure vous êtes satisfait(e) de vos activités sportives actuellement ? Donnez une note sur une échelle de 1 à 10, où 1 signifie que vous n'êtes pas du tout satisfait(e) et 10 que vous êtes tout à fait satisfait(e), les notes intermédiaires permettent de nuancer votre jugement.

- ❍ 1 - vous n'êtes pas du tout satisfait(e)
- ❍ 2
- ❍ 3
- ❍ 4
- ❍ 5
- ❍ 6
- ❍ 7
- ❍ 8
- ❍ 9
- ❍ 10 - vous êtes tout à fait satisfait(e)

SI C0>0

SIMPLE

R22.1

Tout bien considéré, dans quelle mesure diriez-vous que vous êtes un sportif heureux ou une sportive heureuse ? Donnez une note sur une échelle de 1 à 10, où 1 signifie que vous n'êtes pas du tout un sportif heureux ou une sportive heureuse et 10 que vous êtes tout à fait un sportif heureux ou une sportive heureuse, les notes intermédiaires permettent de nuancer votre jugement.

- ❍ 1 - vous n'êtes pas du tout un sportif heureux
- ❍ 2
- ❍ 3
- ❍ 4
- ❍ 5
- ❍ 6
- ❍ 7
- ❍ 8
- ❍ 9
- ❍ 10 - vous êtes tout à fait un sportif heureux

- *Question sur la langue*

A TOUS

SIMPLE

R23

Quelle langue parliez-vous à la maison à l'âge de 5 ans ?

ENQ : citer - Une seule réponse possible

- ❍ 1. Uniquement créole
- ❍ 2. Uniquement français
- ❍ 3. Français et créole
- ❍ 4. Français et autres langues
- ❍ 5 Créole et autre langue
- ❍ 6. Autres langues

© IPSOS - CONFIDENTIAL

SI C0>0

SIMPLE

R23.1

Dans votre pratique sportive principale, est-ce que vous parlez ?

ENQ : citer - Une seule réponse possible

- 1. Uniquement créole
- 2. Uniquement français
- 3. Français et créole
- 4. Français et autres langues
- 5. Créole et autres langues
- 6. Autres langues

TOP FIN MODULE R

TOP DEBUT MODULE G – MIEUX VOUS CONNAITRE

A TOUS

Pour mieux analyser les résultats de l'enquête, voici un certain nombre de questions qui permettront de mieux vous connaître.

A TOUS

SIMPLE

G4

Quel est le diplôme le plus élevé que vous avez obtenu ?

Citer - Une seule réponse possible

- 1. Aucun diplôme
- 2. Certificat d'études primaire (CEP) ou diplôme étranger de même niveau
- 3. Brevet des collèges, BEPC, brevet élémentaire ou diplôme étranger de même niveau
- 4. CAP, BEP ou diplôme de même niveau
- 5. BACCALAUREAT général, technologique ou professionnel, ou diplôme étranger de même niveau
- 6. Diplôme de la santé, du travail social et du sport de niveau bac
- 7. Capacité en droit, DAEU, ESEU
- 8. Diplôme de niveau Bac+2 (DEUG, BTS, DUT, diplômes d'infirmier(e)s jusqu'à 2011, de kinésithérapeute, d'assistant(e) social(e), etc.)
- 9. Diplôme de niveau Bac+3 ou Bac+4 (Licence, Licence pro., diplôme d'infirmier(e) depuis 2012, Maîtrise, Master 1, etc)
- 10. Diplôme de niveau Bac+5 (Master, DEA, DESS, grandes écoles)
- 11. Doctorat de santé (médecine, pharmacie, dentaire)
- 12. Doctorat hors santé

SI G4 = 1, 2 OU 3

SIMPLE

G3NEW

Quel est votre niveau d'études ?

Citer - Une seule réponse possible

- 1. Vous n'avez jamais fait d'études
- 2. Ecole primaire (y compris certificat d'études primaires)
- 3. 6ème à 4ème (collège)
- 4. 3ème (collège)
- 5. Première, deuxième ou dernière année de CAP-BEP ou d'une formation équivalente
- 6. Seconde ou première ou terminale de lycée
- 7. (Vous avez fait des études mais vous ne savez pas jusqu'à quel niveau)

A TOUS

SIMPLE

G6

Vivez-vous en couple ?

- 1. Oui
- 2. Non

SIMPLE

G5

Etes-vous …

ENQ : citer - Une seule réponse possible

- 1. Marié(e)
- 2. Pacsé(e)
- 3. En concubinage ou union libre
- 4. Veuf(ve)
- 5. Divorcé(e)
- 6. Célibataire → **ne pas poser si G6=1**

A TOUS

SIMPLE

G7

Dans quel pays êtes-vous né(e) ?

Saisissez au moins les 3 premières lettres du pays et choisissez votre pays de naissance parmi les pays qui vous seront proposés automatiquement.

- 1. Afrique du Sud
- 2. Albanie
- 3. Algérie
- 4. Allemagne
- 5. Andorre
- 6. Angola
- 7. Antigua-et-Barbuda
- 8. Arabie Saoudite
- 9. Argentine
- 10. Arménie
- 11. Australie
- 12. Autriche
- 13. Azerbaïdjan
- 14. Bahamas
- 15. Bahreïn

- 16. Bangladesh
- 17. Barbade
- 18. Belgique
- 19. Belize
- 20. Bénin
- 21. Bhoutan
- 22. Biélorussie
- 23. Birmanie
- 24. Bolivie
- 25. Bosnie-Herzégovine
- 26. Botswana
- 27. Brésil
- 28. Brunei
- 29. Bulgarie
- 30. Burkina Faso
- 31. Burundi
- 32. Cambodge
- 33. Cameroun
- 34. Canada
- 35. Cap-Vert
- 36. Chili
- 37. Chine
- 38. Chypre
- 39. Colombie
- 40. Comores
- 41. Corée du Nord
- 42. Corée du Sud
- 43. Costa Rica
- 44. Côte d’Ivoire
- 45. Croatie
- 46. Cuba
- 47. Danemark
- 48. Djibouti
- 49. Dominique
- 50. Égypte
- 51. Émirats arabes unis
- 52. Équateur
- 53. Érythrée
- 54. Espagne
- 55. Estonie
- 56. Eswatini
- 57. États-Unis
- 58. Éthiopie
- 59. Fidji
- 60. Finlande
- 61. France
- 62. Gabon
- 63. Gambie

© IPSOS - CONFIDENTIAL

- ❍ 64. Géorgie
- ❍ 65. Ghana
- ❍ 66. Grèce
- ❍ 67. Grenade
- ❍ 68. Guatemala
- ❍ 69. Guinée
- ❍ 70. Guinée équatoriale
- ❍ 71. Guinée-Bissau
- ❍ 72. Guyana
- ❍ 73. Haïti
- ❍ 74. Honduras
- ❍ 75. Hongrie
- ❍ 76. Îles Cook
- ❍ 77. Îles Marshall
- ❍ 78. Inde
- ❍ 79. Indonésie
- ❍ 80. Irak
- ❍ 81. Iran
- ❍ 82. Irlande
- ❍ 83. Islande
- ❍ 84. Israël
- ❍ 85. Italie
- ❍ 86. Jamaïque
- ❍ 87. Japon
- ❍ 88. Jordanie
- ❍ 89. Kazakhstan
- ❍ 90. Kenya
- ❍ 91. Kirghizistan
- ❍ 92. Kiribati
- ❍ 93. Koweït
- ❍ 94. Laos
- ❍ 95. Lesotho
- ❍ 96. Lettonie
- ❍ 100. Liban
- ❍ 101. Liberia
- ❍ 102. Libye
- ❍ 103. Liechtenstein
- ❍ 104. Lituanie
- ❍ 105. Luxembourg
- ❍ 106. Macédoine
- ❍ 107. Madagascar
- ❍ 108. Malaisie
- ❍ 109. Malawi
- ❍ 110. Maldives
- ❍ 111. Mali
- ❍ 112. Malte
- ❍ 113. Maroc
- ❍ 114. Maurice

© IPSOS - CONFIDENTIAL

- 115. Mauritanie
- 116. Mexique
- 117. Micronésie
- 118. Moldavie
- 119. Monaco
- 120. Mongolie
- 121. Monténégro
- 122. Mozambique
- 123. Namibie
- 124. Nauru
- 125. Népal
- 126. Nicaragua
- 127. Niger
- 128. Nigeria
- 129. Niue
- 130. Norvège
- 131. Nouvelle-Zélande
- 132. Oman
- 133. Ouganda
- 134. Ouzbékistan
- 135. Pakistan
- 136. Palaos
- 137. Palestine
- 138. Panama
- 139. Papouasie-Nouvelle-Guinée
- 140. Paraguay
- 141. Pays-Bas
- 142. Pérou
- 143. Philippines
- 144. Pologne
- 145. Portugal
- 146. Qatar
- 147. République centrafricaine
- 148. République démocratique du Congo
- 149. République Dominicaine
- 150. République du Congo
- 151. République tchèque
- 152. Roumanie
- 153. Royaume-Uni
- 154. Russie
- 155. Rwanda
- 156. Sainte-Lucie
- 157. Saint-Kitts-et-Nevis
- 158. Saint-Marin
- 159. Saint-Vincent-et-les-Grenadines
- 160. Salomon
- 161. Salvador
- 162. Samoa

- 163. São Tomé-et-Principe
- 164. Sénégal
- 165. Serbie
- 166. Seychelles
- 167. Sierra Leone
- 168. Singapour
- 169. Slovaquie
- 170. Slovénie
- 171. Somalie
- 172. Soudan
- 173. Soudan du Sud
- 174. Sri Lanka
- 175. Suède
- 176. Suisse
- 177. Suriname
- 178. Syrie
- 179. Tadjikistan
- 180. Tanzanie
- 181. Tchad
- 182. Thaïlande
- 183. Timor oriental
- 184. Togo
- 185. Tonga
- 186. Trinité-et-Tobago
- 187. Tunisie
- 188. Turkménistan
- 189. Turquie
- 190. Tuvalu
- 191. Ukraine
- 192. Uruguay
- 193. Vanuatu
- 194. Vatican
- 195. Venezuela
- 196. Viêt Nam
- 197. Yémen
- 198. Zambie
- 199. Zimbabwe
- 200. Autre pays
- 999. (PAYS NON TROUVE)

SI G7 = 61

SIMPLE

G7BIS

Vous êtes né en France et plus précisément ?

- 1. En métropole
- 2. A La Réunion
- 3. A Mayotte

© IPSOS - CONFIDENTIAL

❍ 4. Ailleurs en outre-mer

A TOUS

NUMERIC

G9

Combien de personnes vivent dans votre domicile y compris vous-même ?

PROG : REPONSE NUMERIQUE AVEC BORNE MINIMUM A 1

CONTROLE DE COHERENCE : SI G6=1 ET G9=1, METTRE UN MESSAGE BLOQUANT « VOUS AVEZ DECLARE VIVRE AVEC UNE AUTRE PERSONNE DANS LE FOYER. MERCI DE CORRIGER LA REPONSE »

~~*SI (G9 >1 ET G5 = 2 OU 3) OU (G9 >2 ET G5 = 1)*~~

~~NUMERIQUE~~

~~G12NEW~~

~~**Parmi ces personnes combien y a t-il d'enfants, que ce soient les vôtres ou non, y compris les enfants en garde alternée ?**~~

~~*ENQ : citer - Une seule réponse possible*~~

~~*CONTROLE DE COHERENCE : Si G6=1 et G9=2 ET G12NEW=>1, mettre un message bloquant* « VOUS AVEZ DECLARE VIVRE AVEC UNE AUTRE PERSONNE DANS LE FOYER ET AVEZ INDIQUE UN NOMBRE INSUFFISANT DE PERSONNES VIVANT DANS LE FOYER Y COMPRIS VOUS MEME. MERCI DE CORRIGER LA REPONSE »~~

❍ ~~1. OUVRIR CHAMP NUM MAX=G9-1~~
❍ ~~2. Aucun~~

~~*SI G12NEW = 1*~~

~~SIMPLE~~

~~G12.1NEW~~

~~**Quel âge a t-il ?**~~

~~*Ne pas citer*~~

❍ ~~1. Moins d'1 an~~
❍ ~~2. 1 à 2 ans~~
❍ ~~3. 3 à 5 ans~~
❍ ~~4. 6 à 10 ans~~
❍ ~~5. 11 à 14 ans~~
❍ ~~6. 15 à 18 ans~~
❍ ~~7. Plus de 18 ans~~

~~*SI G12NEW = 1 ET ITEM 1 >1*~~

~~NUMERIC~~

~~G12.2NEW~~

~~**Combien ont :**~~

~~*Enq : Saisir 0 lorsqu'il n'y a pas d'enfant de cet âge dans le foyer"*~~

~~PROG : TEST DE COHERENCE : LA SOMME DE TOUTES LES CASES DOIT ETRE > 1~~

~~SI SOMME = 1, AFFICHER MESSAGE SUIVANT : « VOUS AVEZ INDIQUE PLUSIEURS ENFANTS VIVANT DANS VOTRE DOMICILE, VEUILLEZ CORRIGER VOTRE REPONSE »~~

~~Moins d'1an :~~
~~1 à 2 ans :~~

Commenté [I1]: Erreur filtre : SI (G9 > 1 ET G6 = 2) OU (G9 > 2 ET G6 = 1)
Les réponses aux questions G12NEW, G12.1NEW, G12.2NEW, G12.3NEW et G12.4NEW sont inexploitables

© IPSOS - CONFIDENTIAL

- ~~3 à 5 ans :~~
- ~~6 à 10 ans :~~
- ~~11 à 14 ans :~~
- ~~15 à 18 ans :~~
- ~~Plus de 18 ans :~~

~~SI G12NEW = 1 ET G12.1NEW=1 OU 2 OU 3 OU 4 OU 5 OU 6~~

~~SIMPLE~~

~~**G12.3NEW**~~

~~**Au cours des douze derniers mois, l'enfant qui vit avec vous a-t-il fait, même occasionnellement y compris pendant les vacances, des activités physiques et sportives (en dehors des cours obligatoires d'éducation physique et sportive à l'école) ?**~~

~~Cela peut être les bébés nageurs pour un enfant tout jeune.~~

- ~~1. Oui~~
- ~~2. Non~~

~~SI G12NEW >= 2~~

~~SIMPLE~~

~~**G12.4NEW**~~

~~**Au cours des douze derniers mois, les enfants qui vivent avec vous ont-ils fait, même occasionnellement y compris pendant les vacances, des activités physiques et sportives (en dehors des cours obligatoires d'éducation physique et sportive à l'école ?**~~

~~Nous parlons de l'ensemble des enfants vivant avec vous.~~

~~Cela peut être les bébés nageurs pour un enfant tout jeune.~~

- ~~1. Oui tous~~
- ~~2. Oui une partie~~
- ~~3. Non~~

A TOUS

SIMPLE

G13NEW

Concernant votre rapport à l'emploi. Etes-vous … ?

Si vous êtes dans plusieurs situations à la fois, merci de répondre en décrivant la situation que vous considérez comme principale actuellement.

Citer - Une seule réponse possible

- 2. Vous travaillez (y compris en apprentissage ou professionnalisation)
- 3. Au foyer, en dehors d'un congé parental
- 4. En congé parental
- 5. Retraité(e) ayant travaillé
- 6. Retraité(e) n'ayant jamais travaillé
- 1. Elève/étudiant(e)
- 7. Au chômage, en recherche du premier emploi
- 8. Au chômage, en recherche d'emploi
- 9. Malade de longue durée ou invalide
- 10. Autre → **PROG : AFFICHER** « Veuillez vérifier que votre situation ne correspond à aucun des 9 items précédents proposés. »

SI G13NEW = 2 OU 3 OU 4 OU 5 OU 8 OU 9

SIMPLE

G15

Si G13NEW=2, AFFICHER < Etes-vous > / SI G13NEW = *3 OU 4 OU 5 OU 8 OU 9*, AFFICHER < Etiez-vous > ?

ENQ : citer - Une seule réponse possible

- 1. Indépendant.e
- 2. Salarié.e de votre propre entreprise, gérant mandataire, PDG
- 3. Salarié.e
- 4. Fonctionnaire
- 5. Je n'ai jamais travaillé ***[Ne pas afficher si G13NEW=2 ou 5 / SI G15 = 5 => Ne pas poser G15TER, ni G16, ni G16_1]***

SI G13NEW = 2 OU 3 OU 4 OU 5 OU 8 OU 9

SIMPLE

G15TER

« Dans votre emploi principal, SI G13NEW=2, AFFICHER < êtes-vous > SI G13NEW=3 OU 4 OU 5 OU 8 OU 9, AFFICHER < étiez-vous > ?

PROG : A AFFICHER DANS CET ORDRE

Une seule réponse possible

- 1. Manœuvre ou ouvrier.ière spécialisé.e
- 2. Ouvrier.ère qualifié.e ou ouvrier hautement qualifié ou technicien.ne d'atelier
- 3. Technicien.ne
- 4. Personnels de catégorie B ou assimilé
- 5. Agent de maîtrise, maîtrise administrative ou commerciale, VRP (non-cadre)
- 6. Personnel de catégorie A ou assimilé
- 7. Ingénieur.e ou cadre (à l'exception des directeurs généraux ou de leurs ~~ses~~ adjoints directs)
- 8. Personnels de catégorie C ou assimilé
- 9. Employé.es de bureau, de commerce, personnel de service
- 10. Directeur.trice général.e ou adjoint.e direct.e au directeur.trice

SI G13_NEW = 2 OU 3 OU 4 OU 5 OU 8 OU 9 / SI G13NEW = 2 OU 3 OU 4 OU 5 OU 8 OU 9

OUVERTE

G16

***SI G13NEW = 2, AFFICHER* < Pouvez-vous indiquer l'intitulé exact de la profession que vous exercez > *SI G13NEW = 3 OU 4 OU 5 OU 8 AFFICHER* < Pouvez-vous indiquer l'intitulé exact de la profession que vous avez exercée > ?**

Merci d'écrire votre métier au masculin.

SAISIR LES PREMIERES LETTRES DE VOTRE PROFESSION – PUIS LA SELECTIONNER DANS LA LISTE QUI VA APPARAITRE (CELA PEUT PRENDRE QUELQUES SECONDES)

Pour valider votre choix, cliquer sur suivant (Flèche de droite)

ENQ : Si vous ne la trouvez pas, cliquez sur « Vous n'avez pas trouvé dans la liste

Si vous avez plusieurs emplois, il s'agit de l'emploi qui vous occupe le plus de temps, ou en cas d'égalité, celui qui vous procure le plus de revenus.

SI G13NEW = 2 OU 3 OU 4 OU 5 OU 8 OU 9

SIMPLE

G16.1

SI G13NEW=2, AFFICHER **< Vous arrive-t-il >** ***SI G13NEW = 3 OU 4 OU 5 OU 8 OU 9, AFFICHER*** **<Vous est-il arrivé > de travailler en horaires décalés (par exemple entre 20h et minuit, entre minuit et 5h du matin, ou alors le samedi ou le dimanche) ?**

ENQ : citer - Une seule réponse possible

- 1. Tout le temps
- 2. Souvent
- 3. Quelquefois
- 4. Jamais

SI G13NEW = 1 OU 7

SIMPLE

G16NEW

Pouvez-vous indiquer la profession que votre parent (beau-parent ou tuteur) exerce ou a exercée ?
Nous allons nous intéresser à la profession du parent (beau-parent ou tuteur) qui gagne le plus d'argent pour votre famille.

Une seule réponse possible

- 1. Agriculteur.trice exploitant.e
- 2. Artisan, commerçant.e ou assimilé.e, chef.fe d'entreprise de 10 salariés ou plus
- 3. Cadre, profession libérale, profession intellectuelle supérieure
- 4. Profession intermédiaire, professeur.e des écoles, instituteur.trice, technicien.ne, contremaître, agent de maîtrise, clergé
- 5. Employé.e, agent de service de la fonction publique, personnel de service
- 6. Ouvrier.ère, chauffeur.euse
- 7. Inactif.ve n'ayant jamais travaillé
- 8. (Je ne sais pas) ***PROG : En CATI : afficher : [Ne sait pas]***

SI (G6 = 1) OU (G5 = 4 OU 5)

SIMPLE

G17

Diriez-vous que votre conjoint / conjointe SI G5 = 4 ou 5 AFFICHER < était > SI G6=1 AFFICHER < est > :

ENQ : citer - Une seule réponse possible

- 1. Pas sportif(ve)
- 2. Peu sportif(ve)
- 3. Assez sportif(ve)
- 4. Très sportif(ve)
- 5. (Je ne sais pas)

A TOUS

SIMPLE

G17A

SI AGE ≥ 20, AFFICHER **< Lorsque vous étiez enfant ou adolescent.e, diriez-vous que votre mère était … >** ***SI AGE < 20, AFFICHER*** **< Diriez-vous que votre mère est … >**

ENQ : citer - Une seule réponse possible

- 1. Pas sportive
- 2. Peu sportive
- 3. Assez sportive
- 4. Très sportive

- 5. (Je ne sais pas)

SI C0>0 ET G17A=2 OU 3 OU 4

SIMPLE

G17A.BIS

SI AGE ≥ 20, AFFICHER **Lorsque vous étiez enfant ou adolescent.e, pratiquiez-vous une activité physique et sportive commune avec votre mère ?** ***SI AGE < 20, AFFICHER*** **Pratiquez-vous une activité commune avec votre mère ?**

- 1. Oui
- 2. Non
- 3. (Je ne sais pas)

SI G17A.BIS=1

SIMPLE

G17A.BIS2

SI AGE ≥ 20, AFFICHER **Quelle était l'activité physique et sportive principale commune avec votre mère** ***SI AGE < 20, AFFICHER*** **Quelle est l'activité physique et sportive principale commune avec votre mère ?**

Enq : une seule réponse possible

- **Prog : reprendre liste B1 : réponse spontanée**
- **(Ne sait pas)**

A TOUS

SIMPLE

G17B

SI AGE ≥ 20, AFFICHER **< Lorsque vous étiez enfant ou adolescent.e, diriez-vous que votre père était ... > /** ***SI AGE < 20, AFFICHER*** **< Diriez-vous que votre père est ... >**

ENQ : citer - Une seule réponse possible

- 1. Pas sportif
- 2. Peu sportif
- 3. Assez sportif
- 4. Très sportif
- 5. (Je ne sais pas)

SI C0>0 ET G17B=2 OU 3 OU 4

SIMPLE

G17B.BIS

SI AGE ≥ 20, AFFICHER **Lorsque vous étiez enfant ou adolescent.e, pratiquiez-vous une activité physique et sportive commune avec votre père ?** ***SI AGE < 20, AFFICHER*** **Pratiquez-vous une activité commune avec votre père ?**

- 1. Oui
- 2. Non
- 3. (Je ne sais pas)

SI G17B.BIS=1

SIMPLE

G17B.BIS2

SI AGE ≥ 20, AFFICHER **Quelle était l'activité physique et sportive principale commune avec votre père** ***SI AGE < 20, AFFICHER*** **Quelle est l'activité physique et sportive principale commune avec votre père ?**

Enq : une seule réponse possible

- **Prog : reprendre liste B1 : réponse spontanée**
- **(Ne sait pas)**

A TOUS

SIMPLE

G18.3

Actuellement, vous diriez qu'au sein de votre ménage / famille, financièrement ...

ENQ : citer - Une seule réponse possible

- 1. Vous êtes à l'aise
- 2. Ça va
- 3. C'est juste
- 4. Vous y arrivez difficilement
- 5. Vous ne pouvez pas y arriver sans faire de dettes (ou avoir recours au crédit à la consommation)

A TOUS

SIMPLE

G29

Votre adresse est-elle bien la suivante ?

PROG : AFFICHER L'ADRESSE EN INFO

Adresse : ADRESSE
Complément d'adresse : COMPLEMENT_ADRESSE
Code postal : CODE_POSTAL
Ville : LIBELLE_COMMUNE

- 1. Oui
- 2. Non
- 3. Refus

SI G29 = 2 OU ADRESSE POSTALE INCONNUE

OUVERTE

G29A

Quelle est votre adresse ?

- Adresse :
- Complément d'adresse :
- Code postal :
- Ville :

- Je ne souhaite pas communiquer mon adresse

TOP FIN MODULE G

Conclusion 1er écran

CATI : **Le questionnaire est à présent terminé. + [Bouton « Valider les réponses »]**

Conclusion 2ème écran

[Fin de l'entretien]. La Région Réunion et l'Université de La Réunion vous remercient pour votre participation et du temps que vous avez bien voulu accorder à cette étude.

Enq : veuillez repasser sur la partie contact pour valider définitivement votre entretien

TOP FIN QUESTIONNAIRE
