# Supplementary material for: Determinants of adult sedentary behavior and physical inactivity for the primary prevention of diabetes in historically disadvantaged communities: A representative cross-sectional population-based study from Reunion Island
Source: PLoS One. 2024 Aug 13;19(8):e0308650. doi: 10.1371/journal.pone.0308650 (PMC11321555; doi:10.1371/journal.pone.0308650)
Supplement: S2 Appendix — This file gives information on composite variables: the Positive perception of the personal and parental history of PSA, and the Physical environment indicators characterizing the residential environment. (DOCX) [file pone.0308650.s002.docx]

**S2 Appendix. Data management and rationale**

| Positive perception of the personal and parental history of PSA | Physical environment indicators characterizing the residential environment |
| --- | --- |
| For participants practicing PSA at the time of the study, positive perception of the personal and parental history of PSA was defined by a response above or equal to the median value (7) on a scale of 1 (not at all happy) to 10 (totally happy), and having indicated that they had the perception of either their mother and/or father having practiced sports very often or relatively often during the participant’s childhood or adolescence. For those not practicing PSA at the time of the study, the same calculation was performed assuming a score inferior to 7. A positive perception of the personal and parental history of PSA (Yes/No) determined at the time of the study could both explain sedentary behavior and physical inactivity, and the socio-economic characteristics of the person interviewed (diplomas obtained, professional status and socio-professional category among others).While the former expected link is explicit ^a^, the latter seems more difficult to understand because it implies taking into consideration the personal and parental history of PSA corresponding to the extent to which a person is accompanied by his or her peers as well as personal achievement and self-esteem ^a^. These factors could influence the educational and professional track record of the person concerned. For example, a person living in a family that practices sports in a regular manner during his or her childhood would be likely to develop capacities of adaptation, coping or resilience leading them to be more easily and more likely situated at the top of the social ladder than other individuals who lived in a context where sports were never or seldom practiced. This rationale led to consider a positive perception of the personal and parental history of PSA (Yes/No) as a confounding factor regarding the relationship between sedentary behavior or physical inactivity and individual socio-economic status. | Geo-treatment was applied using QGIS software version 3.10 (Coruna) on the basis of IRIS in their 2019 version (version referred to as Contour-IRIS®) corresponding to 344 entities on Reunion Island. The rate of artificial cover of the ground of the IRIS of residence (buildings, parking spaces and roads) was an indicator based on the occupied ground layer in 2021 ^b^ carried out by the Cirad (French agricultural research center for international development). It was obtained from a SPOT6 image with a resolution of 1.5 meters and a Sentinel-2 temporal series. The category of occupation of the ground used (class 4) was obtained from the first level of this product. The total precision of the layer achieved a value of 98% (high-quality) coherent over the whole of the island’s territory. From an operational point of view, category 4 of Level 1 was extracted from the complete layer through a simple attribute request. This layer was then transformed into a raster with a resolution of 2 meters to obtain a binary file (0: absence of artificial zone versus 1: presence). Extraction of this information was then carried out on the IRIS applying the *zonal statistics* function of the QGIS software by taking the number (count) of pixels of ‘presence’ per entity. The figure was then multiplied by four to obtain the number of square meters. The result was divided by the total area of the IRIS entity and multiplied by 100 to obtain the rate of artificial cover as a percentage. For statistical exploitation, this rate was dichotomized to the threshold of the median (≤36% or >36%).  The mean annual temperature of the IRIS of residence over 30 years was the result of an interpolation over the entire Reunion Island territory and was derived from measurements obtained from 73 stations of Météo-France (the French National Weather Service) and Cirad between the years 1987 and 2017 (Source: https://aware.cirad.fr/layers/geonode:temp_moy/metadata_detail). This information was extracted to establish an average by IRIS applying the *zonal statistics* function of QGIS software. For statistical purposes, the distribution of the mean annual temperature of the IRIS of residence over 30 years was divided up into tertiles (≤21.3°C, 21.4°C to 22.4°C, > 22.4°C). |

^a^ Taylor D, Bury M, Campling N, Carter S, Garfied S, Newbould J, et al. A Review of the use of the Health Belief Model (HBM), the Theory of Reasoned Action (TRA), the Theory of Planned Behaviour (TPB) and the Trans-Theoretical Model (TTM) to study and predict health related behaviour change. The School of Pharmacy, University of London; 2006.

^b^ https://dataverse.cirad.fr/dataset.xhtml?persistentId=doi:10.18167/DVN1/MR4YT9
