# Supplementary material for: Determinants of adult sedentary behavior and physical inactivity for the primary prevention of diabetes in historically disadvantaged communities: A representative cross-sectional population-based study from Reunion Island
Source: PLoS One. 2024 Aug 13;19(8):e0308650. doi: 10.1371/journal.pone.0308650 (PMC11321555; doi:10.1371/journal.pone.0308650)
Supplement: S1 Fig — (DOCX) [file pone.0308650.s003.docx]

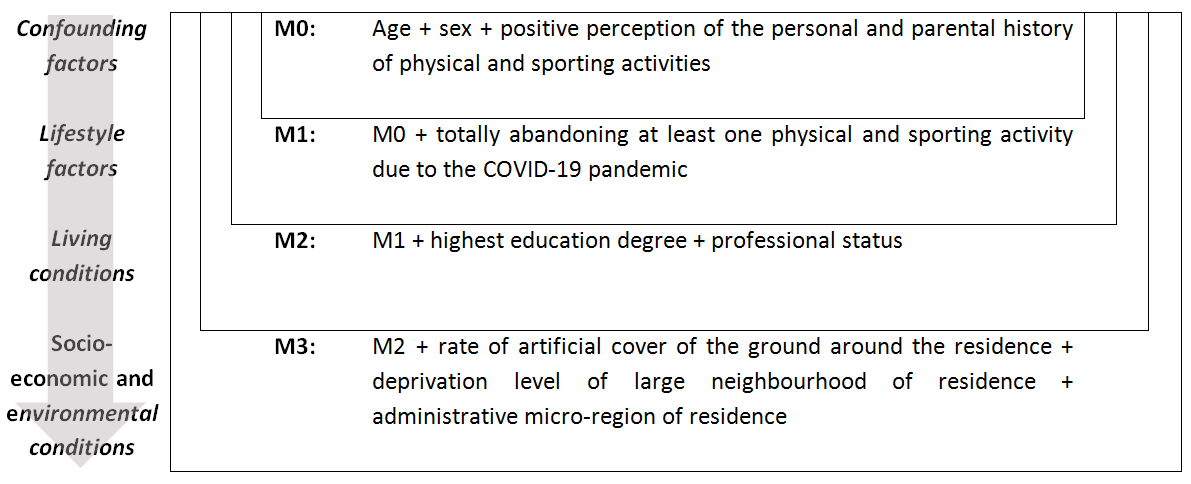


Notes: An initial pre-selection of factors was carried out according to data quality and by retaining significant variables at a threshold of p≤0.10 (based on Rao-Scott’s Khi-2 test) at the stage of bivariate analysis, which led to 16 preselected factors. Following this, pre-selection was refined at the threshold of p≤0.05 (controlling for the confounding factors) resulting in 6 of 16 factors being preselected. Finally, based on the results of this process, and the inclusion of the three confounding factors (giving 9 factors in total), nested multivariable regression models estimated on the same statistical sample were built by applying the following sequence. The basic model (M0) included the three confounding factors of age, sex and the positive perception of the personal and parental history of PSA. The objective was to control for any possible imbalance between the groups defined by the new categorical variables introduced into the subsequent M1 and M2 models. The M1 model included M0 and an individual lifestyle factor (Dahlgren and Whitehead, 2021). The M2 model included M1 and two indicators of living conditions (Dahlgren and Whitehead, 2021). Finally, the complete model (M3) was constructed based on M2 to which socio-economic and environmental factors (Dahlgren and Whitehead, 2021) concerning the residential environment were added. In total, this sequential approach made it possible to examine the change of effects associated with each of the factors of exposure following the introduction of other factors.

**S1 Fig. Nested multivariable regression models (M0, M1, M2, and M3) sequential approach**
