# Supplementary material for: Determinants of adult sedentary behavior and physical inactivity for the primary prevention of diabetes in historically disadvantaged communities: A representative cross-sectional population-based study from Reunion Island
Source: PLoS One. 2024 Aug 13;19(8):e0308650. doi: 10.1371/journal.pone.0308650 (PMC11321555; doi:10.1371/journal.pone.0308650)
Supplement: S1 Table — (DOCX) [file pone.0308650.s004.docx]

**S1 Table.** **Individual and social factors independently associated with each of the three risk profiles of people aged 15 years and over on Reunion Island in 2021 (M1 and M2 Nested multivariable regression models using non-sedentary/active profile as reference)**

| FACTORS | Sedentary/Inactive profile | | Sedentary/Active profile | | Non-sedentary/Inactive profile | |
| --- | --- | --- | --- | --- | --- | --- |
|  | **aOR [95% CI]** | **p** | **aOR [95% CI]** | **p** | **aOR [95% CI]** | **p** |
| Model 1 (M1) | | | | |  |  |
| At least one practice of PSA being abandoned because of the COVID-19 pandemic |  |  |  |  |  |  |
| No/not concerned | 1 | - | 1 | - | 1 | - |
| Yes | 2.26 [1.49 to 3.41] | <.001 | 1.82 [1.34 to 2.47] | <0.001 | 1.62 [0.90 to 2.91] | 0.106 |
| Model 2 (M2) | | | | |  |  |
| At least one practice of PSA being abandoned because of the COVID-19 pandemic |  |  |  |  |  |  |
| No/not concerned | 1 | - | 1 | - | 1 | - |
| Yes | 2.20 [1.45 to 3.32] | <.001 | 1.75 [1.28 to 2.38] | <0.001 | 1.64 [0.91 to 2.97] | 0.102 |
| Education degree (highest) |  |  |  |  |  |  |
| No diploma or primary level of education | 1 | - | 1 | - | 1 | - |
| Lower high-school education or professional certificate | 0.83 [0.47 to 1.47] | 0.517 | 1.08 [0.71 to 1.63] | 0.726 | 0.86 [0.45 to 1.66] | 0.652 |
| Final secondary school diploma or above | 1.64 [0.98 ; 2.73] | 0.059 | 1.77 [1.19 to 2.62] | 0.005 | 0.58 [0.25 to 1.35] | 0.204 |
| Professional status |  |  |  |  |  |  |
| Other^a^ | 1 | - | 1 | - | 1 | - |
| Employed | 1.32 [0.68 to 2.56] | 0.405 | 1.22 [0.75 to 2.00] | 0.425 | 0.64 [0.29 to 1.43] | 0.275 |
| Unemployed | 2.12 [0.97 to 4.64] | 0.058 | 1.36 [0.77 to 2.39] | 0.286 | 0.74 [0.34 to 1.64] | 0.463 |
| Student | 3.85 [1.49 to 9.95] | 0.005 | 4.43 [2.18 to 8.99] | <0.001 | 0.85 [0.15 to 4.71] | 0.850 |
| Retired | 1.04 [0.41 to 2.65] | 0.943 | 1.16 [0.47 to 2.83] | 0.751 | 0.49 [0.18 to 1.34] | 0.162 |

aOR [95% CI]: adjusted odds ratio [95% confidence interval]. PSA: physical and sporting activities. All regression models were adjusted based on age range (15 to 29/30 to 44/45 to 59/60 years+), sex (female/male) and positive perception of the personal and parental history of PSA (yes/no).

^a^ Persons staying at home and not on parental leave, long-term sick leave, disabled persons and categories of inactive persons (other than unemployed, pupils, tertiary students and retired persons).
