# Supplementary material for: Determinants of adult sedentary behavior and physical inactivity for the primary prevention of diabetes in historically disadvantaged communities: A representative cross-sectional population-based study from Reunion Island
Source: PLoS One. 2024 Aug 13;19(8):e0308650. doi: 10.1371/journal.pone.0308650 (PMC11321555; doi:10.1371/journal.pone.0308650)
Supplement: S2 Table — (DOCX) [file pone.0308650.s005.docx]

**S2 Table. Individual and socio-environmental factors associated with the three risk profiles of people aged 15 and over on Reunion Island in 2021 (Non-adjusted regression models using non-sedentary/active profile as reference)**

|  | Sedentary/Inactive profile |  | Sedentary/Active profile |  | Non-sedentary/Inactive profile |  |
| --- | --- | --- | --- | --- | --- | --- |
| FACTORS | **Crude OR [95% CI]** | **p** | **Crude OR [95% CI]** | **p** | **Crude OR [95% CI]** | **p** |
| Age range |  |  |  |  |  |  |
| 45 to 59 years | 1 | - | 1 | - | 1 | - |
| 15 to 29 years | 2.26 [1.39 to 3.69] | 0.001 | 2.71 [1.91 to 3.85] | <0.001 | 0.43 [0.17 to 1.12] | 0.083 |
| 30 to 44 years | 1.01 [0.64 to 1.60] | 0.973 | 1.02 [0.74 to 1.40] | 0.914 | 1.08 [0.56 to 2.05] | 0.825 |
| 60 years and over | 1.24 [0.72 to 2.13] | 0.445 | 0.82 [0.55 to 1.22] | 0.329 | 1.50 [0.80 to 2.81] | 0.203 |
| Sex |  |  |  |  |  |  |
| Male | 1 | - | 1 | - | 1 | - |
| Female | 1.02 [0.71 to 1.47] | 0.905 | 1.02 [0.80 to 1.31] | 0.854 | 1.88 [1.13 to 3.12] | 0.015 |
| Positive perception of the personal and parental history of PSA |  |  |  |  |  |  |
| Yes | 1 | - | 1 | - | 1 | - |
| No | 2.35 [1.43 to 3.87] | 0.001 | 1.00 [0.75 to 1.33] | 0.988 | 14.69 [4.39 to 49.18] | <0.001 |
| At least one practice of PSA being abandoned because of the COVID-19 pandemic |  |  |  |  |  |  |
| No/not concerned | 1 | - | 1 | - | 1 | - |
| Yes | 2.28 [1.51 to 3.45] | <0.001 | 1.81 [1.34 to 2.45] | <0.001 | 1.79 [1.00 to 3.21] | 0.050 |
| Perception of general state of health |  |  |  |  |  |  |
| Very good to good | 1 | - | 1 | - | 1 | - |
| Quite good/don’t know (n=3) | 1.07 [0.72 to 1.58] | 0.738 | 0.76 [0.57 to 1.02] | 0.066 | 1.52 [0.89 to 2.58] | 0.123 |
| Bad to very bad | 2.19 [1.10 to 4.35] | 0.026 | 0.89 [0.49 to 1.60] | 0.692 | 1.61 [0.69 to 3.75] | 0.275 |
| Disability |  |  |  |  |  |  |
| No | 1 | - | 1 | - | 1 | - |
| Yes | 1.59 [0.82 to 3.06] | 0.170 | 0.74 [0.43 to 1.30] | 0.300 | 1.93 [0.84 to 4.45] | 0.123 |
| Body mass index (missing data=45^a^) |  |  |  |  |  |  |
| Normal weight (18.5 to 24.9 kg/m²) | 1 | - | 1 | - | 1 | - |
| Underweight (<18.5 kg/m²) | 1.86 [0.81 to 4.29] | 0.146 | 1.36 [0.77 to 2.39] | 0.287 | 0.93 [0.31 to 2.83] | 0.903 |
| Overweight (25.0 to 29.9 kg/m²) | 0.84 [0.56 to 1.26] | 0.410 | 0.74 [0.55 to 0.98] | 0.035 | 1.66 [0.97 to 2.85] | 0.064 |
| Obese (≥30.0 kg/m²) | 1.17 [0.63 to 2.18] | 0.617 | 1.41 [0.95 to 2.08] | 0.089 | 1.08 [0.46 to 2.56] | 0.857 |
| Language spoken in the home at the age of five |  |  |  |  |  |  |
| Only Creole | 1 | - | 1 | - | 1 | - |
| Only French | 1.36 [0.78 to 2.35] | 0.279 | 1.73 [1.22 to 2.47] | 0.002 | 0.52 [0.25 to 1.09] | 0.083 |
| French and Creole | 1.33 [0.87 to 2.02] | 0.191 | 1.55 [1.16 to 2.08] | 0.003 | 0.56 [0.32 to 0.99] | 0.044 |
| Other | 1.06 [0.50 to 2.24] | 0.884 | 1.53 [0.94 to 2.48] | 0.085 | 0.33 [0.12 to 0.93] | 0.035 |
| Living situation |  |  |  |  |  |  |
| Living in a couple with other persons | 1 | - | 1 | - | 1 | - |
| Living alone | 1.95 [1.13 to 3.38] | 0.017 | 1.22 [0.81 to 1.84] | 0.352 | 1.27 [0.60 to 2.69] | 0.538 |
| Single living with other persons | 1.91 [1.28 to 2.85] | 0.002 | 2.04 [1.55 to 2.70] | <0.001 | 1.02 [0.59 to 1.77] | 0.940 |
| Perception of financial difficulties of the household |  |  |  |  |  |  |
| Just breaking even or in difficulty | 1 | - | 1 | - | 1 | - |
| Good or comfortably off | 1.16 [0.81 to 1.67] | 0.427 | 1.41 [1.09 to 1.83] | 0.008 | 1.18 [0.72 to 1.93] | 0.516 |
| Education degree (highest) |  |  |  |  |  |  |
| No diploma or primary level of education | 1 | - | 1 | - | 1 | - |
| Lower high-school education or professional certificate | 0.89 [0.51 to 1.54] | 0.665 | 1.44 [0.97 to 2.14] | 0.071 | 0.74 [0.42 to 1.32] | 0.310 |
| Final secondary school diploma or above | 1.48 [0.89 to 2.45] | 0.127 | 2.09 [1.44 to 3.04] | <0.001 | 0.41 [0.22 to 0.77] | 0.006 |
| Professional status |  |  |  |  |  |  |
| Other ^b^ | 1 | - | 1 | - | 1 | - |
| Employed | 1.42 [0.77 to 2.59] | 0.258 | 1.42 [0.87 to 2.33] | 0.162 | 0.45 [0.22 to 0.89] | 0.021 |
| Unemployed | 2.03 [0.98 to 4.19] | 0.056 | 1.39 [0.80 to 2.42] | 0.247 | 0.63 [0.28 to 1.41] | 0.259 |
| Student | 4.57 [2.10 to 9.96] | <0.001 | 6.55 [3.48 to 12.32] | <0.001 | 0.26 [0.06 to 1.11] | 0.069 |
| Retired | 1.41 [0.68 to 2.94] | 0.358 | 1.03 [0.58 to 1.85] | 0.919 | 0.63 [0.29 to 1.37] | 0.246 |
| Rate of artificial cover of the ground of the IRIS of residence |  |  |  |  |  |  |
| ≤36% | 1 | - | 1 | - | 1 | - |
| >36% | 1.28 [0.89 to 1.84] | 0.183 | 1.45 [1.13 to 1.87] | 0.004 | 1.47 [0.90 to 2.41] | 0.120 |
| Mean annual temperature of the IRIS of residence over 30 years |  |  |  |  |  |  |
| ≤21.3°C | 1 | - | 1 | - | 1 | - |
| 21.3°C to 22.4°C | 1.18 [0.75 to 1.85] | 0.485 | 1.47 [1.06 to 2.03] | 0.020 | 1.01 [0.55 to 1.84] | 0.972 |
| >22.4°C | 1.50 [0.96 to 2.33] | 0.074 | 1.57 [1.14 to 2.16] | 0.006 | 1.01 [0.55 to 1.83] | 0.987 |
| Deprivation level of the large neighborhood of residence (1: most deprived to 5: least deprived)^c^ |  |  |  |  |  |  |
| 1 | 1 | - | 1 | - | 1 | - |
| 2 | 0.95 [0.36 to 2.50] | 0.916 | 0.48 [0.30 to 0.77] | 0.002 | 0.92 [0.38 to 2.22] | 0.851 |
| 3 | 2.09 [0.82 to 5.34] | 0.124 | 0.72 [0.46 to 1.14] | 0.165 | 1.24 [0.50 to 3.09] | 0.639 |
| 4 | 1.41 [0.57 to 3.51] | 0.463 | 0.55 [0.36 to 0.83] | 0.004 | 0.60 [0.25 to 1.44] | 0.252 |
| 5 | 1.69 [0.66 to 4.34] | 0.273 | 0.82 [0.53 to 1.28] | 0.389 | 0.77 [0.30 to 1.97] | 0.586 |
| Administrative micro-region of residence |  |  |  |  |  |  |
| Southern region | 1 | - | 1 | - | 1 | - |
| Eastern region | 0.86 [0.52 to 1.45] | 0.576 | 1.02 [0.72 to 1.45] | 0.898 | 1.24 [0.67 to 2.29] | 0.498 |
| Northern region | 1.64 [1.01 to 2.68] | 0.046 | 1.58 [1.13 to 2.21] | 0.008 | 1.06 [0.54 to 2.10] | 0.858 |
| Western region | 1.24 [0.76 to 2.05] | 0.390 | 1.15 [0.81 to 1.61] | 0.435 | 0.72 [0.36 to 1.44] | 0.356 |

OR [95% CI]: odds ratio [95% confidence interval]. PSA: physical and sporting activities. IRIS: aggregated units for the statistical information.

^a^ Missing data due to undeclared height or weight.

^b^ Persons staying at home and not on parental leave, long-term sick leave, disabled persons and categories of inactive persons (other than unemployed, pupils, tertiary students and retired persons).

^c^ Regional data ([24])
