# Supplementary material for: Determinants of adult sedentary behavior and physical inactivity for the primary prevention of diabetes in historically disadvantaged communities: A representative cross-sectional population-based study from Reunion Island
Source: PLoS One. 2024 Aug 13;19(8):e0308650. doi: 10.1371/journal.pone.0308650 (PMC11321555; doi:10.1371/journal.pone.0308650)
Supplement: Attachment — Submitted filename: Review_PONE-D-23-43256.pdf [file pone.0308650.s006.pdf]

Manuscript Number: PONE-D-23-43256.
Title: Determinants of adult sedentary behavior for the primary pre of diabetes in historically disadvantaged communities : a representative cross-sectional study from Reunion island.
Article Type: Research article

General comment

Sedentary lifestyle is a key determinant of obesity and of its subsequent complications such as type 2 diabetes mellitus (T2D), blood hypertension, arterial stiffness, and beyond, cardiovascular ischemic diseases. Understanding the pathways between sedentary behavior risk factors, especially those that are modifiable, and T2D is therefore of paramount importance to prevent T2D and its related burden of morbimortality. In this framework, Fianu et al. have posited a holistic approach of health determinants for investigating both individual and socio-environmental (contextual) risk factors of adult sedentary behavior living in Reunion Island with the aim to pave the way for future public heath interventions in the primary prevention of T2D. The topic is of public health relevance given the global epidemic of obesity and T2D, whose incidences peak in vulnerable communities like those observed in the southwestern Indian ocean (SWIO) region. The team from which it comes seems to have mastered this type of intervention, which warrants interesting perspectives for the transformation process into future actions (Fianu et al, *Sante Publique* 2017; Fianu et al*, PLoS One* 2016; Debussche et al, *Diabetes Metabolism* 2012). The paper is thorough and well-written. It carries and spreads very interesting methodological options that it deserves publication to shed light on the intricate links between social inequalities in health (SIH), physical and sport activity (PSA) and sedentary behaviors in the setting of T2D prevention. This notably because literature still lacks well-designed population-based studies from high-risk regions, which face multiple challenges best known under the concept of epidemiological transition. However, before reaching this achievement, the authors should consider minor compulsory and minor discretionary revisions including clearer presentation of study findings, language simplifications, and discussion of strategies in analysis. With an appropriate revision, given the abovementioned, the paper could even be eligible to PLoS Global Public Health, a very promising new PLOS journal of Public Health/Global Health with a strong focus on social inequalities in health.

Specific comments

Minor compulsory revision (mandatory)

Title and abstract
1(*a*). Indicate the study's design with a commonly used term in the title or the abstract. Add "population-based" to study as it is a strength of the study.
The abstract is too narrative and should include quantitative results.
1(*b*). Page 2, line 31. Replace "territory" by department, which is more exact administratively.
Page 2, line 38. The authors must define the categories of interest of their multinomial outcome.
Page 2, lines 41 to 42. "… associated with sedentary or physical inactive behavior." The formulation makes think the outcome is composite and binary while it is four categorial.
Page 2, line 44. I am not sure a research perspective has to be placed in the abstract.

Page 2, line49 to page 3, line 50. Rephrase the last sentence of the abstract as “reducing” may apply for lifestyle risk factors but not T2D primary prevention programs.
Page 2, line 49. “may suggest.” Why “may” ? The authors should better trust themselves and write “suggest” or “may foster” or something stronger like that.

Introduction
2. Background/rationale
Explain the scientific background and rationale for the investigation being reported.
Page 3, lines 58 to 59. Rephrase the sentence to distinguish among the determinants of chronic diseases (such as T2D), risk factors from mediators. For instances, writing “that are intrinsically linked to both the development of sedentary lifestyle and excessive caloric intake through metabolic disturbances (insulin resistance).” Diabetes should not be placed at the end of the sentence as it is one of the chronic diseases to what the authors refer to.
Page 3, lines 61.”is… and is…” Two times is for the same subject, T2D. Rephrase the sentence starting with Type 2 diabetes (T2D),…[4, 5].”
Page 3, line 73 to page 4, line 74. Does the author use social inequalities for social inequalities of health? If yes, replace social inequalities by SIH to avoid the truism as it seems evident that differences in income and unfavorable living and working conditions define social inequalities.
3. Objectives
State specific objectives, including any prespecified hypotheses.
The authors should be more explicit on their hypotheses. We guess they are presented in the long sentence preceding the objectives page 4, lines 82 to 84. They could use a formulation like “Given the abovementioned blablabla, we hypothesize … .” They should also mention and define physical and sport activities as physical activity/inactivity is included in their multinomial four- category outcome. For readers not familiar to the literature about physical activity and health, it may be difficult to distinguish sedentary behavior and absence of physical activity.

Methods
4. Study design
Present key elements of study design early in the paper
5. Setting
Describe the setting, locations, and relevant dates, including periods of recruitment, exposure, follow-up, and data collection.
Page 5, line 98. Define the acronym ERPPS in the text and makes clear that the study reports this study as it is published formerly in a study report for public health stakeholders, academics and funders but not as a scientific publication.
Page 5, line 98. “was collected” is abrupt and let imagine it these are findings of a new study disconnected from the ERPPS study. Better use “had been” to let see, the data had been previously collected.
Page 5, line 101. Write “aged 15 or over” as it is really “≥” and not “>”.
Page 5, line 107. Define the acronym “Fideli” in French in italics. Acronym should be defined in French and English translations added when necessary.
Page 5, line 112. Define the categories of the level of income to see how it was categorized. It will make more intelligible the number of possible strata in the random sampling.
7. Variables
Clearly define all outcomes, exposures, predictors, potential confounders, and effect modifiers. Give diagnostic criteria, if applicable

Lifestyle behaviors outcomes. This important paragraph should be rephrased and start from the definition of the multinomial four-category outcome, followed by clearer definitions of each of its categories.
The authors clearly have focused their hypothesis and objectives on sedentary behavior and not physical activity. This paragraph should explain why physical activity was integrated in the outcome definition and note taken as a potential effect-modifier (or even as a potential confounder).
Page 6, line 134. Physical inactivity is a composite variable which combined four reported outcome variables.
Page 6, line 137. Remove "reporting" as it has been specified previously that data were self-reported.
Confounding factors. Page 7, lines 147 to 148. The sentence is confusing. How a health-related behavior like a sedentary behavior could confound sedentary behavior?
Factors of exposure. Page 7, line 158. Replace "threshold" by "categories" as we need three thresholds or cut-off to define four categories.
Page 7, line 160. I don't think language spoken in the home may be defined as a psychosocial characteristic. Please explain.
Page 7, lines 179 to 182. Define the referent category as zero category and the categorization 0 to 4.
8. Data sources/ measurement
For each variable of interest, give sources of data and details of methods of assessment (measurement). Describe comparability of assessment methods if there is more than one group.
Page 6, line 124. Which ecological data were compiled?
9. Bias
Describe any efforts to address potential sources of bias
10. Study size
Explain why this study did not require sample size pre-specification (instead of how the study size was arrived at).
11. Quantitative variables
Explain how quantitative variables were handled in the analyses. If applicable, describe which groupings were chosen and why?
12. Statistical methods
12 (*a*). Describe all statistical methods, including those used to control for confounding.
12 (*b*). Describe any methods used to examine subgroups and interactions.
It should be explained why physical activity was integrated into the multinomial four-category outcome as it precludes study physical activity as an effect-modifier or a confounder.
12 (*c*). Explain how missing data were addressed. This paragraph page 9, lines 205 to 215 is
The paragraph page 9, lines 205 to 212 is difficult to read for non-statistician readers and has little impact on the understanding the article. I would shorten it and merely explain that there were multiple imputations.
Page 9, line 200. "The working framework." Working could be replaced by operating or something like that.
Ethical considerations. Page 10, lines 234 to 235. Explain why minors 15 to 17 were not asked to provide their oral consent along with that of their parents.

Commenté [pg1]: Give information separately for cases and controls in case-control studies and, if applicable, for exposed and unexposed groups in cohort and cross-sectional studies.

Results

13. Participants
13 (a).Page 11, line 241. Remove the “with” and reformulate using a formulation like “which represented” or something like that.
14. Descriptive data
14 (a). Give characteristics of study participants (eg demographic, clinical, social) and information on exposures and potential confounders.
Page 11, lines 247 to 251. Although, Table 1 is a descriptive table, were there some comparisons between the study sample and census data. When percentages differed it could have shed light on potential selection biases? (even though in this case differences are significant due to statistical power.
Page 16, lines 273 to 274. Most participants possessed an educational degree. This could have been a source of selection bias. Explain and specify if there was or there wasn't a selection bias and how it was accounted if it had an impact on study findings.
Page 16, lines 274 to 275. Define a positive perception.
Page 16, line 281. What for students?.
14 (b). Indicate number of participants with missing data for each variable of interest
15. Outcome data
15 (c). *Cross-sectional study*—Report numbers of outcome events or summary measures

Commenté [pg2]: Give information separately for cases and controls in case-control studies and, if applicable, for exposed and unexposed groups in cohort and cross-sectional studies.

16. Main results
16 (*a*). Unadjusted estimates could be placed as supporting information(supplemental online tables). Make clear which confounders were adjusted for and why they were included.
It is inappropriate and very confusing to report a single analysis on three different tables. Tables 2 to 4 should be merged into a single table presenting the M3 final model distinguishing each outcome category (Y1 in place of M1, Y2 in place of M2, as study findings that are interpreted are only those of M3 models. Results of nested models M1 and M2 models which are not interpreted or very simply in short sentences, should be presented as supporting information and supplemental online tables.
Tables 2 to 4. With respect to the prespecified hypotheses, why not having taken “final secondary school diploma or above as referent category?
16 (*b*). Report category boundaries when continuous variables were categorized
16 (*c*). If relevant, consider translating estimates of relative risk into absolute risk for a meaningful time period
17 Other analyses
Report other analyses done—eg analyses of subgroups and interactions, and sensitivity analyses.
Page 16, lines 264 to 267. Explain why results have not been presented at least for some microregions that shaped or contrasted the overall study findings.
Page 23, line 323. Present crude unadjusted analyses like the primary analysis: M1 with Y1,Y2 and Y3 in columns, M2 with Y1, Y2, and Y3 in columns.
Page 23, line 319. “Fewer poor neighborhoods” means literally in French “Moins de pauvres voisinages.” I think the authors mean “Less poor” as specified in the Methods.

Discussion
18. Key results
Summarise key results with reference to study objectives.
Why explaining only significant results? Please explain. Some expected findings may have not been found due to beta risk (lack of power), but this would interest researchers to know why.

Page 23, lines 331 to 332. “associated to sedentary behavior, taking into account physical activity status.” This formulation would better apply with physical status taken as an effect-modifier (interaction term) or a confounder. Read above and if not done, explain theses options were not retained for the primary analysis.
Page 24, lines 336 to 337. “Many studies reported the consequences on physical activity levels and sedentary.” The authors posit that sedentary behavior and physical inactivity are different things. Why amalgaming here? Please, detail other study findings here to support your statement.
Page 24, line 349. “Consistent”. I am not sure this term is appropriate here. The socio-economic is the most consistent risk factor for what? Please, detail.
Page 24, lines 357 to 358. Specify here that a “better integration of communication on physical activity content” is needed if physical activity may decrease the consequences of sedentary behaviors, which is not consistent across studies, as the authors pinpoint elsewhere.
19. Limitations
Discuss both strengths and limitations of the study, taking into account sources of bias or imprecision, maybe strengths prior to limitations. Discuss both direction and magnitude of any potential bias.
20. Interpretation
Give a cautious overall interpretation of results considering objectives, limitations, multiplicity of analyses, results from similar studies, and other relevant evidence.
Page 25, line 342. There is a misinterpretation here. Write “less poor” neighborhoods as the protective effective effect discussed here applies for the fourth category of the variable which indicates a less disadvantaged neighborhood (the fifth category indicating the wealthier advantaged neighborhood).
Page 25, line 368. If possible, provide here a reference and place it after areas.
Page 25, lines 367 to 370. The sentence is not so clear. Explain what is an endogenous psychosociological mechanism.
Page 25, lines 381 to 383. The authors state that the “inhibition of some protective health-related attitudes are found in the most deprived neighborhoods”. I guess this is an interpretation but where is the study finding on which it is based on?
Page 26, line 387; I am not sure the term “intervention” is suitable here. The authors refer to multiple public health actions. Qualify the intervention as multifaceted or better use program, for instance.

21. Generalisability
Discuss the generalisability (external validity) of the study results.
Page 27, line 421. “institutionalized accommodation.” Does the authors mean long-term care facility?
Page 27, line 423. What represent the people placed in institutions on Reunion island? Are they different or representative of the general population? If not and if there are numerous, could they introduce a selection bias?
Page 27, line 424. “The accelerometer gold standard”. Is a study in nuclear physics? Reformulate with a coma and a more appropriate designation of the gold standard method.
Page 28, line 442. How a health risk could be refined? Give more precisions because it is not crystal clear.

Other information

22. Funding
Give the source of funding and the role of the funders for the present study and, if applicable, for the original study on which the present article is based.
References
Translate the French references that are accessible in Pubmed into English and write them into brackets[] as done in PubMed.

Minor discretionary corrections (request an author's reply without obligation of edits)

Title and abstract
1(*b*). Page 2, line 30. I am not sure that the prevalence is the target of primary prevention programs. Maybe incidence would be more appropriate. Prevalence decreases as a reduction of incidence.
Page 2, line 34. "to highlight… in order to …. to alleviate…" Too much "to"in the same sentence. Replace some of these by grammatically correct formulations. "aiming at + -ing".
Page 2, line 47. "Strengthening individuals." Does the authors mean enpowerment?
Page 3, lines 50 to 51. Material exposures is vague. It could be detailed.

Introduction
2. Background/rationale
Page 3, line 66. The PIMA could be named in the text as it is well known reminiscent example of the concept of disadvantaged communities regarding T2D.
Page 3, line 71. Add the acronym (SWIO) for southwestern Indian ocean and "region" after and specify it belongs to the WHO African region. This is important as Reunion island share social characteristics of both well developed and low-or-middle income countries.
Page 3, line 76. Too many wars are waged in the world. Please use a peaceful vocabulary. I do not think a combat could be waged against a probability as there is no ethics in preemptive (or preventive) wars. Rephrase using more intelligible words.
Page 3, line 81. I am not sure the results of reference #17 could be used for such generalization and the findings of a local study on Reunion island could be translated to all territories with aging populations. Complete the sentence stating this as an example with a formulation like "as a demographic study from Reunion island suggests".
Page 3, line 87. "Strengthening individuals." Does the authors mean enpowerment?
3. Objectives
State specific objectives, including any prespecified hypotheses.
Page 3, lines 91 to 92. "to highlight… in order to …. to alleviate…" Too much "to"in the same sentence. Replace some of these by grammatically correct formulations. "aiming at + -ing".

Methods
7. Variables
Confounding factors. Page 7, line 145. Age categories have been categorized differently than for sampling. It could be explained why.
8. Data sources/ measurement
Page 5, line 119. "during the study period" would be better placed upstream in the sentence, after "were gathered;"
12. Statistical methods
12 (*a*). Page 8, line 190. Remove the "the" before four categories.

Ethical considerations. I see no concern to use “concerning” twice, however it not elegant.

Results
14. Descriptive data.
Page 16, line 273. People aged 15 to 44 years could be characterized as young adults here and further, the age ranged placed in parentheses.

Discussion
19. Limitations
20. Interpretation
Page 26, line 396. Fianu et al. Replace by “we” or by ”our group.“
21. Generalisability
Discuss the generalisability (external validity) of the study results.
Page 26, line 407. “The research perspective was.” A research perspective is sensed to occur in the future. Please replace “was” by the present, the future or the conditional (is, will be, or would be…).
Page 27, line 409. “At the frontiers of…” Replace by “at the borderline” or “at the edge”, better appropriate here.

Other information
References should be placed at the end of the sentences.
Page 9, line 199. Ref#23 should be placed at the end of the sentence.
Page 9, line 211. Ref#26 should be placed at the end of the sentence.
Page 24, line 349. Ref#25 should be placed at the end of the sentence.
Page 27, line 413. Ref#22 should be placed at the end of the sentence.
Page 28, line 446. Ref#23 should be placed at the end of the sentence.
