# Supplementary material for: Determinants of adult sedentary behavior and physical inactivity for the primary prevention of diabetes in historically disadvantaged communities: A representative cross-sectional population-based study from Reunion Island
Source: PLoS One. 2024 Aug 13;19(8):e0308650. doi: 10.1371/journal.pone.0308650 (PMC11321555; doi:10.1371/journal.pone.0308650)
Supplement: Attachment — Submitted filename: Response-to-Reviewers-R1.docx [file pone.0308650.s007.docx]

Response to Reviewers Comments

**REVIEW COMMENTS TO THE AUTHOR:**

**Reviewer # 1:**

We appreciated the positive assessment of our study in the general comment and noticed that Reviewer # 1 used the STROBE checklist for observational studies (https://www.equator-network.org/reporting-guidelines/strobe/) for structuring his report. Within this framework, our responses considered specific Reviewer’s comment(s) under/after each STROBE item (we put them in Italics to better identify them). We assumed STROBE items without the Reviewer’s comment were not applicable or already presented in the text and did not expect a response from the authors. In addition, we completed the STROBE Checklist for Cross-sectional studies at the end of the responses given for Reviewer # 1.

Be careful in the following responses page and line numbers refer to the Revised Manuscript with Track Changes, except for the STROBE Checklist referring to the revised and clean manuscript for convenience.

**Specific comments / Minor compulsory revision (mandatory):**

**Point 1:**

*Title and abstract 1(a). Indicate the study’s design with a commonly used term in the title or the abstract.*

Add “population-based” to study as it is a strength of the study.

**Response 1:**

Fixed in manuscript (page 1, lines 2-3).

**Point 2:**

The abstract is too narrative and should include quantitative results.

**Response 2:**

First, we included a prevalence 95% confidence interval for both sedentary and physical inactivity behaviors (page 3, lines 42-44).

Second, to better describe significant associations between lifestyle behaviors outcomes and factors of exposure, we added significant P-values from the complete full-adjusted regression models (M3) (page 3, lines 45-47). However, to not exceed 300 words in the abstract, it was not possible to put the corresponding odds ratios and their confidence intervals estimated from the same M3 models. In addition, we had to delete two entire sentences to not exceed the 300-word threshold (Please see the new abstract pages 3-4).

**Point 3:**

*1(b).* Page 2, line 31. Replace “territory” by department, which is more exact administratively.

**Response 3:**

Fixed in manuscript (page 3, line 33).

**Point 4:**

Page 2, line 38. The authors must define the categories of interest of their multinomial outcome.

**Response 4:**

Done in manuscript (page 3, lines 41-42).

**Point 5:**

Page 2, lines 41 to 42. “… associated with sedentary or physical inactive behavior.” The formulation makes think the outcome is composite and binary while it is four categorial.

**Response 5:**

We suggest to re-use the categories introduced in Point 4. Please see (page 3, line 48).

**Point 6:**

Page 2, line 44. I am not sure a research perspective has to be placed in the abstract.

**Response 6:**

To not exceed 300 words in the abstract, we had to delete the sentence including the terms “research perspective” (Please see the new abstract pages 3-4). Thus, the amendment has become obsolete.

**Point 7:**

Page 2, line49 to page 3, line 50. Rephrase the last sentence of the abstract as “reducing” may apply for lifestyle risk factors but not T2D primary prevention programs.

**Response 7:**

“enhancing” (page 4, line 57) seems a better public health perspective to qualify T2D primary prevention programs, as stated in the Conclusion of our manuscript (page 37).

**Point 8:**

Page 2, line 49. “may suggest.” Why “may” ? The authors should better trust themselves and write “suggest” or “may foster” or something stronger like that.

**Response 8:**

Thank you for this relevant advice, which empowers our propositions. As a consequence, we deleted the word “may” (page 4, line 56).

**Point 9:**

*2. Background/rationale.Explain the scientific background and rationale for the investigation being reported.*

Page 3, lines 58 to 59. Rephrase the sentence to distinguish among the determinants of chronic diseases (such as T2D), risk factors from mediators. For instances, writing “that are intrinsically linked to both the development of sedentary lifestyle and excessive caloric intake through metabolic disturbances (insulin resistance).” Diabetes should not be placed at the end of the sentence as it is one of the chronic diseases to what the authors refer to.

**Response 9:**

Fixed in manuscript (page 4, lines 65-67).

**Point 10:**

Page 3, lines 61.”is… and is…” Two times is for the same subject, T2D. Rephrase the sentence starting with Type 2 diabetes (T2D),…[4, 5].”

**Response 10:**

Fixed in manuscript (page 4, lines 69-70).

**Point 11:**

Page 3, line 73 to page 4, line 74. Does the author use social inequalities for social inequalities of health? If yes, replace social inequalities by SIH to avoid the truism as it seems evident that differences in income and unfavorable living and working conditions define social inequalities.

**Response 11:**

We did not use “social inequalities” for social inequalities in health (SIH). In this statement, our purpose was to describe the social context of Reunion Island independent of the health state of the population.

We respectfully disagree with the comment second part (second sentence in Point 11). Indeed, SIH are also underpinned by many other social determinants than living and working conditions, which are significant causes of SIH but not the only ones. For instance, according to a widely accepted classification, the upper-stream and may be most influential determinants of health are those related to general socio-economic, cultural and environmental conditions, including economic strategies, tax policies, trade, and environmental agreements between countries (Dahlgren and Whitehead, 2021).

**Point 12:**

*3. Objectives*

*State specific objectives, including any prespecified hypotheses.*

The authors should be more explicit on their hypotheses. We guess they are presented in the long sentence preceding the objectives page 4, lines 82 to 84. They could use a formulation like “Given the abovementioned blablabla, we hypothesize … .”

**Response 12:**

As recommended, we introduced more explicitly the study’s hypothesis (page 6, lines 104-105).

**Point 13:**

They should also mention and define physical and sport activities as physical activity/inactivity is included in their multinomial four- category outcome. For readers not familiar to the literature about physical activity and health, it may be difficult to distinguish sedentary behavior and absence of physical activity.

**Response 13:**

“Physical and sport activities (PSA)” was first mentioned at the end of the second paragraph of the Introduction section. Due to the need not to unbalance the Introduction section (with a long and detailed text), we decided to develop this concept through both the WHO’s academic definition and the available proxy chosen for our analysis in the Materials and Methods section. Please see the Lifestyle behaviors outcomes section (pages 9-10, lines 190-198).

To introduce the difference and complementarity between sedentary behavior and physical inactivity, we added a statement just after the sedentary behavior definition (page 6, lines 101-102).

**Point 14:**

*Methods*

*4. Study design*

*Present key elements of study design early in the paper*

*5. Setting*

*Describe the setting, locations, and relevant dates, including periods of recruitment, exposure,*

*follow-up, and data collection.*

Page 5, line 98. Define the acronym ERPPS in the text and makes clear that the study reports this

study as it is published formerly in a study report for public health stakeholders, academics and

funders but not as a scientific publication.

**Response 14:**

These study reporting precisions were added in page 7, lines 123-124, 127-128.

**Point 15:**

Page 5, line 98. “was collected” is abrupt and let imagine it these are findings of a new study

disconnected from the ERPPS study. Better use “had been” to let see, the data had been previously

collected.

**Response 15:**

Fixed in manuscript (page 7, line 124).

**Point 16:**

Page 5, line 101. Write “aged 15 or over” as it is really “≥” and not “>”.

**Response 16:**

Fixed in manuscript (page 6, lines 121-122).

**Point 17:**

Page 5, line 107. Define the acronym “Fideli” in French in italics. Acronym should be defined in

French and English translations added when necessary.

**Response 17:**

We added the “Fideli” French definition (page 7, lines 144-145). The text already gave the English translation: “a demographic listing (for tax purposes) of accommodation and individuals”.

**Point 18:**

Page 5, line 112. Define the categories of the level of income to see how it was categorized. It will

make more intelligible the number of possible strata in the random sampling.

**Response 18:**

Done in manuscript (page 8, line 150).

**Point 19:**

*7. Variables*

*Clearly define all outcomes, exposures, predictors, potential confounders, and effect modifiers.*

*Give diagnostic criteria, if applicable*

Lifestyle behaviors outcomes. This important paragraph should be rephrased and start from

the definition of the multinomial four-category outcome, followed by clearer definitions of each of

its categories.

**Response 19:**

Done in manuscript (pages 8-10, lines 167-198).

**Point 20:**

The authors clearly have focused their hypothesis and objectives on sedentary behavior and not

physical activity. This paragraph should explain why physical activity was integrated in the outcome

definition and note taken as a potential effect-modifier (or even as a potential confounder).

**Response 20:**

Thank you for this significant methodological comment. It helped us to better justify our lifestyle behaviors outcomes. Actually, given the study design (a cross-sectional survey), it is not possible to consider that physical activity status (measured at the time of the survey) could precede the occurrence of sedentary behavior (measured at the same time) as it should be the case for a confounder or an effect-modifier. Therefore, the physical activity status is part of the outcome (see Figure 1) and is not a confounder or an effect-modifier.

This explanation is consistent with the French National Observatory on Physical Activity and Sedentariness (ONAPS) recommendation. Please see online:

- Figure 1 at:

<https://onaps.fr/les-definitions/>

- And also:

<https://lnkd.in/dNv3jpbJ>

In our manuscript, to avoid confusion, we updated the title (page 1), short title (page 1), abstract (pages 3-4, lines 31, 35-36) and main text, including “physical inactivity” in the study rationale (page 6, lines 101-102, 107) and objective (line 114).

**Point 21:**

Page 6, line 134. Physical inactivity is a composite variable which combined four reported outcome

variables.

**Response 21:**

Fixed in manuscript (page 9, line 194).

**Point 22:**

Page 6, line 137. Remove “reporting” as it has been specified previously that data were selfreported.

**Response 22:**

Fixed in manuscript (page 10, line 196).

**Point 23:**

Confounding factors. Page 7, lines 147 to 148. The sentence is confusing. How a health-related

behavior like a sedentary behavior could confound sedentary behavior?

**Response 23:**

Thank you for this comment. It was a mistake. We updated the statement (page 11, lines 203-204).

**Point 24:**

Factors of exposure. Page 7, line 158. Replace “threshold” by “categories” as we need three

thresholds or cut-off to define four categories.

**Response 24:**

We replaced the term “threshold” with “classes” (page 11, line 214) as BMI was considered an ordinal variable.

**Point 25:**

Page 7, line 160. I don’t think language spoken in the home may be defined as a psychosocial

characteristic. Please explain.

**Response 25:**

In Reunion Island, Creole is the native spoken language, whereas French is the official language taught at school and used for administrative purposes. The language spoken in the home at the age of five (in three categories: Only Creole/Only French/French and Creole/Other languages) was considered by our team as a proxy of both literacy (including health literacy) and community belonging. According to this assumption, we classified this factor within the broad category of psychosocial determinants of health.

To clarify, we stated our assumption (page 11, lines 217-218).

**Point 26:**

Page 7, lines 179 to 182. Define the referent category as zero category and the categorization 0 to

4.

**Response 26:**

We respectfully disagree with this request. In accordance with the INSEE typology defining the large neighborhood deprivation groups (1, 2, 3, 4, and 5), we chose to apply the published figures (Besson 2018). In this way, the reader will be able to match the INSSE publication and ours without difficulty. This convention has no impact on the referent category for the multinomial logistic regression model.

**Point 27:**

*8. Data sources/ measurement*

*For each variable of interest, give sources of data and details of methods of assessment*

*(measurement). Describe comparability of assessment methods if there is more than one group.*

Page 6, line 124. Which ecological data were compiled?

**Response 27:**

The goal of this statement (“Ecological data concerning the residential environment of the participants were also compiled”) was to introduce in the Data collection section the factors of exposure characterizing the residential environment which were analyzed (page 12, lines 228-239).

To improve the connection between these two parts of the Material and Methods section, we added an invitation to read the Factors of exposure section (page 8, lines 164-165).

**Point 28:**

*9. Bias*

*Describe any efforts to address potential sources of bias*

*10. Study size*

Explain why this study did not require sample size pre-specification (instead of how the study size

was arrived at).

**Response 28:**

As now stated in the Study design and Settings section (page 7, lines 128-129), the present study is a secondary analysis of the ERPPS database (available on request at: <https://data.progedo.fr/studies/doi/10.13144/lil-1584>). In other words, it was not appropriate to calculate (prior) study size. Given this framework, our study did not match the sample size pre-specification as defined by Vandenbroucke and collaborators for the STROBE guidelines. To clarify, we added a new methodological section named “Study size” just before the Statistical analysis section (see pages 12-13, lines 241-246).

**Point 29:**

*11. Quantitative variables*

*Explain how quantitative variables were handled in the analyses. If applicable, describe which*

*groupings were chosen and why?*

*12. Statistical methods*

*12 (a). Describe all statistical methods, including those used to control for confounding.*

*12 (b). Describe any methods used to examine subgroups and interactions.*

It should be explained why physical activity was integrated into the multinomial four-category

outcome as it precludes study physical activity as an effect-modifier or a confounder.

**Response 29:**

In the same way as we answered Point 20, given the study design (a cross-sectional survey), it is not possible to consider that physical activity status (measured at the time of the survey) could precede the occurrence of sedentary behavior (measured at the same time) as it should be the case for a confounder or an effect-modifier. Therefore, the physical activity status is part of the multinomial four-category outcome (see Figure 1) and is not a confounder or an effect-modifier.

This explanation is consistent with the French National Observatory on Physical Activity and Sedentariness (ONAPS) recommendation. Please see online:

- Figure 1 at:

<https://onaps.fr/les-definitions/>

- And also:

<https://lnkd.in/dNv3jpbJ>

**Point 30:**

*12 (c). Explain how missing data were addressed.* This paragraph page 9, lines 205 to 215 is

The paragraph page 9, lines 205 to 212 is difficult to read for non-statistician readers and has little

impact on the understanding the article. I would shorten it and merely explain that there were

multiple imputations.

**Response 30:**

This significant paragraph deals with the methods used to address selection biases due to missing data in the questionnaire and the subjects’ selection from the sample frame. Given the Reviewer # 2 general comment (“The study is described by the authors with adequate statistical analysis and at a good technical level, in sufficient detail.”), we believe it would be better to keep at all this paragraph. Otherwise, the reader could need information on the impact of the 49% participation rate on the results and how the statistical analysis addressed it. Please see the Reviewer # 2 specific comment (Point 9) and our response (Response 9).

For addressing the questionnaire missing data, we did not use multiple imputation but simple imputation.

**Point 31:**

Page 9, line 200. “The working framework.” Working could be replaced by operating or something

like that.

**Response 31:**

Thank you for this comment. Indeed, the word “working” was not necessary since this framework was qualified as “theoretical” in the previous sentence (page 13, line 261). Therefore, we deleted this word (line 263).

**Point 32:**

Ethical considerations. Page 10, lines 234 to 235. Explain why minors 15 to 17 were not asked to

provide their oral consent along with that of their parents.

**Response 32:**

On the contrary, minors 15 to 17 were asked to provide their oral consent along with that of their parent. To clarify, we described this process in the time frame: from the parent-consent’s step to the minor-consent’s step (page 15, lines 298-300).

**Point 33:**

*Results*

*13. Participants*

*13 (a).*Page 11, line 241. Remove the “with” and reformulate using a formulation like “which represented” or something like that.

**Response 33:**

Fixed in manuscript (page 16, lines 307-308).

**Point 34:**

*14. Descriptive data*

*14 (a). Give characteristics of study participants (eg demographic, clinical, social) and information on exposures and potential confounders.*

Page 11, lines 247 to 251. Although, Table 1 is a descriptive table, were there some comparisons between the study sample and census data. When percentages differed it could have shed light on potential selection biases? (even though in this case differences are significant due to statistical power.

**Response 34:**

We did not compare the study sample and census data using statistical tests, given the reason stated by the Reviewer. Indeed, all the ERPPS data are weighted to describe our results at a regional level by using a population-based study with individuals drawn by random sampling methods. In other words, the ERPPS weighted data and the census data are at the same level of inference provided that population criteria are similar between ERPPS and census (e.g., same age range). Within this framework, census data helped to evaluate the ERPPS overall representativeness rather than selection bias related to study non-participation.

To identify selection biases, we compared ERPPS participants versus ERPPS non-participants on socio-economic and demographic characteristics available within the 2020-Fidéli sample frame for individuals aged ≥ 15 years who were not living in a community on Reunion Island (N = 728,930 – see Figure 2). The three factors associated with participation in the ERPPS survey were sex, age and place of birth (page 16, lines 308-309).

**Point 35:**

Page 16, lines 273 to 274. Most participants possessed an educational degree. This could have been a source of selection bias. Explain and specify if there was or there wasn’t a selection bias and how it was accounted if it had an impact on study findings.

**Response 35:**

The second column of Table 1 shows that most ERPPS participants possessed an educational degree (i.e., a final secondary school diploma or above): 39.0% [95%CI = 36.4 to 41.6]. In Table 1's third column, the corresponding figure (from the 2019 census data for ≥ 15 years old) is 36.5%. We noticed that the 95% confidence interval of the former estimate includes the latter estimate. Given this observation, there was no representativeness gap in educational degrees for ERRPS compared to the Reunion Island population of the same age range and no impact on study findings was expected.

**Point 36:**

Page 16, lines 274 to 275. Define a positive perception.

**Response 36:**

A positive perception of the personal and parental history of PSA (Yes/No) is a composite factor with a 96-word definition. To avoid text unbalance between the chapters of the main text, we decided to put this long definition in an appendix (please see S2 Appendix) quoted at the end of the Confounding factors section (page 11, lines 206-207).

**Point 37:**

Page 16, line 281. What for students?.

**Response 37:**

The relationship with students was given at the end of the following sentence: “[…], and students were more likely to have sedentary/inactive profile.” (Please see page 21, line 350).

**Point 38:**

*14 (b). Indicate number of participants with missing data for each variable of interest*

*15. Outcome data*

*15 (c). Cross-sectional study—Report numbers of outcome events or summary measures*

*16. Main results*

*16 (a). Unadjusted estimates could be placed as supporting information(supplemental online tables). Make clear which confounders were adjusted for and why they were included.*

It is inappropriate and very confusing to report a single analysis on three different tables. Tables 2 to 4 should be merged into a single table presenting the M3 final model distinguishing each outcome category (Y1 in place of M1, Y2 in place of M2, as study findings that are interpreted are only those of M3 models. Results of nested models M1 and M2 models which are not interpreted or very simply in short sentences, should be presented as supporting information and supplemental online tables.

**Response 38:**

As recommended, we reshaped the former Table 2, Table 3, and Table 4 into a single table (Please see new Table 2 – pages 27-28), only showing the M3 model results. For M1 and M2, we pooled them for each risk profile leading into a new supplemental online table (Please see S1 Table). As a consequence, we updated the tables’ quotation within the main text.

**Point 39:**

Tables 2 to 4. With respect to the prespecified hypotheses, why not having taken “final secondary school diploma or above as referent category?

**Response 39:**

The choice of the reference category for the highest education degree factor was not based on a prespecified hypothesis, but on our data. In the bivariate analysis (data not shown), we found that the prevalence of participants with no diploma or primary level of education was lower among sedentary participants compared to non-sedentary participants. Moreover, we found that the prevalence of participants with a final secondary school diploma or above was higher among sedentary participants compared to non-sedentary participants. Given this significant relationship, we decided on the lowest education category as the reference category for the logistic regression models (see Table 2, S1 Table, and S2 Table).

**Point 40:**

*16 (b). Report category boundaries when continuous variables were categorized*

*16 (c). If relevant, consider translating estimates of relative risk into absolute risk for a meaningful time period*

*17 Other analyses*

*Report other analyses done—eg analyses of subgroups and interactions, and sensitivity analyses.*

Page 16, lines 264 to 267. Explain why results have not been presented at least for some microregions that shaped or contrasted the overall study findings.

**Response 40:**

This comment overlaps with that of Reviewer # 2 (Point 11).

These other analyses (according to STROBE item # 17) go beyond the scope of the present study. Indeed, the study objective was to generalize the findings at the *regional level* to propose local public health interventions. Moreover, as stated in the new Study size section, stratified multivariable regression analyses were not anticipated in the study design (page 12, lines 243-244), meaning that their findings might arise by chance given the multiplicity of analyses and inflation of type 1 error [Peacock J, Peacock P. Oxford Handbook of Medical Statistics. Oxford University Press. Oxford; 2011. 517 p]. Even so, we tried to analyze the first-order interaction between the three socio-economic and environmental factors included in the M3 multivariable regression model (i.e., rate of the artificial cover of the ground around the residence, deprivation level of the large neighborhood of residence and administrative micro-region of residence). Nevertheless, given a low number of observations within some cell(s) of the cross-tables stratified by the four categories of the dependent variable (Sedentary/Inactive, Sedentary/Active, Non-sedentary/Inactive, Non-sedentary/Active), we were able to compute only one regression model including the rate of artificial cover of the ground around the residence and the administrative micro-region of residence. The joint test gave a non-significant first-order interaction term between these two factors (P=0.8004). Thus, the multivariable regression modelling stratified by micro-region certainly lacked statistical power.

Taking into consideration all these methodological constraints and limitations, we suggest interpreting the northern region prevalence results in the Discussion section using our knowledge of the local setting (page 33, lines 458-464).

**Point 41:**

Page 23, line 323. Present crude unadjusted analyses like the primary analysis: M1 with Y1,Y2 and Y3 in columns, M2 with Y1, Y2, and Y3 in columns.

**Response 41:**

On behalf of all the authors, I was not sure to understand this comment. Indeed, there is no M1 and M2 multivariable regression models for crude unadjusted analyses. All crude OR (with 95%CI and Pvalue) were presented in a single table (with three risk profiles in columns), as shown in the supporting information (Please see S2 Table).

For convenience, we pooled M1 and M2 into a new supplemental table: S1 Table.

**Point 42:**

Page 23, line 319. “Fewer poor neighborhoods” means literally in French “Moins de pauvres voisinages.” I think the authors mean “Less poor” as specified in the Methods.

**Response 42:**

Fixed in manuscript (page 30, line 392).

**Point 43:**

*Discussion*

*18. Key results*

*Summarise key results with reference to study objectives.*

Why explaining only significant results? Please explain. Some expected findings may have not been found due to beta risk (lack of power), but this would interest researchers to know why.

**Response 43:**

As stated in the new Study size section (page 12, lines 242-243), the ERPPS database secondary analysis was made *a posteriori*. Given this framework, the study did not match the sample size pre-specification necessary for identifying with adequate statistical power some known risk factors. In this paper, we adopted an exploratory approach as a first step to explore the relationships between various characteristics of the participants and their environment and physical inactivity and sedentariness, and thus identify potential determinants of physical inactivity and sedentariness. A perspective to the present work would be to use our first results to develop and explore specific hypotheses using an explanatory approach. We acknowledged this new study limitation, followed by a research perspective in the Discussion section (page 36, lines 541-545).

**Point 44:**

Page 23, lines 331 to 332. “associated to sedentary behavior, taking into account physical activity status.” This formulation would better apply with physical status taken as an effect-modifier (interaction term) or a confounder. Read above and if not done, explain theses options were not retained for the primary analysis.

**Response 44:**

In line with our previous responses (see Reviewer # 1’s Points 20 and 29), we acknowledge this formulation was inappropriate. We did not want to consider the physical activity status as an effect-modifier (interaction term) or a confounder. To clarify, we propose to precise the risk profiles (outcomes) highlighted in our study (page 31, lines 406-407).

**Point 45:**

Page 24, lines 336 to 337. “Many studies reported the consequences on physical activity levels and sedentary.” The authors posit that sedentary behavior and physical inactivity are different things. Why amalgaming here? Please, detail other study findings here to support your statement.

**Response 45:**

In the first two sentences of the paragraph, we didn’t want to discuss the two quoted studies (Chaabna et al. 2022 and Mehraeen et al. 2023). It was a broad statement on the COVID-19 pandemic impact on physical activity and sedentariness. This general topic aimed to introduce Chêne’s article (2022), which we wanted to discuss in detail. Indeed, Chêne’s study was implemented the same year as ours (2021) and gave focuses on two aspects of our interested lifestyle behaviors: the report of insufficient physical activity levels below the guidelines, on one hand, and the report of staying seated more than seven hours/day (an indicator of sedentary behavior), on the other hand. We think this relevant study supported our findings related to the COVID-19 pandemic.

**Point 46:**

Page 24, line 349. “Consistent”. I am not sure this term is appropriate here. The socio-economic is the most consistent risk factor for what? Please, detail.

**Response 46:**

Thank you for this comment. Our first proposition was unclear. We reformulated here (page 31, lines 425-428).

**Point 47:**

Page 24, lines 357 to 358. Specify here that a “better integration of communication on physical activity content” is needed if physical activity may decrease the consequences of sedentary behaviors, which is not consistent across studies, as the authors pinpoint elsewhere.

**Response 47:**

This comment overlaps with that of Reviewer # 2 (Point 16), therefore we proposed a synthesis taking into consideration the two comments (page 32, lines 436-439).

**Point 48:**

*19. Limitations*

*Discuss both strengths and limitations of the study, taking into account sources of bias or imprecision, maybe strengths prior to limitations. Discuss both direction and magnitude of any potential bias.*

*20. Interpretation*

*Give a cautious overall interpretation of results considering objectives, limitations, multiplicity of analyses, results from similar studies, and other relevant evidence.*

Page 25, line 342. There is a misinterpretation here. Write “less poor” neighborhoods as the protective effective effect discussed here applies for the fourth category of the variable which indicates a less disadvantaged neighborhood (the fifth category indicating the wealthier advantaged neighborhood).

**Response 48:**

To match with the text, we assumed that line 362 (instead of 342) was pointed. Thank you for this comment. We made the correction here (page 32, line 443).

**Point 49:**

Page 25, line 368. If possible, provide here a reference and place it after areas.

**Response 49:**

Thank you for this recommendation. We added the reference to the neighborhood deprivation typology from the INSEE for defining the most deprived living areas (page 32, line 451).

**Point 50:**

Page 25, lines 367 to 370. The sentence is not so clear. Explain what is an endogenous psychosociological mechanism.

**Response 50:**

The concept of endogenous psychosocial mechanism/exposure is explained two paragraphs further within the section dedicated to the typology of biological mechanisms involved in social exposures embodiment (Kelly-Irving and Delpierre, 2021). For more explanations, we invited the reader to go there (page 32, lines 451-452).

**Point 51:**

Page 25, lines 381 to 383. The authors state that the “inhibition of some protective health-related attitudes are found in the most deprived neighborhoods”. I guess this is an interpretation but where is the study finding on which it is based on?

**Response 51:**

This interpretation was based on the study findings from Table 2 at the intersection of the column named “Sedentary/Active profile” and the line of level 4 within “Deprivation level of the large neighborhood of residence”:

aOR = 0.55 [95%CI = 0.32 to 0.94] P=	0.030

In other words (as stated page 30, lines 392-394): Those in “less poor neighborhoods located far from city centers” (Level 4) were less likely to have a sedentary/active profile compared to participants in “urban neighborhoods facing multiple socio-economic difficulties” (Level 1).

To interpret in the way, we did in the Discussion section, we reversed the reference category (Level 1 → Level 4) leading to: Participants in “urban neighborhoods facing multiple socio-economic difficulties” (Level 1) were **more** likely to have a sedentary/active profile compared to those in “less poor neighborhoods located far from city centers” (Level 4).

**Point 52:**

Page 26, line 387; I am not sure the term “intervention” is suitable here. The authors refer to multiple public health actions. Qualify the intervention as multifaceted or better use program, for instance.

**Response 52:**

We acknowledged the multiplicity of actions that could be implemented. To be consistent with the previous sentence (page 34, line 482), we propose to re-use the same term: “actions” (line 483).

**Point 53:**

*21. Generalisability*

*Discuss the generalisability (external validity) of the study results.*

Page 27, line 421. “institutionalized accommodation.” Does the authors mean long-term care facility?

**Response 53:**

The institutionalized accommodations (we put a “s”) include long-term care facilities among the other structures enumerated. To complete the listing, we added this example here (page 35, line 528).

**Point 54:**

Page 27, line 423. What represent the people placed in institutions on Reunion island? Are they different or representative of the general population? If not and if there are numerous, could they introduce a selection bias?

**Response 54:**

To the best of our knowledge, it is hard to estimate the exact number of people living in institutions on Reunion Island at the time of the ERRPS survey (in late 2021). Indeed, there is not one but numerous institutions depending on the need to address: care (public/private), simple accommodation (short-term / mid-term / long-term), social aid (many solutions are proposed by the General Council of Reunion), etc... Moreover, those publics are not captive populations. People can enter and then exit from an institution, so the number fluctuates across the calendar month of the year (seasonality). Given the prevalence of both social vulnerability (around 40%) and the elderly population ≥60 years (~17%), we can hypothesize that less than 10% of the general population is concerned by such a placement.

Whatever the case, such populations are not representative of the general population considering sedentary behavior. These groups are undoubtedly affected by problems linked to age, chronic diseases, limitations, disabilities, deprivation, so they spend more time in bed or sitting up during the day, increasing the sedentary time compared to younger, healthier and non-deprived people.

As stated in the manuscript (page 36, line 529), the absence of such populations living in institutionalized accommodations in the ERPPS sample was (not a selection bias but) a lack of general population coverage due to the study population eligibility criteria: Participants were included if they were ≥15 years old, and were residents living in ordinary accommodation on Reunion Island in 2021.

We added a short clarification about the generalizability of the study descriptive results here (lines page 36, lines 529-530).

**Point 55:**

Page 27, line 424. “The accelerometer gold standard”. Is a study in nuclear physics? Reformulate with a coma and a more appropriate designation of the gold standard method.

**Response 55:**

Fixed in manuscript (page 36, line 531).

**Point 56:**

Page 28, line 442. How a health risk could be refined? Give more precisions because it is not crystal clear.

**Response 56:**

To make it easier to comprehend, we replaced the word “refine” with “rank” and specified the two-opposite risk-levels: “sedentary and physically inactive profile” Versus “non-sedentary and physically active profile” (page 37, lines 556-558).

**Point 57:**

*Other information*

*22. Funding*

*Give the source of funding and the role of the funders for the present study and, if applicable, for the original study on which the present article is based.*

References

Translate the French references that are accessible in Pubmed into English and write them into brackets[] as done in PubMed.

**Response 57:**

This amendment was applicable for only one reference (page 42, lines 730-732):

Fianu A, Villeval M, Naty N, Favier F, Lang T. [To analyse the transferability of an intervention : applying the functions/implementation/context model on a diabetes prevention programme]. Santé Publique. 2017;Vol. 29:525–34. https://doi.org/10.3917/spub.174.0525.

**Specific comments / Minor discretionary corrections (request an author’s reply without obligation of edits):**

**Point 58:**

*Title and abstract 1(b).*

Page 2, line 30. I am not sure that the prevalence is the target of primary prevention programs. Maybe incidence would be more appropriate. Prevalence decreases as a reduction of incidence.

**Response 58:**

Thank you for this amendment. We replaced “prevalence” with “incidence” (page 3, line 32).

**Point 59:**

Page 2, line 34. “to highlight… in order to …. to alleviate…” Too much “to”in the same sentence. Replace some of these by grammatically correct formulations. “aiming at + -ing”.

**Response 59:**

Fixed in manuscript (page 3, line 37).

**Point 60:**

Page 2, line 47. “Strengthening individuals.” Does the authors mean enpowerment?

**Response 60:**

We didn’t mean empowerment, but interventions targeting individuals. According to the typology of actions to tackle social inequalities in health (M. Whitehead, 2007), “Strengthening individuals” refers to interventions aiming at strengthening individuals in disadvantaged circumstances and using person-based strategies (such as health education programs). To clarify this point, we added between brackets the strategy of this type of intervention (page 4, lines 54-55).

**Point 61:**

Page 3, lines 50 to 51. Material exposures is vague. It could be detailed.

**Response 61:**

This terminology was adapted from the framework proposed by M. Kelly-Irving and C. Delpierre for understanding health inequalities over the life course (M. Kelly-Irving and C. Delpierre, 2021). According to this framework, one can distinguish three main types of factors: structural, social and psychosocial factors.

In our case, “material” exposures referred to education, professional status and limited access to material resources in the neighborhood. However, to ensure consistency with the framework used, we replaced the term “material” with “structural” in the abstract (page 4, line 58) and in the discussion section of the manuscript (page 33-34, lines 472, 477, and 481).

**Point 62:**

*Introduction*

*2. Background/rationale*

Page 3, line 66. The PIMA could be named in the text as it is well known reminiscent example of the concept of disadvantaged communities regarding T2D.

**Response 62:**

Done in manuscript (page 5, line 77).

**Point 63:**

Page 3, line 71. Add the acronym (SWIO) for southwestern Indian ocean and “region” after and specify it belongs to the WHO African region. This is important as Reunion island share social characteristics of both well developed and low-or-middle income countries.

**Response 63:**

Done in manuscript (page 5, lines 85-87).

**Point 64:**

Page 3, line 76. Too many wars are waged in the world. Please use a peaceful vocabulary. I do not think a combat could be waged against a probability as there is no ethics in preemptive (or preventive) wars. Rephrase using more intelligible words.

**Response 64:**

We replaced the verb “combat” with “reduce” (page 5, line 95).

**Point 65:**

Page 3, line 81. I am not sure the results of reference #17 could be used for such generalization and the findings of a local study on Reunion island could be translated to all territories with aging populations. Complete the sentence stating this as an example with a formulation like “as a demographic study from Reunion island suggests”.

**Response 65:**

We completed this sentence as proposed by the Reviewer (page 6, lines 102-104).

**Point 66:**

Page 3, line 87. “Strengthening individuals.” Does the authors mean enpowerment?

**Response 66:**

We didn’t mean empowerment, but interventions targeting individuals. According to the typology of actions to tackle social inequalities in health (M. Whitehead, 2007), “Strengthening individuals” refers to interventions aiming at strengthening individuals in disadvantaged circumstances and using person-based strategies (such as health education programs).

In the same way, we did in the abstract, to clarify this point, we added between brackets the strategy of this type of intervention (page 6, lines 111-112).

**Point 67:**

*3. Objectives*

*State specific objectives, including any prespecified hypotheses.*

Page 3, lines 91 to 92. “to highlight… in order to …. to alleviate…” Too much “to”in the same sentence. Replace some of these by grammatically correct formulations. “aiming at + -ing”.

**Response 67:**

Fixed in manuscript (page 6, line 116).

**Point 68:**

*Methods*

*7. Variables*

Confounding factors. Page 7, line 145. Age categories have been categorized differently than for

sampling. It could be explained why.

**Response 68:**

For statistical analysis purposes, at the bivariate and then multivariate analysis stage, we had to use larger age categories than for sampling to prevent a low number of observations within some cell(s) of cross-tables, statistical power limitation and numerical problems such as model non-convergence.

**Point 69:**

*8. Data sources/ measurement*

Page 5, line 119. “during the study period” would be better placed upstream in the sentence, after

“were gathered;”

**Response 69:**

Fixed in manuscript (page 8, line 159).

**Point 70:**

*12. Statistical methods*

*12 (a).* Page 8, line 190. Remove the “the” before four categories.

**Response 70:**

Fixed in manuscript (page 13, line 254).

**Point 71:**

Ethical considerations. I see no concern to use “concerning” twice, however it not elegant.

**Response 71:**

We replaced the second “concerning” with “about” (page 15, line 288)

**Point 72:**

*Results*

*14. Descriptive data.*

Page 16, line 273. People aged 15 to 44 years could be characterized as young adults here and further, the age ranged placed in parentheses.

**Response 72:**

Thank you for this proposition, but we prefer to let the reader interpret whether the 15-44 age category characterized young adults. Indeed, we think this interpretation is context-dependent and population-based: some 15-44-year-old populations could be considered as young adults, while others would be assimilated as a mix of young and middle-aged adults.

**Point 73:**

*Discussion*

*19. Limitations*

*20. Interpretation*

Page 26, line 396. Fianu et al. Replace by “we” or by ”our group.“

**Response 73:**

Fixed in manuscript (page 34, line 493).

**Point 74:**

*21. Generalisability*

*Discuss the generalisability (external validity) of the study results.*

Page 26, line 407. “The research perspective was.” A research perspective is sensed to occur in the future. Please replace “was” by the present, the future or the conditional (is, will be, or would be…).

**Response 74:**

Fixed in manuscript (page 35, line 510).

**Point 75:**

Page 27, line 409. “At the frontiers of…” Replace by “at the borderline” or “at the edge”, better appropriate here.

**Response 75:**

Fixed in manuscript (page 35, line 512).

**Point 76:**

*Other information*

References should be placed at the end of the sentences.

Page 9, line 199. Ref#23 should be placed at the end of the sentence.

Page 9, line 211. Ref#26 should be placed at the end of the sentence.

Page 24, line 349. Ref#25 should be placed at the end of the sentence.

Page 27, line 413. Ref#22 should be placed at the end of the sentence.

Page 28, line 446. Ref#23 should be placed at the end of the sentence.

**Response 76:**

Fixed in manuscript (page 13, line 263, page 14, line 275, page 31, line 428, page 35, line 519, page 37, line 564).

STROBE Statement—Checklist of items that should be included in reports of ***cross-sectional studies***

|  | Item No | Recommendation | Page number, table, figure |
| --- | --- | --- | --- |
| **Title and abstract** | 1 | (*a*) Indicate the study’s design with a commonly used term in the title or the abstract | Pages 1-2 |
|  |  | (*b*) Provide in the abstract an informative and balanced summary of what was done and what was found | Pages 2-3 |
| Introduction | | |  |
| Background/rationale | 2 | Explain the scientific background and rationale for the investigation being reported | Pages 3-5 |
| Objectives | 3 | State specific objectives, including any prespecified hypotheses | Pages 4-5 |
| Methods | | |  |
| Study design | 4 | Present key elements of study design early in the paper | Pages 5-6 |
| Setting | 5 | Describe the setting, locations, and relevant dates, including periods of recruitment, exposure, follow-up, and data collection | Pages 5-6 |
| Participants | 6 | (*a*) Give the eligibility criteria, and the sources and methods of selection of participants | Pages 5-6 |
| Variables | 7 | Clearly define all outcomes, exposures, predictors, potential confounders, and effect modifiers. Give diagnostic criteria, if applicable | Figure 1, pages 7-9, S2 Appendix |
| Data sources/ measurement | 8* | For each variable of interest, give sources of data and details of methods of assessment (measurement). Describe comparability of assessment methods if there is more than one group | Pages 7-9, S2 Appendix |
| Bias | 9 | Describe any efforts to address potential sources of bias | S1 Figure |
| Study size | 10 | Explain how the study size was arrived at | Pages 9-10 |
| Quantitative variables | 11 | Explain how quantitative variables were handled in the analyses. If applicable, describe which groupings were chosen and why | Pages 7-9, S2 Appendix |
| Statistical methods | 12 | (*a*) Describe all statistical methods, including those used to control for confounding | Pages 10-11, S1 Figure (legend) |
|  |  | (*b*) Describe any methods used to examine subgroups and interactions | Not applicable (study size limitation) |
|  |  | (*c*) Explain how missing data were addressed | Page 11 |
|  |  | (*d*) If applicable, describe analytical methods taking account of sampling strategy | Page 11 |
|  |  | (*e*) Describe any sensitivity analyses | S1 Table (M1, M2) |
| Results | | |  |
| Participants | 13* | (a) Report numbers of individuals at each stage of study—eg numbers potentially eligible, examined for eligibility, confirmed eligible, included in the study, completing follow-up, and analysed | Figure 2, page 13 |
|  |  | (b) Give reasons for non-participation at each stage | Figure 2 |
|  |  | (c) Consider use of a flow diagram | Figure 2 |
| Descriptive data | 14* | (a) Give characteristics of study participants (eg demographic, clinical, social) and information on exposures and potential confounders | Table 1, pages 13 and 18 |
|  |  | (b) Indicate number of participants with missing data for each variable of interest | Not applicable (simple imputation) |
| Outcome data | 15* | Report numbers of outcome events or summary measures | Not applicable (weighted data) |
| Main results | 16 | (*a*) Give unadjusted estimates and, if applicable, confounder-adjusted estimates and their precision (eg, 95% confidence interval). Make clear which confounders were adjusted for and why they were included | S2 Table, Table 2, pages 18 and 21 |
|  |  | (*b*) Report category boundaries when continuous variables were categorized | Table 1, Table 2, S2 Table |
|  |  | (*c*) If relevant, consider translating estimates of relative risk into absolute risk for a meaningful time period | Not applicable (cross-sectional study) |
| Other analyses | 17 | Report other analyses done—eg analyses of subgroups and interactions, and sensitivity analyses | Figure 3, S1 Table |
| Discussion | | |  |
| Key results | 18 | Summarise key results with reference to study objectives | Pages 21-22 |
| Limitations | 19 | Discuss limitations of the study, taking into account sources of potential bias or imprecision. Discuss both direction and magnitude of any potential bias | Pages 26-27 |
| Interpretation | 20 | Give a cautious overall interpretation of results considering objectives, limitations, multiplicity of analyses, results from similar studies, and other relevant evidence | Pages 22-25 |
| Generalisability | 21 | Discuss the generalisability (external validity) of the study results | Page 26 |
| Other information | | |  |
| Funding | 22 | Give the source of funding and the role of the funders for the present study and, if applicable, for the original study on which the present article is based | Cover letter and Plos One’s online submission platform |

*Give information separately for exposed and unexposed groups.

**Note:** An Explanation and Elaboration article discusses each checklist item and gives methodological background and published examples of transparent reporting. The STROBE checklist is best used in conjunction with this article (freely available on the Web sites of PLoS Medicine at http://www.plosmedicine.org/, Annals of Internal Medicine at http://www.annals.org/, and Epidemiology at http://www.epidem.com/). Information on the STROBE Initiative is available at www.strobe-statement.org.

**Reviewer # 2:**

We are grateful for the review, which helped us to improve the quality of the manuscript. We thank the Reviewer # 2 for showing interest in our study and the constructive comments. We carefully addressed all points and modified the manuscript accordingly.

Please, be careful in the following responses page and line numbers refer to the Revised Manuscript with Track Changes.

I have the following questions and comments regarding the manuscript:

**Point 1:**

Introduction:

- The text discusses the impact of socio-economic changes on health behavior and type 2 diabetes

(T2D). Although the connections are clear, the logical process of the text should be made clearer so

that readers can follow the argument more easily. Showing the connections, for example how

socio-economic changes lead to a higher prevalence of T2D, may require a more detailed

explanation.

**Response 1:**

Thank you for this comment. We acknowledge that our first explanations were too brief. In the revised manuscript, we elaborated on two possible mechanisms for explaining the impact of socio-economic changes and environmental changes on health behavior and T2D (page 5, lines 79-83).

**Point 2:**

- The information about the island of Réunion is detailed and relevant, but the history of the text

does not make it clear why this specific location is important. It would be worthwhile to emphasize

more why this particular community is being studied and how it differs from other communities in

a similar socio-economic situation.

**Response 2:**

Thank you again for this helpful comment. As suggested, we completed the description of the Réunion Island population characteristics (multiethnic/multicultural settlement). We added more information about the island location in response to the Reviewer # 1 comment (please see Point 63). Furthermore, we stated on a social particularity (‘Living together’) which could have implications for the public health interventions based on community strengthening processes such as local-networks (Please see page 34, lines 492-496 in the Discussion section).

Please see the amendments on page 5, lines 85-91.

**Point 3:**

- A more precise definition of terms such as "contextual influences" and "macro- or mesoeconomic and social factors" could help readers better understand the content of the text.

**Response 3:**

We agreed that these terms were not explicit and needed clarification. Except for “contextual influences” (now defined between brackets) we deleted the other terms (i.e., "macro- or mesoeconomic and social factors") and gave instead definitions in first intention (page 4, lines 72-74 and page 5, line 75).

**Point 4:**

The text mentions the island's historical context (slavery and indentured labor), but could also explain their effects on current health inequalities in more detail.

**Response 4:**

The link between past and current social inequalities now appears on page 5, lines 93-94. The expected effect of past social inequalities on current health inequalities is mentioned in lines 96-98.

**Point 5:**

Material and methods:

- When using the phrase "Identical to the nationwide study of PSA (ENPPS-2020)", it would be useful to briefly summarize the purpose and methods of the ENPPS-2020 study so that readers can better understand the relationship between the two studies.

**Response 5:**

We briefly summarized the ENPPS-2020 study purpose and stated the shared methods between the two surveys. In addition, we gave access to the official PROGEDO website for a comprehensive description of the ENPPS-2020 study methodology (page 7, lines 133-141).

**Point 6:**

- The details of the sampling methods are thorough, but the term "one-level systematic random sampling stratified by the micro-region" may require an explanation as to exactly how the sampling took place.

**Response 6:**

To explain the sampling method, we described what was done using an implementation perspective (page 8, lines 150-153).

**Point 7:**

- Mentioning the unequal sampling probability is important, but it would be helpful to provide further explanation of how this is handled in the analysis (e.g., by weighting).

**Response 7:**

We added a statement about handling unequal sampling probability by weighting in the Statistical analysis section (page 14, lines 276-277).

**Point 8:**

- Mention of “positive perception of personal and parental history of PSA” as a potential confounder is important, but it is unclear how this variable was measured and what effect it may have had on the results.

**Response 8a to the first part of Point 8: Measure**

As stated page 11, lines 206-207, we defined this composite variable in an appendix (S2 Appendix. Data management and rationale). The appendix allowed us not to unbalance the Material and Methods section with detailed text.

In the S2 Appendix, we added a review of models to study and predict health-related behaviour change from Taylor and collaborators (2006). In this review, the authors reported on page 43 that in the context of exercise behavior, “the most salient behavioural belief is that exercise improves physical and psychological health; that family members have the strongest normative influence on exercise [...]”. This new reference helped us to improve the rationale for considering the “positive perception of personal and parental history of PSA” as a potential confounder regarding the relationship between sedentary behavior or physical inactivity and individual socio-economic status.

**Response 8b to the second part of Point 8: Effect**

In the ERPPS statistical analysis, the effect of “positive perception of personal and parental history of PSA” on the lifestyle behaviors outcomes was described by another appendix: S2 Table. In this supplementary table, we see that participants with no positive perception of personal and parental history of PSA were more likely to have, respectively:

Sedentary/Inactive profile (crude OR = 2.35 [95%CI: 1.43 to 3.87] P=0.001).

Non-sedentary/Inactive profile (crude OR = 14.69 [95%CI: 4.39 to 49.18] P<0.001).

This factor was not associated with the Sedentary/Active profile (P=0.988).

Beyond the theoretical rationale (see upper: first part), the observed statistical associations gave empirical concern for including this factor as a confounding factor. From here, and given the sequential approach (please see S1 Fig), we chose to include all together three confounding factors (the positive perception of the personal and parental history of PSA, age, and sex) in the basic model (M0) and the subsequent nested multivariable regression models (M1, M2, and M3). As stated in the S1 Fig legend, the objective was to control for any possible imbalance between the groups defined by the new categorical variables introduced into the subsequent M1 and M2 models. Within this framework, we presented the combined effect of the three adjustments (including the positive perception of the personal and parental history of PSA) in M1 (see S1 Table) on totally abandoning at least one PSA due to the COVID-19 pandemic compared to the non-adjusted crude OR estimate (see S2 Table).

**Point 9:**

Results:

- Regarding the sentence "In total, 2,010 adults from Reunion Island participated in the study with a participation rate of 49%", the question is how can the participation rate affect the reliability of the results in the study?

**Response 9:**

It is a critical methodological issue about the impact of selection bias in the ERPPS study. In our manuscript, the selection bias was described in the following sentence (page 16, lines 308-309): “Participants were mostly women, aged 30 to 59 years and Reunion Island natives (data not shown).”

For more explanations:

When looking at Figure 2 (flow chart), we can separate the reasons for non-participation into two broad categories: those with a likely low impact in terms of selection bias (telephone numbers not called, false telephone numbers, ineligible individuals) versus those with a likely mid-high impact in terms of selection bias (unavailable individuals (no answer or busy line), refusals, questionnaires with significant data collection failures). In the latter category, the most problematic cases are refusals and questionnaires with significant data collection failures, which represented 5.6% of the total sample frame [(223+69)/5200]. Thus, although the overall non-participation rate is elevated (51% = 100% - 49%), the specific non-participation rate undoubtedly linked to selection bias seems minimal (5.6%). This observation gave a baseline confidence in the reliability of the study results.

To go further, we addressed the total non-response (for three categories of non-participants, including those who refused to participate or abandoned before the end of the telephone interview) by data reweighting and data calibration to increase regional representativeness of PSA. For data reweighting and data calibration, we included, among other socio-economic and demographic characteristics available in the sample frame, the three factors associated with participation in the ERPPS survey: sex, age and place of birth. Lastly, all these methods to correct the impact of selection bias on the study results increased the confidence in the study's reliability.

Given these explanations, we updated the Statistical analysis section (page 14, lines 274-275) and the study strengths paragraph within the Discussion section (page 37, lines 554-555).

**Point 10:**

- During the presentation of the results, it would be good to provide a further explanation of the significance and interpretation of the confidence interval at the sentence "Over half the participants had a sedentary behavior (53.9%, 95%CI: [51.1 to 56.7])".

**Response 10:**

As suggested, we used the confidence interval interpretation to reinforce the statement reported above (page 16, lines 314-315).

**Point 11:**

- In the case of the results for administrative micro-regions ("inhabitants of the northern region had the highest prevalence estimate for both sedentary/inactive (15.0%) and sedentary/active (46.1%)"), the differences are significant, but what factors can influence them?

**Response 11:**

This comment overlaps with that of Reviewer # 1 (Point 40).

These other analyses (according to STROBE item # 17) go beyond the scope of the present study. Indeed, the study objective related to generalizing the findings at the *regional level* to propose local public health interventions. Moreover, as stated in the new Study size section, stratified multivariable regression analyses were not anticipated in the study design (page 12, lines 243-244), meaning that their findings might arise by chance given the multiplicity of analyses and inflation of type 1 error [Peacock J, Peacock P. Oxford Handbook of Medical Statistics. Oxford University Press. Oxford; 2011. 517 p]. Even so, we tried to analyze the first-order interaction between the three socio-economic and environmental factors included in the M3 multivariable regression model (i.e., rate of the artificial cover of the ground around the residence, deprivation level of the large neighborhood of residence and administrative micro-region of residence). Nevertheless, given a low number of observations within some cell(s) of the cross-tables stratified by the four categories of the dependent variable (Sedentary/Inactive, Sedentary/Active, Non-sedentary/Inactive, Non-sedentary/Active), we were able to compute only one regression model including the rate of artificial cover of the ground around the residence and the administrative micro-region of residence. The joint test gave a non-significant (P=0.8004) first-order interaction term between these two factors. Thus, the multivariable regression modelling stratified by micro-region certainly lacked statistical power.

Taking into consideration all these methodological constraints and limitations, we suggest interpreting the northern region prevalence results in the Discussion section using our knowledge of the territory (page 33, lines 458-464).

**Point 12:**

- Regarding the generalizability of the results, it would be worthwhile to make further comments on how other regions can utilize the findings of the study.

**Response 12:**

We acknowledge the Reviewer for suggesting this amendment. However, as stated (page 35, lines 514-515), we think our findings are not entirely generalizable or transferable to other contexts because they highlighted social determinants which might be specific to the Reunion Island territory.

Even so, our methods for sampling, data collection, statistical analysis (the socio-ecological framework and sequential approach) (page 35, lines 519-521), and results interpretation based on the two typologies (Whitehead 2007, Kelly-Irving and Delpierre 2021) could be re-used in other contexts in future studies. We believe this approach is relevant and useful for population health intervention research (Hawe and Potvin 2009).

**Point 13:**

Discussion:

- The transition from the effects of COVID-19 to the analysis of the socio-economic background is sudden. It would be worthwhile to provide a smoother transition, for example by connecting the two topics with how socio-economic background influenced individuals' responses during COVID-19.

**Response 13:**

As suggested, we connected the two topics using a short transition (please see page 31, lines 421-422) based on our work: Health Impacts of the COVID-19 Lockdown Measure in a Low Socio-Economic Setting: A Cross-Sectional Study on Reunion Island

**Point 14:**

- In addition to the cited statistical data (e.g. "one adult out of two" and "one adult out of five"), it would be worthwhile to provide exact percentages so that the reader can interpret the ratios more easily.

**Response 14:**

Fixed in manuscript (page 31, lines 414 and 416).

Note: in the Chêne’s article (2022), the author reported the figure for physical activity with no exact percentage as follow (English traduction): “The prevalence of lack of physical activity (around 50% in May 2020) was maintained one year later.” Please see page 2 of:

https://www.santepubliquefrance.fr/etudes-et-enquetes/coviprev-une-enquete-pour-suivre-l-evolution-des-comportements-et-de-la-sante-mentale-pendant-l-epidemie-de-covid-19/documents/enquete_coviprev_tpsassis_sup7h_vague24

**Point 15:**

- The term "sedentary and active profile" needs clarification, since "sedentary" and "active" are opposite concepts. Perhaps the term "sedentary and physically active periods" would be more appropriate.

**Response 15:**

Thank you for this clarification. We made the amendments here (page 32, lines 431-432 and 443-444).

**Point 16:**

- The relationship between higher health literacy and physical activity mentioned by Rey et al (2023) deserves a more detailed explanation. For example, how higher health literacy can improve the effectiveness of health communication and how it can help promote physical activity.

**Response 16:**

This comment overlaps with that of Reviewer # 1 (Point 47).

As suggested, we gave a more detailed explanation based on the health literacy definition (page 32, lines 436-439).

**Point 17:**

- It is sometimes difficult to follow cause-and-effect relationships between the statements in the discussion. For example, the causes of physical activity inequalities and the mechanisms leading to them are not fully understood.

**Response 17:** To improve the understanding of the cause-and-effect relationship supported by Paudel et al. (2023) and our study, we stated the expected effect of political and territorial inequalities on PSA (page 32, lines 448-450).

**Point 18:**

The term "endogenous psychosocial mechanism on the stress perception" would require more explanation so that the reader can understand how this can lead to a sedentary lifestyle.

**Response 18:**

Thank you for this comment, which made us aware of an improper statement. After checking, we didn’t want to presume an endogenous psychosocial mechanism on the stress perception but on the inhibition of some protective health-related attitudes. We corrected this statement by deleting the extra text (page 32, line 452).

In line with what was suggested by the Point 17 (first sentence), we made further explanations about this specific assumption based on the theory of planned behavior (Ajzen, 1991) (page 32-33, lines 454-456).

**Point 19:**

- The distinction between "exogenous" and "endogenous" factors is useful, but the examples in the text are not always clear. For example, "abandoning physical activity due to the COVID-19 pandemic" is presented as a behavioral factor, but it is not clear how it fits into the exogenous category.

**Response 19:**

First, we gave some examples and keywords (page 33, lines 472-475) aiming at illustrating the main categories of the typology to describe the biological mechanisms involved in social exposures embodiment (Kelly-Irving and Delpierre, 2021).

Second, according to this typology, health-related behaviors are classified within the exogeneous factors category or “mechanisms of exogeneous origin” external to the human body. To illustrate this category, Kelly-Irving and Delpierre took the SARS-CoV2 epidemic as a clear example of an exogenous entity interacting with structural factors (occupation, transport, home living conditions) to spread over the population. In our case, abandoning physical activity due to the COVID-19 pandemic was a health-related behavior determined by the context in which it happened (Please see the definition of this factor of exposure in the material and Method section on page 11, lines 210-211). To clarify, we reminded the concept of context-based behavior here (page 33, lines 475-476).

**Point 20:**

- Among the intervention proposals is the strategy of "proportionate universalism", but it is not clear how this can be implemented in practice. More detailed explanations and concrete examples would be needed.

**Response 20:**

Thank you for this request. In response, we included a definition of the proportionate universalism strategy and illustrated it with a fictive but plausible local example (pages 34-35, lines 500-505).

**Point 21:**

- The sentence "The understanding of individual and socio-environmental determinants..." is too general and not specific enough. It would be worth clarifying what specific interventions are possible with such knowledge.

**Response 21:**

To precise the application with such knowledge, we stated and argued for the local public health interventions here (page 35, lines 507-509).

**Point 22:**

- The "neighborhood deprivation level indicator" is specific to the island of Réunion, but it is not clear why and how it differs from indicators used elsewhere. A more detailed explanation would help the reader understand the differences.

**Response 22:**

We reported a more detailed explanation here (page 35, lines 515-519).

**Point 23:**

- After the comment "multivariate analyzes did not include all the environmental factors", it would be worthwhile to clarify what specific environmental factors are missing and how they may affect the results.

**Response 23:**

To clarify this statement, we gave some examples of missing environmental factors based on the O’Donoghue et al. review and outlined the residual confounding limitation (page 36, lines 536-541).

* * *
